# Quantifying Base-Dependent Lewis Acidity in Substituted Boranes through DFT and Machine Learning

**DOI:** 10.1021/acs.inorgchem.6c04676

**Published:** 2026-10-19

**Authors:** Amir Mahdian, Kari Laasonen, Kaveh Farshadfar

**Affiliations:** Department of Chemistry and Materials Science, School of Chemical Engineering, 174277 Aalto University, 02150 Espoo, Finland

## Abstract

The dependence of Lewis acidity on the interacting base is established. Here, density functional theory calculations and machine-learning analysis quantify substituent effects and base dependence within a combinatorial library of 816 trisubstituted boranes constructed from 16 substituents. Interactions with five probe bases (H_2_O, NH_3_, C_5_H_5_N, PMe_3_, and Cl^–^) yield 3927 calculated adduct formation free energies in dichloromethane. Electron-withdrawing substituents generally enhance borane Lewis acidity, whereas electron-donating substituents attenuate it. The magnitude of these effects varies with the base, producing changes and occasional reversals in relative acidity rankings. PMe_3_ shows the strongest dependence on substituent identity among the investigated bases. Weak linear correlations between isolated-borane global hardness and adduct formation free energy indicate limited predictive power for this single descriptor. Random forest models describe the computed trends, while SHAP analysis identifies the summed Hammett σ_*p*_ parameter, the borane–base frontier orbital energy gap, and borane electrophilicity as the most influential descriptors in the model. These results provide a quantitative map of substituent effects and the transferability of relative borane acidity rankings across the investigated bases.

## Graphical abstract

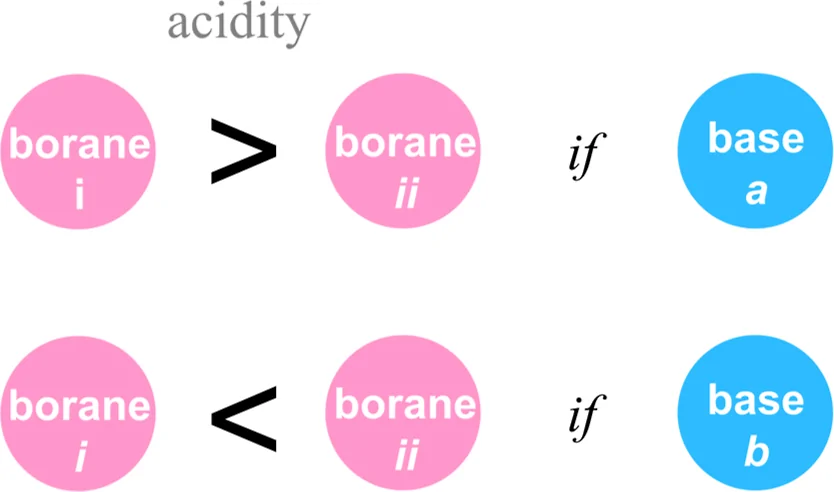

## 1 Introduction

Lewis acid–base interactions constitute a fundamental concept in chemistry, underpinning a broad spectrum of chemical reactivity and molecular behavior.^1^ Among Lewis acids, boron compounds occupy a central position because the three valence electrons of boron leave the atom electron-deficient and formally octet-incomplete, giving rise to pronounced Lewis acidity. Consequently, boron–Lewis base interactions have attracted sustained experimental and theoretical interest owing to their fundamental importance and broad applications in catalysis, materials science, and synthesis.^2–7^

A central challenge in boron chemistry is the reliable quantification of Lewis acidity. In 1982, Childs and co-workers developed an NMR-based Lewis acidity scale by examining the complexation of several Lewis acids with a series of α, β-unsaturated carbonyl and nitrile compounds.^8^ Importantly, the resulting acidity order was found to depend on the identity of the coordinating Lewis base, indicating that Lewis acidity cannot be regarded as a completely universal property. Subsequently, Beckett and co-workers introduced a convenient ^31^P NMR method for evaluating Lewis acidity using triethylphosphine oxide (TEPO) as a probe base.^9^ Although this approach has become one of the most widely used experimental measures of boron Lewis acidity, it relies on a single reference base and therefore cannot capture variations arising from changes in the interacting Lewis base.

The dependence of Lewis acid–base association on both interaction partners is established in qualitative and quantitative descriptions. In the Drago–Wayland approach, the enthalpy of adduct formation is correlated through the two-term expression −Δ*H* = *E*_*A*_ *E*_*B*_ + *C*_*A*_ *C*_*B*_, where each acid and base is characterized by electrostatic and covalent parameters.^10,11^ This framework allows the relative ordering of Lewis acids to depend on the interacting base. Accordingly, base-dependent acidity rankings are consistent with established thermochemical descriptions of Lewis acid–base association.

As boron chemistry continued to develop, increasing attention was directed toward understanding how structural and electronic factors influence the Lewis acidity. For example, Smith and co-workers demonstrated that substituent effects in 9-borafluorenes strongly influence their LUMO (lowest unoccupied molecular orbital) energies, electron affinities, air stability, and optical properties, providing valuable insights for the rational design of boron-containing conjugated materials.^12^ Lawson and Melen subsequently reviewed advances in borane chemistry and highlighted how substituent effects can be exploited to tune the Lewis acidity and reactivity of boranes, while emphasizing the central role of tris­(pentafluorophenyl)­borane, $B{\left(C_{6}F_{5}\right)}_{3}$ (BCF), together with the emergence of new boranes and borocations with broader applications.^13^

At the same time, a growing body of evidence has demonstrated that Lewis acidity is not determined solely by the Lewis acid but also by the identity of the interacting Lewis base. Greb and co-workers demonstrated that the behavior of frustrated Lewis pairs depends on the electronic properties of both the Lewis acid and the Lewis base rather than on the Lewis acid alone.^14^ Tussing, Greb, and co-workers subsequently showed that imines and amines exhibit comparable reactivity toward BCF but markedly different reactivities toward less Lewis acidic boranes.^15^ Similar conclusions were reached by Radius and co-workers, who showed that compounds displaying similar affinity toward NH_3_ may exhibit substantially different reactivities toward CO and CO_2_.^16,17^ More recently, Dutton and co-workers evaluated the Lewis acidity of carborane-substituted boranes using fluoride, hydride, and NH_3_ affinities together with the global electrophilicity index and LUMO energies and explicitly emphasized that no single universal descriptor can fully characterize Lewis acidity because the calculated values depend on the hardness or softness of the Lewis acid–base pair.^18^

Commonly used descriptors of Lewis acidity, such as fluoride ion affinity and hydride ion affinity, characterize interactions with specific reference bases. Erdmann and Greb computed multiple Lewis-base affinity scales for 183 p-block Lewis acids, demonstrating the importance of a multidimensional description of Lewis acidity.^19^ Mayer and co-workers established a quantitative Lewis acidity/basicity scale based on experimental equilibrium constants for borane–base association.^20^ These studies provide a quantitative foundation for examining how substituent variation affects borane–base association and the transferability of relative acidity rankings across different Lewis bases.

Complementary mechanistic insight was provided by Hamlin and co-workers, who investigated X_3_B–NY_3_ Lewis pairs (X, Y = F, Cl, Br, I, H) using quantum chemical calculations.^21^ Their analysis demonstrated that the strength of boron trihalide–ammonia adducts is strongly influenced by the energy required to deform the borane from its planar geometry to the pyramidal geometry adopted upon coordination, with weaker B–X bonds associated with lower deformation costs. Although this study provides detailed insight into borane–nitrogen donor interactions, it considered a restricted set of substituents and nitrogen-based Lewis bases.

The present study systematically examines substituent effects and base dependence in Lewis acidity within a consistently computed combinatorial library of 816 trisubstituted boranes constructed from 16 substituents. Interactions with five probe bases (H_2_O, NH_3_, pyridine, PMe_3_, and Cl^–^) are evaluated using a common solution-phase computational protocol. Density functional theory (DFT) calculations combined with machine learning (ML) analysis are employed to quantify adduct formation free energies, assess the transferability of relative borane acidity rankings across these bases, and identify electronic descriptors associated with the observed trends.

## 2 Results and Discussion

As illustrated in Scheme 1, the present study considers 16 representative substituents spanning a broad range of electron-withdrawing and electron-donating properties together with five representative Lewis bases (H_2_O, NH_3_, C_5_H_5_N, PMe_3_, and Cl^–^). Owing to the intrinsic symmetry of the borane framework, substitution at the three boron sites generates 816 unique trisubstituted boranes. Consequently, for each Lewis base, a total of 816 borane–base combinations^22–51^ were constructed and analyzed. All calculations were performed at the SMD/M06-2X/def2-TZVP//SMD/M06-2X/6-31G­(d) level of theory. Solvent effects were included using the SMD solvation model with dichloromethane as the solvent throughout.

**Scheme 1:**
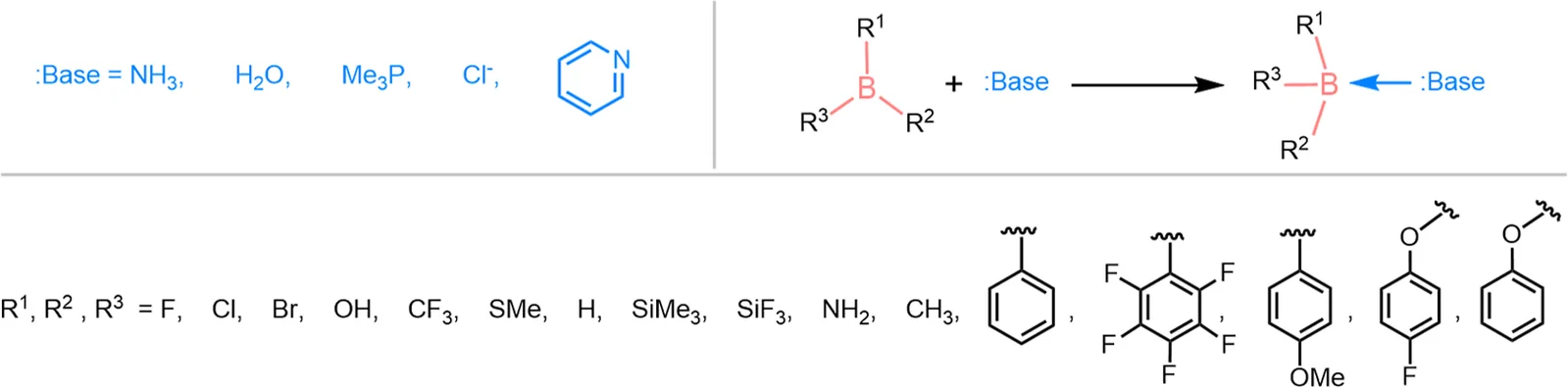
Representative R^1^, R^2^, and R^3^ Substituents Considered for Generating the Lewis Acidity Data Set

In certain cases, the substituents donate sufficient electron density to substantially attenuate the Lewis acidity of the borane, thereby precluding the formation of a stable Lewis acid–base adduct. In addition, hydrogen-bond formation is preferred over Lewis acid–base adduct formation for some borane–base combinations, and the corresponding hydrogen-bonding interaction energies are provided in the Supporting Information (Table S2). Steric effects also have the potential to inhibit adduct formation and give rise to frustrated Lewis pair behavior. However, based on our analysis, no evidence was found that steric hindrance was responsible for the excluded systems. Overall, 153 of the 4080 borane–base systems in the complete data set were excluded from further analysis, including 110 cases with H_2_O, 38 cases with Cl^–^, and 5 cases with PMe_3_ as the Lewis base.

Although the primary objective of the present work is to compare the relative Lewis acidity of substituted boranes toward different Lewis bases rather than to investigate their chemical reactivity, it is well documented that boron halides can undergo hydrolysis or ammonolysis upon reaction with H_2_O or NH_3_.^52,53^ To address this aspect, additional DFT calculations were performed to evaluate the thermodynamics of the corresponding substitution reactions. The calculated Gibbs free energies for substitution of halide substituents by hydroxyl or amino groups in a selected set of halogenated and methyl-substituted halogenated boranes are summarized in Tables S3–S8 of the Supporting Information.

Dimerization is a well-established characteristic of certain boranes; for example, B_2_H_6_ is significantly more stable than its monomeric counterpart BH_3_. Motivated by this behavior, the possibility of dimer formation was systematically examined for all of the systems considered. In cases where the dimeric species was found to be thermodynamically more stable than the corresponding monomeric borane, the dimer was adopted as the reference state for the energy calculations. Our analysis revealed that this behavior is predominantly observed for boranes bearing two hydrogen substituents (H_2_BR) in which hydrogen-bridged structures are formed. The corresponding DFT results are reported in Table S1 of the Supporting Information.

### 2.1 Dependence of Borane Lewis Acidity on the Interacting Lewis Base

In general, increasing the electron-donating character of the substituents on the boron center leads to a decrease in the borane Lewis acidity, resulting in weaker interactions with the corresponding Lewis base. A more detailed analysis of the relationships between substituent-derived features and borane Lewis acidity is presented in the ML section. The primary focus of the present study, however, is to elucidate the role of the Lewis base in governing the relative acidities of boranes. To facilitate a direct comparison, we first examine the relative Lewis acidity ordering of BR_3_ systems, in which all substituents on boron are identical upon interaction with different Lewis bases. The corresponding interaction energies were ranked according to adduct stability, from the most stable to the least stable species (Table 1). Systems not included in Table 1 correspond to cases where no stable Lewis adduct was identified based on our calculations.

**Table 1:** Base-Dependent Lewis Acidity Ordering of BR_3_ Systems, Ranked from the Most Stable to the Least Stable Lewis Adduct

NH_3_: B(CF_3_)_3_ > B(SiF_3_)_3_ > BBr_3_ > BCl_3_ > B(SiMe_3_)_3_ > B(C_6_F_5_)_3_ > BF_3_ > B_2_H_6_ > B(C_6_H_5_)_3_ > BMe_3_ > B(C_6_H_4_-4-OMe)_3_ > B(SMe)_3_ > B(OC_6_H_4_-4-F)_3_ > B(OC_6_H_5_)_3_ > B(OH)_3_ > B(NH_2_)_3_
pyridine: B(SiF_3_)_3_ > B(CF_3_)_3_ > BBr_3_ > BCl_3_ > B(SiMe_3_)_3_ > BF_3_ > B(C_6_F_5_)_3_ > B_2_H_6_ > B(C_6_H_5_)_3_ > BMe_3_ > B(OC_6_H_5_)_3_ > B(SMe)_3_ > B(OC_6_H_4_-4-F)_3_ > B(C_6_H_4_-4-OMe)_3_ > B(OH)_3_ > B(NH_2_)_3_
H_2_O: B(CF_3_)_3_ > B(SiF_3_)_3_ > BBr_3_ > B(C_6_F_5_)_3_ > BF_3_ > B(SiMe_3_)_3_ > BCl_3_ > B(C_6_H_5_)_3_ > BMe_3_ > B_2_H_6_ > B(C_6_H_4_-4-OMe)_3_ > B(OC_6_H_4_-4-F)_3_ > B(OC_6_H_5_)_3_ > B(SMe)_3_
PMe_3_: B(SiF_3_)_3_ > B(CF_3_)_3_ > B(SiMe_3_)_3_ > BBr_3_ > BCl_3_ > B(C_6_F_5_)_3_ > B_2_H_6_ > BF_3_ > B(C_6_H_5_)_3_ > BMe_3_ > B(C_6_H_4_-4-OMe)_3_ > B(SMe)_3_ > B(OC_6_H_4_-4-F)_3_ > B(OC_6_H_5_)_3_ > B(NH_2_)_3_
Cl^–^: B(CF_3_)_3_ > B(SiF_3_)_3_ > BBr_3_ > BCl_3_ > B(SiMe_3_)_3_ > B(C_6_F_5_)_3_ > BF_3_ > B_2_H_6_ > B(C_6_H_5_)_3_ > B(SMe)_3_ > B(C_6_H_4_-4-OMe)_3_ > BMe_3_ > B(OC_6_H_4_-4-F)_3_ > B(OC_6_H_5_)_3_

Based on the DFT results, the most stable borane–Lewis base adduct identified for each of the five studied bases (Table 1) corresponds to NH_3_· B­(CF_3_)_3_ (−61.4 kcal·mol^–1^), pyridine·B­(SiF_3_)_3_ (−53.1 kcal·mol^–1^), H_2_O· B­(CF_3_)_3_ (−35.5 kcal·mol^–1^), PMe_3_· B­(SiF_3_)_3_ (−71.4 kcal·mol^–1^), and Cl^–^·B­(CF_3_)_3_ (−64.3 kcal·mol^–1^). The relative Lewis acidity of boranes is, to some extent, dependent on the nature of the interacting Lewis base. For example, the calculated adduct formation free energies with NH_3_ are −61.4 and −55.8 kcal·mol^–1^ for $B{\left(CF_{3}\right)}_{3}$ and $B{\left(SiF_{3}\right)}_{3}$ , respectively, favoring $B{\left(CF_{3}\right)}_{3}$ by 5.6 kcal·mol^–1^. With PMe_3_, the ordering is reversed: the corresponding free energies are −60.2 and −71.4 kcal·mol^–1^, favoring $B{\left(SiF_{3}\right)}_{3}$ by 11.2 kcal·mol^–1^. Changing the probe base therefore shifts the relative preference between these two boranes by 16.8 kcal·mol^–1^. This reversal is consistent with established multidimensional descriptions of Lewis acidity^10,19^ and quantifies the sensitivity of the relative acidity of this borane pair to base identity under the present computational conditions.

As another illustrative example, BF_3_ behaves as a stronger Lewis acid than B_2_H_6_ toward NH_3_, pyridine, H_2_O, and Cl^–^. In contrast, this trend is reversed for Me_3_P, for which B_2_H_6_ exhibits greater Lewis acidity than BF_3_. Additional examples of such base-dependent inversions in relative Lewis acidity can be identified among the selected systems listed in Table 1 as well as throughout the complete data set.

Figure 1 illustrates the relationship between electrophilicity and the relative Gibbs free energy of borane–base adducts for boranes bearing identical substituents, while the corresponding correlation for the complete data set is provided in Figure S1 of the Supporting Information. These results show that the Lewis acidity decreases as the substituents on the borane become increasingly electron-donating, with a more pronounced effect observed for PMe_3_ compared to the other Lewis bases. For example, in interactions with boranes bearing strongly electron-withdrawing substituents, such as B­(SiF_3_)_3_, B­(SiMe_3_)_3_, and BBr_3_, PMe_3_ acts as the strongest Lewis base among the five Lewis bases investigated. In contrast, for boranes with electron-donating substituents, such as B­(SMe)_3_, B­(OC_6_H_4_-4-F)_3_, and B­(OC_6_H_5_)_3_, the corresponding PMe_3_ adducts (−4.9, 5.1, and 5.3 kcal·mol^–1^, respectively) are less stable than those formed with NH_3_ (−7.4, −5.4, and −4.6 kcal·mol^–1^) and Cl^–^ (−5.0, −2.8, and −1.6 kcal·mol^–1^). As a result, PMe_3_ exhibits the broadest range of adduct formation free energies across the data set, whereas H_2_O shows the narrowest, reflecting a stronger dependence on substituent electronic effects for PMe_3_. The pronounced dependence of PMe_3_ on the electronic nature of the borane substituents raises the question of whether this behavior originates primarily from methyl substitution or from replacement of nitrogen by phosphorus. To address this question, PH_3_ and NMe_3_ were included as additional reference Lewis bases.

**Figure 1:**
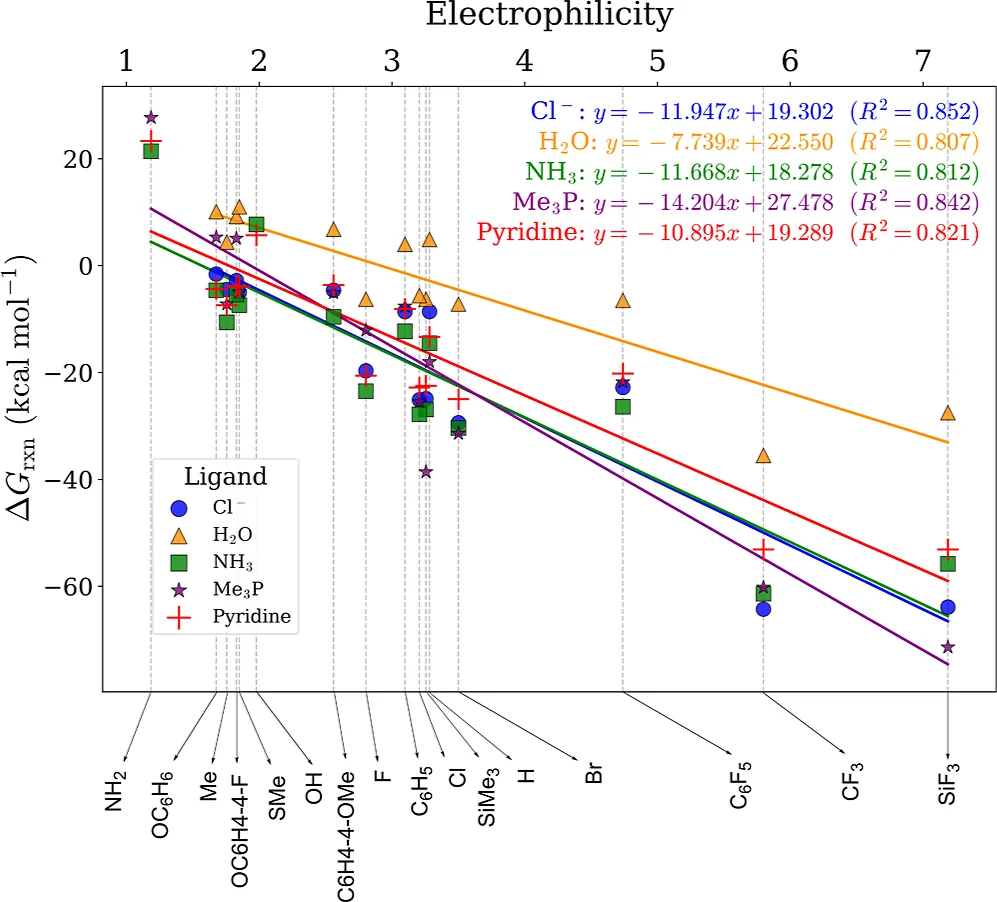
Scatter plots of the reaction energies versus borane electrophilicity for trisubstituted boranes (R^1^ = R^2^ = R^3^ = CF_3_, SiF_3_, Br, Cl, SiMe_3_, C_6_F_5_, F, H, C_6_H_5_, SMe, C_6_H_4_-4-OMe, Me, OC_6_H_4_-4-F, OC_6_H_5_, OH and NH_2_) interacting with different Lewis bases.

### 2.2 Structural Effects in Phosphine- and Amine–Borane Adduct Stability

PH_3_ and NMe_3_ were therefore examined as additional reference Lewis bases for the BR_3_ series (R^1^ = R^2^ = R^3^; Table 2). The results reveal several instructive trends. For phosphines, PH_3_ generally forms less stable adducts than PMe_3_. In contrast, the corresponding trend is less systematic for amines: in most cases, borane·NH_3_ adducts are more stable than borane·NMe_3_, although several exceptions are observed.

**Table 2:** Comparison of Nitrogen- and Phosphorus-Based Lewis Bases for Different Substituents R. All Δ*G*_rxn_ Values Correspond to the Formation of Base·BR_3_ Adducts and Are Reported in Kcal·mol^–1^ ^a^

Base:+BR_3_ → Base.BR_3_				
R	NH_3_	NMe_3_	PH_3_	PMe_3_
CF_3_	–61.4	–53.4	–40.7	–60.2
SiF_3_	–55.8	–57.1	–50.4	–71.4
Br	–30.4	–28.5	–10.2	–31.4
Cl	–27.8	–27.7	–5.1	–25.5
SiMe_3_	–26.9	–19.9	–28.1	–38.6
C_6_F_5_	–26.4	–4.2	–9.4	–21.8
F	–23.5	–25.5	3.6	–12.1
H	–14.5	–3.3	10.9	–18.0
C_6_H_5_	–12.3	1.8	3.0	–7.6
Me	–10.6	–8.1	4.7	–7.2
C_6_H_4_-4-OMe	–9.6	5.1	6.3	–5.1
SMe	–7.4	–4.1	8.7	–4.9
OC_6_H_4_-4-F	–5.4	–4.4	Dis	5.1
OC_6_H_5_	–4.6	1.8	Dis	5.3
OH	7.7	1.1	2.3	Dis
NH_2_	21.4	Dis	Dis	27.7

a Dis. denotes cases in which the Lewis base dissociated from the borane during the geometry optimization.

This difference can be rationalized by the greater steric congestion associated with tertiary amines than with tertiary phosphines. The shorter N–C bond lengths and the more compact coordination environment around nitrogen lead to greater crowding upon methyl substitution. Consequently, NMe_3_ experiences a larger steric penalty than Me_3_P in forming borane adducts, which can offset the electronic donation from methyl groups and render borane·NMe_3_ adducts less stable than the analogous phosphine adducts in many cases.

To further examine the effect of Lewis base size, additional calculations were carried out for the larger analogues Me_2_O and Br^–^, enabling direct comparison with H_2_O and Cl^–^, respectively. The corresponding results are presented in Table S9 of the Supporting Information.

### 2.3 Chemical Hardness as a Descriptor of Borane–Base Association

To examine the relationship between chemical hardness and adduct formation thermodynamics, we first considered selected boranes interacting with NH_3_ and PH_3_. Table 3 summarizes the calculated borane hardness values and the corresponding adduct formation free energies for the halide and chalcogen-containing series.

**Table 3:** Calculated Chemical Hardness Values of Selected Boranes and Reaction Free Energies for the Formation of Their NH_3_ and PH_3_ Adducts. Free Energies Are Reported in Kcal·mol^–1^ and Hardness Values in eV. The Calculated Hardness Values of NH_3_ and PH_3_ Are 5.65 and 5.33 eV, Respectively

Base + BR_3_ → Base·BR_3_			
R	hardness of borane (eV)	NH_3_	PH_3_
F	7.72	–23.5	3.6
Cl	4.86	–27.8	–5.1
Br	4.12	–30.4	–10.2
OH	6.04	7.7	2.3
SH	4.37	–8.2	9.7
SeH	3.04	–4.5	–1.3

As shown in Table 3, borane hardness decreases along the series BF_3_, BCl_3_, and BBr_3_, while adduct formation becomes more favorable for both NH_3_ and PH_3_. However, the trend differs between the two donors when comparing B­(SH)_3_ and B­(SeH)_3_: the softer B­(SeH)_3_ forms a less favorable adduct with PH_3_ than with NH_3_. These comparisons illustrate the dependence of the thermodynamic trends on the interacting donor. The greater stability of the boron trihalide adducts with NH_3_ than with PH_3_ should therefore not be interpreted solely in terms of hardness because donor strength and hardness are distinct properties.

To evaluate the usefulness of global hardness as a single linear descriptor, we examined its correlation with adduct formation free energy across the borane data set for each probe base. Figure 2 presents the results for NH_3_, while the corresponding plots for H_2_O, pyridine, PMe_3_, and Cl^–^ are provided in Figure S18 of the Supporting Information. The weak linear correlations indicate that the calculated global hardness of the isolated borane is insufficient as a single linear predictor of adduct formation free energy in this data set. This single-descriptor analysis does not constitute a test of the full HSAB framework or the Drago–Wayland thermochemical model.

**Figure 2:**
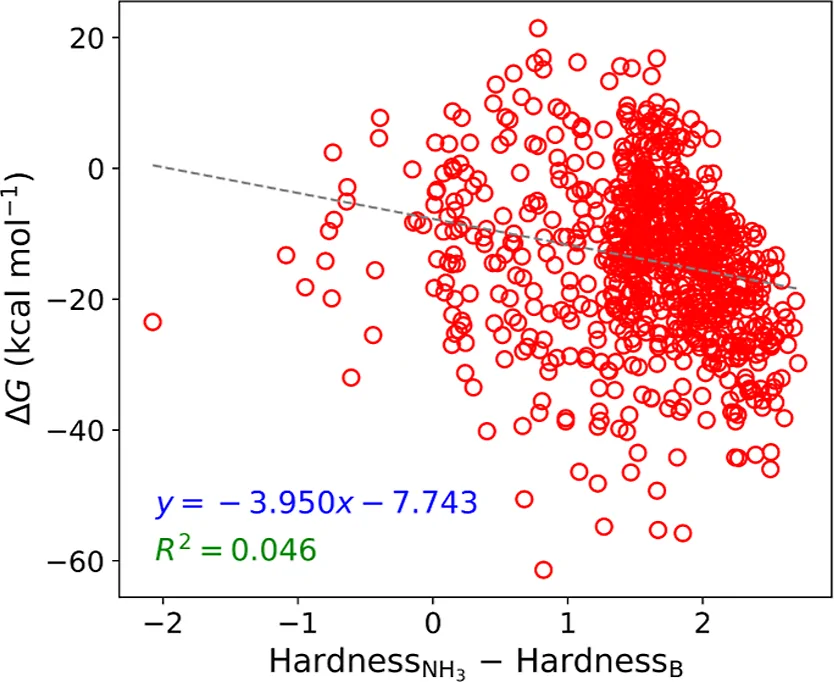
Linear correlation between the hardness difference (*η*_NH_3__-*η*_B_) and the calculated adduct formation free energy (Δ*G*) for boranes interacting with NH_3_. The regression equation and coefficient of determination (*R* ^2^) are shown in the plot.

### 2.4 Base-Dependent Lewis Acidity: Comparative Analysis Relative to NH_3_

In the following, the behavior of each Lewis base is analyzed relative to NH_3_, which is used as a reference Lewis base. Owing to the large number of borane systems investigated, representative examples are discussed below, whereas the complete set of results is provided in Figures S2–S17 of the Supporting Information. Figure 3 illustrates the variation in the relative adduct formation free energies for boranes with R^3^ = C_6_H_5_, while R^1^ and R^2^ span all substituents considered in this study.

**Figure 3:**
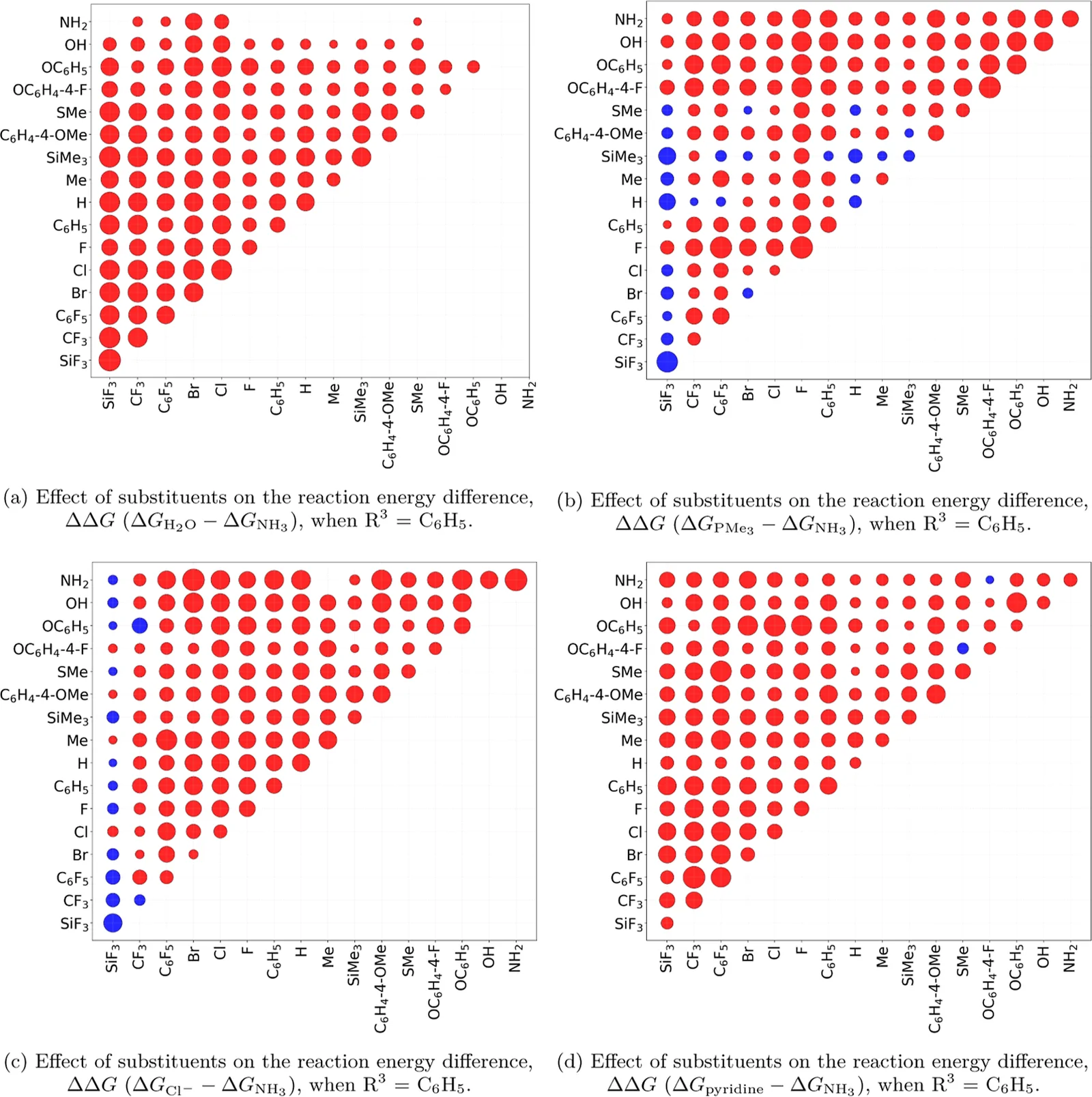
Effect of substituents on the adduct formation free energy difference, $\Delta \Delta G=\Delta G_{Base}-\Delta G_{NH_{3}}$ , for boranes with *R*_3_ = C_6_H_5_ and variable *R*_1_ and *R*_2_. The probe bases are (a) H_2_O, (b) PMe_3_, (c) Cl^–^, and (d) pyridine. Positive ΔΔ*G* values indicate more favorable adduct formation with NH_3_ (red circles), whereas negative values indicate more favorable adduct formation with the other probe base (blue circles). The diameter of each circle is proportional to the magnitude of ΔΔ*G*. The substituents are arranged from EWGs to EDGs.

Figure 3a compares the relative Lewis basicities of H_2_O and NH_3_. Across the series, NH_3_ consistently forms more stable adducts than H_2_O, although the magnitude of this preference depends on the borane substituent. Notably, the difference in adduct stability between NH_3_ and H_2_O increases as the substituents on the boron center become more electron withdrawing.

A more nuanced picture emerges for PMe_3_ (Figure 3b). Although PMe_3_ generally behaves as a stronger Lewis base than NH_3_ toward boranes bearing strongly electron-withdrawing substituents, this preference diminishes progressively as the substituents become more electron-donating. The effect is particularly pronounced for boranes containing SiF_3_ substituents and, to a lesser extent, for those bearing SiMe_3_ or H substituents. In contrast, when at least one EDG substituent is present on the borane center, such as NH_2_, OH, OC_6_H_4_-4-F, OC_6_H_5_, or F, NH_3_ consistently exhibits higher Lewis basicity than Me_3_P across all examined cases. A similar trend is observed when comparing the Lewis basicity of Cl^–^ with that of NH_3_ (Figure 3c).

Due to the greater 2p orbital contribution to the nitrogen lone pair in NH_3_ than in pyridine, ammonia is generally regarded as the stronger Lewis base. This difference in Lewis basicity becomes particularly pronounced in interactions with boranes bearing electron-withdrawing substituents. However, for certain boranes, the stabilities of the corresponding NH_3_ and pyridine Lewis acid–base adducts are remarkably similar (Figure 3d).

Overall, the results indicate that, for boranes featuring EWG substituents, Cl^–^ exhibits superior Lewis basicity relative to NH_3_. As the substituents on the boron center become progressively more electron-donating, however, this balance shifts in favor of NH_3_, which increasingly forms the more stable adducts. These results confirm that comparisons of borane Lewis acidity without explicit consideration of the interacting Lewis base can, in certain cases, be misleading, and that the relative Lewis acidity ordering of boranes depends on the identity of the interacting Lewis base.

### 2.5 Machine-Learning Approach

To gain quantitative insights into the factors governing borane Lewis acidity, a machine-learning (ML) approach was employed to analyze the relationship between borane substituents and the reaction energies for interactions with each of the five Lewis bases investigated. The ML framework was further used to examine how the identity of the Lewis base influences the observed Lewis acid–base trends across the borane series. The reaction energies corresponding to all five Lewis bases were selected as the target variables, yielding a total of 3927 data points. A set of molecular descriptors were evaluated to quantify the influence of borane substituents on the reaction energies. Comprehensive ML analyses for each individual Lewis base are presented in the Supporting Information.

The molecular descriptors employed in the ML–DFT analysis include: frontier orbital energies, including the HOMO energy of the Lewis base and the LUMO energy of the borane; global reactivity indices of the investigated boranes, including electrophilicity^54,55^ (a measure of a molecule’s propensity to accept electrons), chemical potential (reflecting the tendency to gain or lose electron density), and chemical hardness (describing resistance to changes in electron distribution); the Hammett σ_p_ parameter^56^ of the borane substituents, which serves as an empirical indicator of their electron-donating or electron-withdrawing character and has also been employed as a molecular descriptor in recent machine-learning studies;^57–63^ and the natural population analysis (NPA) charge at the boron center. Detailed definitions of all descriptors are provided in the Supporting Information (Table S10).

These descriptors served as the input features for training random forest (RF) regression models.^64^ Details of the evaluation and comparison of the machine-learning algorithms are provided in the Supporting Information. The initial RF models were constructed using the complete set of descriptors. Subsequent SHapley Additive exPlanations (SHAP) analysis indicated that several features showed weak associations with the target variable and adversely affected the predictive performance. As a result, these descriptors were excluded, and the models were retrained using 5 highly informative features, namely, the σ_*p*_ parameters, LUMO_borane_–HOMO_base_ gap, Electrophilicity_borane_, Hardness_borane_, and NPA_boron_.

The predictive performance of the optimized RF regression model was evaluated over 50 iterations, corresponding to the average of 50 independent train test splits, as shown in Figure 4. The final model consisted of 200 decision trees, with node splitting allowed for a minimum of two samples and terminal nodes permitted to contain at least one sample. The model performance was assessed using out-of-bag (OOB) error estimation, which provides an internal estimate of RF predictive performance. However, when OOB scores are used for feature selection or hyperparameter tuning, an independent evaluation is needed to assess the generalization of the selected model. To enhance model generalizability and mitigate overfitting, hyperparameters were optimized through 10-fold cross validation. The optimized model yielded *R* ^2^ values of 0.99 for the training set, 0.94 for the validation set, and 0.94 based on OOB evaluation, together with a mean absolute error (MAE) of 2.47 kcal·mol^–1^. Overall, these performance metrics demonstrate that the selected descriptors successfully capture the key patterns and underlying reactivity trends in the data set.

**Figure 4:**
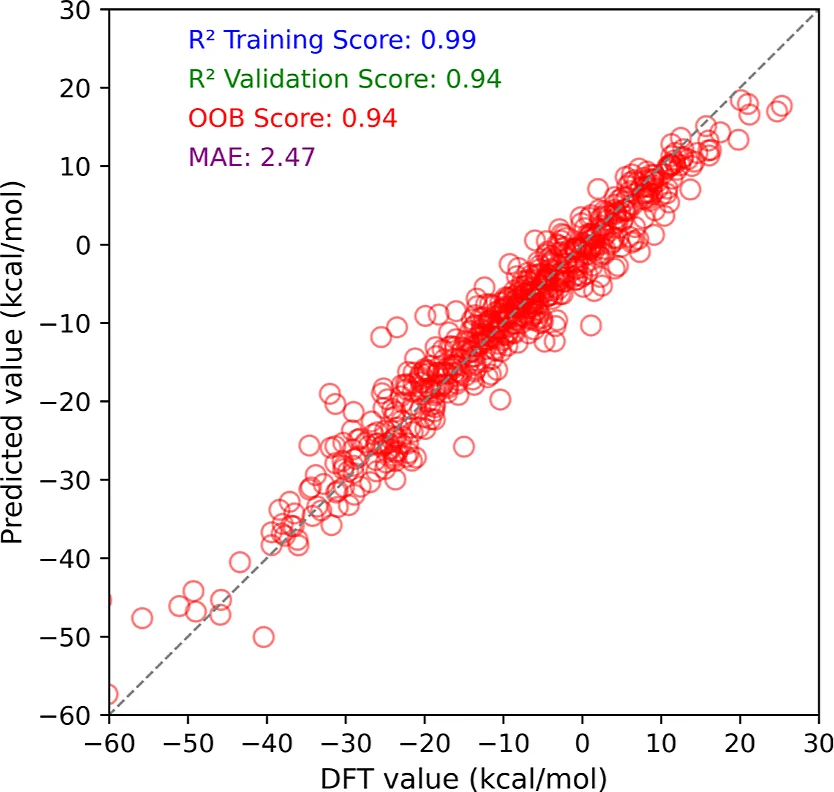
Scatter plots of DFT-computed versus RF-predicted Δ*G*_rxn_ values for the test subset (15% of the data set), using five input features.

### 2.6 Interpretation of Model Predictions Using SHAP

SHAP analysis was applied to interpret the RF model and quantify the relative importance of individual features.^65^ Each SHAP value represents the contribution of a descriptor to an individual prediction relative to the model’s baseline prediction. Negative and positive SHAP values indicate shifts toward more and less favorable predicted adduct formation free energies, respectively.

As shown in Figure 5, the summed Hammett σ_*p*_ parameter is the most influential descriptor in the model. Higher values, corresponding to a greater overall electron-withdrawing character of the borane substituents, are generally associated with negative SHAP values and more favorable predicted adduct formation free energies. Lower values, corresponding to a greater electron-donating character, generally show the opposite trend.

**Figure 5:**
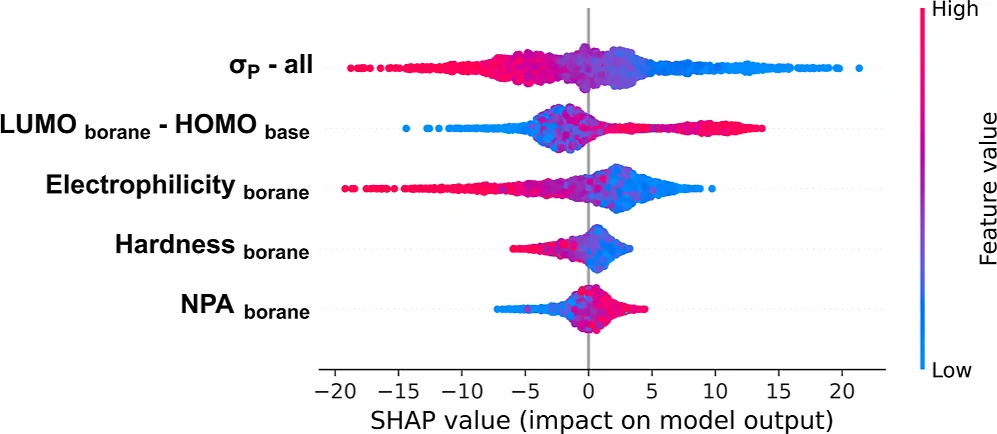
SHAP summary plot showing feature contributions to the predicted adduct formation free energies, expressed in kcal·mol^–1^. Each point represents an individual sample, and features are ranked by their mean absolute SHAP values. Colors indicate feature values, with blue representing low values and red representing high values.

The borane–base frontier orbital energy gap, defined as the borane LUMO energy minus the base HOMO energy, is the second most influential descriptor. Larger gaps are generally associated with less favorable predicted adduct formation free energies, whereas smaller gaps are associated with more favorable values. This trend is qualitatively consistent with a donor–acceptor description of Lewis acid–base interactions.

Borane electrophilicity is the third-most influential descriptor. Higher electrophilicity values generally correspond to negative SHAP values, whereas lower values tend to correspond to positive SHAP values. The borane chemical hardness and the NPA charge at the boron center make smaller contributions to the model predictions. Further details on correlations between descriptors are provided in Table S11 of the Supporting Information.

These results describe how the trained model uses the descriptors to predict the adduct formation free energies. They indicate statistical associations rather than independently establishing the physical origins of the observed trends.

### 2.7 Performance Evaluation of the Trained Model on the Studied Data Set

To assess the predictive capability of the trained ML model and evaluate its practical applicability, a separate set of 24 molecules was selected from the complete data set comprising approximately 3927 borane–base systems involving the five Lewis bases studied. These selected systems comprise 8 molecules bearing strongly electron-donating substituents and 16 molecules containing strongly electron-withdrawing substituents. After training the model on the full data set, the trained ML model was employed to predict ΔG_rxn_ for these selected systems. The predicted values were then compared with the corresponding DFT calculated energies, and the model performance was quantified in terms of the coefficient of determination (*R* ^2^) and MAE. As shown in Figure 6, the model achieves an *R* ^2^ value of approximately 0.98 and an MAE of 2.95 kcal·mol^–1^, indicating that the trained model is capable of accurately reproducing reaction energies across a diverse set of molecules.

**Figure 6:**
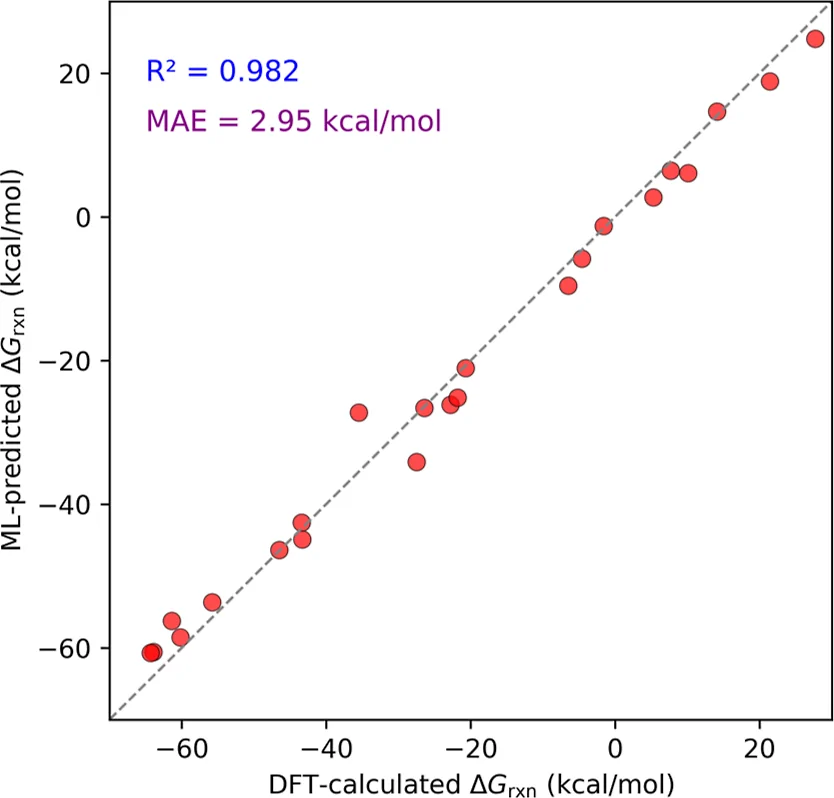
Scatter plots of DFT-computed Δ*G*_rxn_ versus predicted values from the RF regression models.

## 3 Conclusion

In this work, we investigated how the identity of the interacting Lewis base influences the Lewis acidity trends of the substituted boranes. A common solution-phase computational protocol yielded 3927 adduct formation free energies for a combinatorial library of 816 trisubstituted boranes constructed from 16 substituents and evaluated against five probe bases. These results quantify the magnitude and substituent dependence of the established base-dependent acidity trends within the investigated chemical space. The relative acidity ordering may change and, in some cases, even reverse, upon changing the Lewis base.

DFT calculations show that electron-withdrawing substituents generally enhance, whereas electron-donating substituents attenuate the Lewis acidity of boranes. However, the extent of these effects varies considerably among the different Lewis bases. In particular, PMe_3_ exhibits a substantially stronger dependence on the substituent identity than the other Lewis bases considered. Comparative analysis with PH_3_ and NMe_3_ further suggests that both donor atom identity and methyl substitution contribute to this behavior, although these comparisons do not independently separate their physical contributions.

The observed trends do not uniformly follow a simple hard–soft matching picture. The low coefficients of determination obtained for the linear correlations between isolated-borane global hardness and adduct formation free energy indicate that this descriptor alone has limited predictive power within the investigated data set. These correlations do not constitute a test of the full HSAB framework or the Drago–Wayland thermochemical model.

The machine-learning analysis provides a complementary statistical description of the calculated trends. SHAP analysis identifies the summed Hammett σ_*p*_ parameter of the borane substituents as the most influential descriptor in the model, followed by the borane–base frontier orbital energy gap and borane electrophilicity.

Overall, this study underscores that comparisons of Lewis acidity across borane series must be made with an explicit reference to the interacting Lewis base. Its contribution is a quantitative map of substituent effects and the transferability of relative borane acidity rankings under a common computational protocol. Extension to other solvents, donor classes, and experimentally accessible systems requires further validation.

## 4 Computational Details

All structures were fully optimized using Gaussian 16 ^66^ at the M06-2X level of theory.^67^ Solvent effects were incorporated via the SMD solvation model,^68^ employing dichloromethane as the solvent throughout. Geometry optimizations and subsequent frequency calculations were carried out using the 6–31G­(d) basis set.^69^ All reported optimized adduct structures were confirmed as local minima by the absence of imaginary vibrational frequencies. This basis set combination will be referred to as BS1. To refine the electronic energies obtained at the SMD/M06-2*X*/6-31G­(d) level, single-point energy calculations were performed using the def2-TZVP basis set.^70^ The def2-TZVP basis set (BS2) was employed for all atoms. A tight convergence criterion and an ultrafine integration grid were employed to enhance the accuracy of all calculations. The solution-phase Gibbs free energy for each species was computed according to the following expression

1 $$G=E\left(BS2\right)+G\left(BS1\right)-E\left(BS1\right)+\Delta G_{1atm\to 1M}$$

where *E*(BS1) and *E*(BS2) denote the electronic energies calculated at the respective levels of theory, and *G*(BS1) denotes the total Gibbs free energy obtained from the BS1 frequency calculation, including the electronic energy and thermal correction. Thus, *G*(BS1) – *E*(BS1) represents the thermal correction to the Gibbs free energy. The term Δ*G*_1 atm→1M_ = 1.89 kcal·mol^–1^ accounts for conversion from the 1 atm standard state to the 1 M standard state.

The reaction free energy for borane–base adduct formation was calculated according to the following expression

2 $$\Delta G_{rxn}=G_{Base\cdot BR_{3}}-\left(G_{Base}+G_{BR_{3}}\right)$$

where $G_{Base\cdot BR_{3}}$ is the Gibbs free energy of the borane–base adduct, and *G*_Base_ and $G_{BR_{3}}$ correspond to the Gibbs free energies of the isolated Lewis base and borane, respectively.

We employed RF regression (RFR) and gradient boosting ML models implemented in Python using the Scikit-learn package.^71^ These algorithms were selected for their ability to model nonlinear relationships between molecular descriptors and adduct formation free energies.

## Supplementary Material



## Data Availability

The data sets, including a table of the calculated adduct formation free energies for the studied borane–Lewis base combinations, and the source code used in this study are publicly available through the following GitHub repository: https://github.com/mahdianamir/Borane

## Notes

Additional calculated reaction energies, analyses of substituent effects, quantum chemical descriptors, machine-learning model comparisons, and SHAP analyses (PDF)
