# Supplementary material for: Quantifying Base-Dependent Lewis Acidity in Substituted Boranes through DFT and Machine Learning
Source: Inorg Chem. 2026 Oct 19;65(43):25557–66. doi: 10.1021/acs.inorgchem.6c04676 (PMC13733270; doi:10.1021/acs.inorgchem.6c04676)
Supplement: Supplementary file 1 [file ic6c04676_si_001.pdf]

Supporting Information for:

Quantifying Base-Dependent Lewis Acidity in Substituted Boranes through DFT and Machine Learning

Amir Mahdian[a], Kari Laasonen*[a], Kaveh Farshadfar*[a]

[a] *Department of Chemistry and Materials Science, School of Chemical Engineering, Aalto University, 02150 Espoo, Finland*

Contents

0.1 Comparison of reaction energies versus borane electrophilicity for five different Lewis bases. S3
0.2 Thermodynamics of Borane Dimerization S4
0.3 Formation of Intermolecular Hydrogen Bonds in Borane–Base Complexes S5
0.4 Calculated Reaction Energies for the Reactions of Substituted Boranes with H_2O S9
0.5 Calculated Reaction Energies for the Reactions of Substituted Boranes with NH_3 . . . . S12
0.6 Comparison of the Interaction Energies of Cl^-, Br^-, H_2O, and Me_2O across Different R Substituents S15
0.7 Substituent Effects on Relative Reaction Energies with H_2O and NH_3 S16
0.8 Substituent Effects on Relative Reaction Energies with Me_3P and NH_3 S20
0.9 Substituent Effects on Relative Reaction Energies with Cl^- and NH_3 S24

Email addresses: `kari.laasonen@aalto.fi` (Kari Laasonen*), `kaveh.farshadfar@aalto.fi` (Kaveh Farshadfar*)

0.10 Substituent Effects on Relative Reaction Energies with Pyridine and NH_3 . . . . S29
0.11 Linear Scaling Relationships between Hardness and Reaction Free Energy S33
0.12 Summary of Quantum Chemical Descriptors for Predictive Modeling S34
0.13 Assessment of Machine Learning Approaches in Predicting Reaction Energies S36
0.14 Scatter Plots and SHAP Analysis of DFT Reaction Energies versus Machine Learning Predictions for Different Lewis Bases S40

0.1. Comparison of reaction energies versus borane electrophilicity for five different Lewis bases.

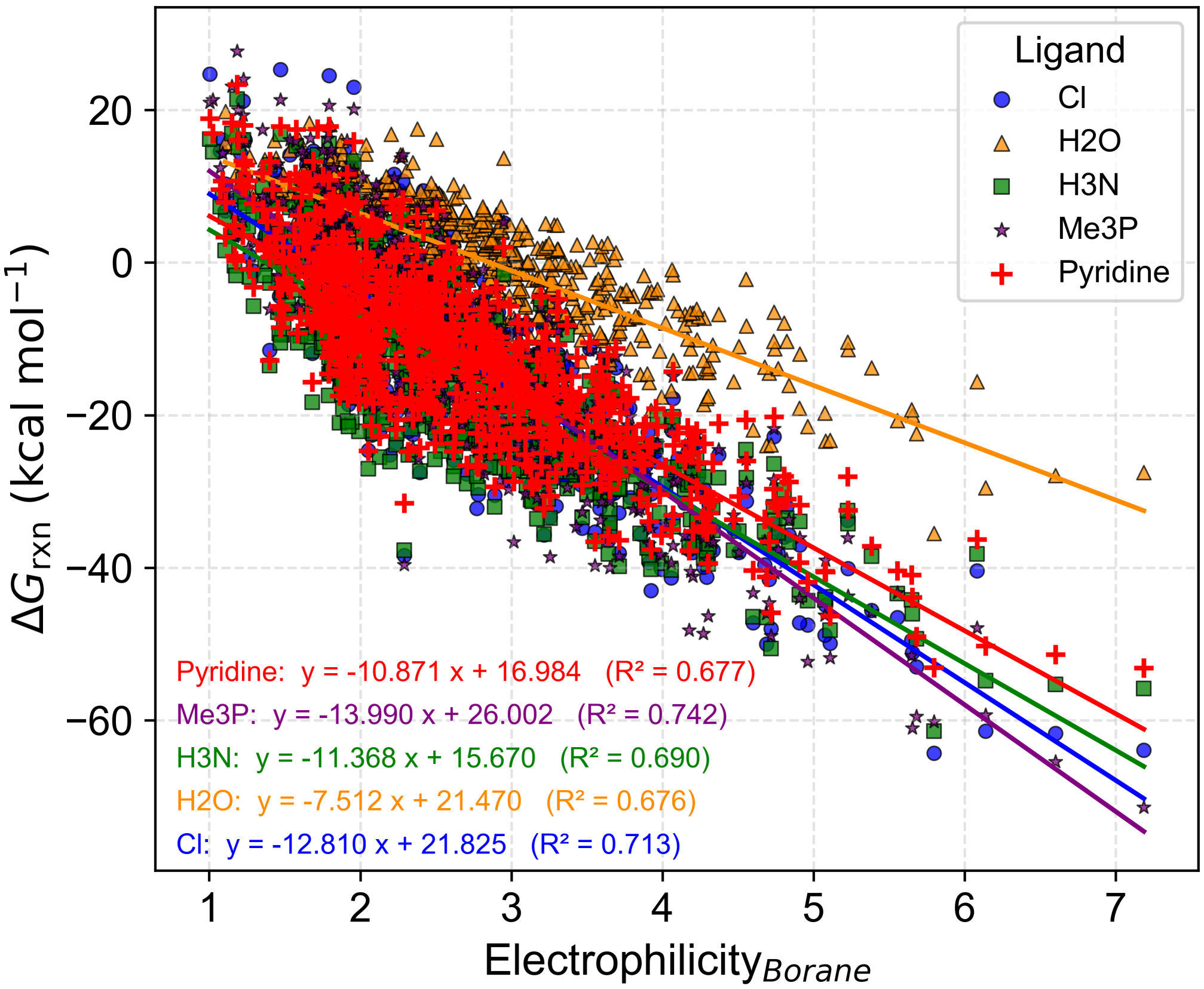

Figure S1: Scatter plots of reaction energies versus borane electrophilicity for R_3-substituted boranes interacting with various Lewis bases.

0.2. Thermodynamics of Borane Dimerization

Considering the possibility of dimerization in borane base complexes bearing specific substituents, dimer formation was evaluated for all relevant systems. The relative dimerization energies for the different substituents are summarized in Table S1. In cases where the dimeric form was found to be thermodynamically more favorable than the corresponding monomer, the dimer was considered as the resting state in all subsequent calculations.

Table S1: Calculated gibbs free energies of borane dimerization, defined as $\Delta G_{\text{dimer}} = G(A_2) - 2G(A)$, where A is the monomeric borane. Values are reported in kcal mol^{-1}.

**Complexes**	ΔG_{dimer} **(kcal/mol)**
$B_2H_4F_2$	-3.9
$B_2H_4Cl_2$	-10.6
$B_2H_4Br_2$	-12.1
$B_2H_4(CF_3)_2$	-27.0
$B_2H_4(C_6F_5)_2$	-15.3
$B_2H_4(C_6H_5)_2$	-15.6
$B_2H_4(C_6H_4\text{–4-OMe})_2$	-12.8
B_2H_6	-28.8
$B_2H_4(SiMe_3)_2$	-30.7
$B_2H_4Me_2$	-19.4

0.3. Formation of Intermolecular Hydrogen Bonds in Borane–Base Complexes

Table S2: Calculated interaction energies (ΔE) for borane–base complexes exhibiting hydrogen bonding.

**Complex**	ΔE **(kcal mol^{-1})**
$BF(OH)_2 \cdots Cl$	-17.7
$BH(NH_2)_2 \cdots Cl$	-5.9
$B(NH_2)_3 \cdots Cl$	-7.3
$BBr(NH_2)_2 \cdots Cl$	-18.5
$B(OH)(SiMe_3)(NH_2) \cdots Cl$	-11.9
$B(OH)(NH_2)_2 \cdots Cl$	-11.4
$B(OH)Me(NH_2) \cdots Cl$	-6.8
$B(C_6H_4\text{-4-OMe})(OH)_2 \cdots Cl$	-11.6
$BSMe(OH)_2 \cdots Cl$	-11.5
$B(NH_2)(OH)_2 \cdots Cl$	-8.6
$B(C_6F_5)(OH)_2 \cdots Cl$	-15.2
$B(OC_6H_5)(OH)_2 \cdots Cl$	-10.9
$B(OH)(OC_6H_4\text{-4-F})(NH_2) \cdots Cl$	-7.6
$B(OC_6H_4\text{-4-F})(NH_2)_2 \cdots Cl$	-8.5
$B(OC_6H_5)(NH_2)_2 \cdots Cl$	-8.3
$B(SiF_3)_2(NH_2) \cdots OH_2$	-11.0
$B(OC_6H_4\text{-4-F})(SiF_3)(OH) \cdots OH_2$	-9.3
$B(SiMe_3)(SiF_3)(NH_2) \cdots OH_2$	-8.5
$B(OC_6H_4\text{-4-F})(SiF_3)(NH_2) \cdots OH_2$	-8.1
$B(SiF_3)(OH)(NH_2) \cdots OH_2$	-7.9
$B(NH_2)_3 \cdots OH_2$	-7.6
$B(SiF_3)(OH)_2 \cdots OH_2$	-7.5
$B(SiF_3)F(NH_2) \cdots OH_2$	-7.2
$B(C_6H_4\text{-4-OMe})(SiF_3)(NH_2) \cdots OH_2$	-7.0
$B(SMe)(SiMe_3)(OH) \cdots OH_2$	-7.0
$B(SiF_3)Me(NH_2) \cdots OH_2$	-6.8
$B(OC_6H_5)(SiMe_3)OH \cdots OH_2$	-6.8
$B(OC_6H_5)_2(NH_2) \cdots OH_2$	-6.7
$B(OC_6H_5)(SiMe_3)(NH_2) \cdots OH_2$	-6.7

Continued on next page

Table S2: Calculated interaction energies (ΔE) for borane–base complexes exhibiting hydrogen bonding (continued).

**Complex**	ΔE **(kcal mol^{-1})**
$B(OH)(NH_2)_2 \cdots OH_2$	-6.6
$B(OC_6H_4-4-F)(SiMe_3)(NH_2) \cdots OH_2$	-6.6
$B(C_6F_5)(OH)_2 \cdots OH_2$	-6.5
$B(SMe)(NH_2)_2 \cdots OH_2$	-6.3
$B(C_6H_4-4-OMe)(NH_2)_2 \cdots OH_2$	-6.2
$B(SiF_3)(NH_2)_2 \cdots OH_2$	-6.2
$B(OC_6H_5)(NH_2)_2 \cdots OH_2$	-6.2
$B(OC_6H_4-4-F)(NH_2)_2 \cdots OH_2$	-6.2
$B(C_6H_5)(NH_2)_2 \cdots OH_2$	-6.1
$B(SiMe_3)(NH_2)_2 \cdots OH_2$	-6.1
$BMe(NH_2)_2 \cdots OH_2$	-6.1
$B(C_6H_4-4-OMe)(SiMe_3)(NH_2) \cdots OH_2$	-6.0
$B(OH)_3 \cdots OH_2$	-6.0
$B(OC_6H_4-4-F)(C_6H_4-4-OMe)(OH) \cdots OH_2$	-6.0
$B(C_6F_5)(NH_2)_2 \cdots OH_2$	-6.0
$B(SiMe_3)(OH)_2 \cdots OH_2$	-6.0
$B(SiMe_3)_2(NH_2) \cdots OH_2$	-5.9
$B(SMe)(SiMe_3)(NH_2) \cdots OH_2$	-5.9
$B(CF_3)(OC_6H_4-4-F)(NH_2) \cdots OH_2$	-5.9
$BH(OH)_2 \cdots OH_2$	-5.9
$B(CF_3)(OC_6H_5)(NH_2) \cdots OH_2$	-5.8
$B(C_6F_5)(SiF_3)(NH_2) \cdots OH_2$	-5.7
$B(CF_3)(SiMe_3)(NH_2) \cdots OH_2$	-5.6
$B(C_6H_5)(OC_6H_5)(NH_2) \cdots OH_2$	-5.6
$B(SiMe_3)F(OH) \cdots OH_2$	-5.6
$B(OC_6H_4-4-F)(OC_6H_5)(OH) \cdots OH_2$	-5.6
$B(OC_6H_4-4-F)_2(OH) \cdots OH_2$	-5.6
$B(OC_6H_5)Me(NH_2) \cdots OH_2$	-5.6
$B(C_6H_4-4-OMe)_2(NH_2) \cdots OH_2$	-5.6
$BH(NH_2)_2 \cdots OH_2$	-5.4
$B(SiMe_3)Me(OH) \cdots OH_2$	-5.4
$B(OC_6H_4-4-F)_2(NH_2) \cdots OH_2$	-5.4

Continued on next page

Table S2: Calculated interaction energies (ΔE) for borane–base complexes exhibiting hydrogen bonding (continued).

**Complex**	**ΔE (kcal mol^{-1})**
$B(C_6H_5)(C_6H_4-4-OMe)(NH_2)\cdots OH_2$	-5.4
$B(C_6F_5)(OC_6H_5)(NH_2)\cdots OH_2$	-5.4
$BMe(OH)(NH_2)\cdots OH_2$	-5.3
$B(OC_6H_5)(OH)(NH_2)\cdots OH_2$	-5.3
$B(SiMe_3)(OH)(NH_2)\cdots OH_2$	-5.3
$B(SiMe_3)Me(NH_2)\cdots OH_2$	-5.3
$BCl(NH_2)_2\cdots OH_2$	-5.3
$B(C_6F_5)(OC_6H_4-4-F)(NH_2)\cdots OH_2$	-5.3
$B(OC_6H_4-4-F)Me(NH_2)\cdots OH_2$	-5.2
$B(C_6H_5)(SiMe_3)(NH_2)\cdots OH_2$	-5.2
$B(C_6H_5)_2(NH_2)\cdots OH_2$	-5.2
$B(OC_6H_5)Me(OH)\cdots OH_2$	-5.1
$B(OC_6H_4-4-F)(OC_6H_5)(NH_2)\cdots OH_2$	-5.1
$B(OH)_2(NH_2)\cdots OH_2$	-5.1
$B(C_6H_4-4-OMe)Me(NH_2)\cdots OH_2$	-5.0
$B(OC_6H_5)(SMe)(NH_2)\cdots OH_2$	-5.0
$B(OC_6H_4-4-F)(SMe)(OH)\cdots OH_2$	-5.0
$BF(OC_6H_4-4-F)(OH)\cdots OH_2$	-5.0
$B(C_6F_5)(OH)(NH_2)\cdots OH_2$	-4.9
$BF(NH_2)_2\cdots OH_2$	-4.9
$BF(C_6F_5)(NH_2)\cdots OH_2$	-4.9
$B(C_6H_4-4-OMe)(OH)(NH_2)\cdots OH_2$	-4.9
$BCl(OC_6H_4-4-F)(NH_2)\cdots OH_2$	-4.9
$BBr(NH_2)_2\cdots OH_2$	-4.9
$B(C_6H_5)(OC_6H_5)(OH)\cdots OH_2$	-4.8
$B(SMe)(OH)(NH_2)\cdots OH_2$	-4.8
$B(OC_6H_4-4-F)(OH)(NH_2)\cdots OH_2$	-4.8
$B(C_6H_5)(OH)(NH_2)\cdots OH_2$	-4.8
$BMe(C_6H_5)(NH_2)\cdots OH_2$	-4.8
$B(CF_3)(OH)(NH_2)\cdots OH_2$	-4.8
$BH(SiMe_3)(NH_2)\cdots OH_2$	-4.8
$B(C_6H_5)(OC_6H_4-4-F)(OH)\cdots OH_2$	-4.7

Continued on next page

Table S2: Calculated interaction energies (ΔE) for borane–base complexes exhibiting hydrogen bonding (continued).

**Complex**	ΔE **(kcal mol^{-1})**
$B(C_6F_5)(SiMe_3)(NH_2)\cdots OH_2$	-4.7
$BF(OC_6H_4-4-F)(NH_2)\cdots OH_2$	-4.7
$B(OC_6H_5)(C_6H_4-4-OMe)(OH)\cdots OH_2$	-4.7
$BMe(C_6F_5)(NH_2)\cdots OH_2$	-4.7
$BF(SiMe_3)(NH_2)\cdots OH_2$	-4.5
$B(OC_6H_4-4-F)(OH)_2\cdots OH_2$	-4.5
$BBr(SiMe_3)(NH_2)\cdots OH_2$	-4.5
$B(OC_6H_5)(OH)_2\cdots OH_2$	-4.4
$BH(OC_6H_4-4-F)(NH_2)\cdots OH_2$	-4.4
$BFMe(NH_2)\cdots OH_2$	-4.4
$BHMe(NH_2)\cdots OH_2$	-4.4
$B(C_6H_4-4-OMe)(OH)_2\cdots OH_2$	-4.3
$B(C_6H_5)(OH)_2\cdots OH_2$	-4.2
$BF(C_6H_5)(NH_2)\cdots OH_2$	-4.2
$BH(C_6H_4-4-OMe)(NH_2)\cdots OH_2$	-4.1
$BF(C_6H_4-4-OMe)(NH_2)\cdots OH_2$	-4.1
$BH(C_6H_5)(NH_2)\cdots OH_2$	-4.1
$BH(C_6F_5)(NH_2)\cdots OH_2$	-4.1
$B(CF_3)(C_6H_4-4-OMe)(NH_2)\cdots OH_2$	-4.0
$BF(OH)(NH_2)\cdots OH_2$	-3.9
$BH_2(NH_2)\cdots OH_2$	-3.8
$B(OC_6H_5)(C_6H_4-4-OMe)(NH_2)\cdots OH_2$	-3.7
$BH(OH)(NH_2)\cdots OH_2$	-3.7
$B(OC_6H_4-4-F)(C_6H_4-4-OMe)(NH_2)\cdots OH_2$	-3.6
$B(C_6H_5)(OC_6H_4-4-F)(NH_2)\cdots OH_2$	-3.6
$B(SMe)(OH)_2\cdots OH_2$	-2.3

0.4. Calculated Reaction Energies for the Reactions of Substituted Boranes with H_2O

Table S3: Calculated reaction energies (ΔG) for the reactions of substituted boranes with three H_2O molecule.

Reaction	ΔG (kcal mol^{-1})
$BF_3 + 3H_2O \rightarrow B(OH)_3 + 3HF$	9.3
$BCl_3 + 3H_2O \rightarrow B(OH)_3 + 3HCl$	-35.6
$BBr_3 + 3H_2O \rightarrow B(OH)_3 + 3HBr$	-43.7
$BF_2Cl + 3H_2O \rightarrow B(OH)_3 + 2HF + HCl$	-6.6
$BF_2Br + 3H_2O \rightarrow B(OH)_3 + 2HF + HBr$	-9.8
$BFCl_2 + 3H_2O \rightarrow B(OH)_3 + HF + 2HCl$	-21.8
$BCl_2Br + 3H_2O \rightarrow B(OH)_3 + 2HCl + HBr$	-38.4
$BFBr_2 + 3H_2O \rightarrow B(OH)_3 + HF + 2HBr$	-27.9
$BClBr_2 + 3H_2O \rightarrow B(OH)_3 + HCl + 2HBr$	-41.1
$BFClBr + 3H_2O \rightarrow B(OH)_3 + HF + HCl + HBr$	-24.9
$BF_2Me + 3H_2O \rightarrow B(OH)_3 + 2HF + CH_4$	-17.9
$BFMe_2 + 3H_2O \rightarrow B(OH)_3 + HF + 2CH_4$	-50.4
$BCl_2Me + 3H_2O \rightarrow B(OH)_3 + 2HCl + CH_4$	-47.0
$BClMe_2 + 3H_2O \rightarrow B(OH)_3 + HCl + 2CH_4$	-63.0
$BBr_2Me + 3H_2O \rightarrow B(OH)_3 + 2HBr + CH_4$	-52.2
$BBrMe_2 + 3H_2O \rightarrow B(OH)_3 + HBr + 2CH_4$	-65.0

Table S4: Calculated reaction energies (ΔG) for the reactions of substituted boranes with two H_2O molecule.

Reaction	ΔG (kcal mol^{-1})
$BF_3 + 2H_2O \rightarrow BF(OH)_2 + 2HF$	4.4
$BCl_3 + 2H_2O \rightarrow BCl(OH)_2 + 2HCl$	-26.2
$BBr_3 + 2H_2O \rightarrow BBr(OH)_2 + 2HBr$	-31.8
$BF_2Cl + 2H_2O \rightarrow BF(OH)_2 + HF + HCl$	-11.5
$BF_2Br + 2H_2O \rightarrow BF(OH)_2 + HF + HBr$	-14.7
$BFCl_2 + 2H_2O \rightarrow BF(OH)_2 + 2HCl$	-26.7
$BCl_2Br + 2H_2O \rightarrow BCl(OH)_2 + HCl + HBr$	-29.0
$BFBr_2 + 2H_2O \rightarrow BF(OH)_2 + 2HBr$	-32.8
$BClBr_2 + 2H_2O \rightarrow BCl(OH)_2 + 2HBr$	-31.6
$BFClBr + 2H_2O \rightarrow BF(OH)_2 + HCl + HBr$	-29.8
$BF_2Me + 2H_2O \rightarrow BMe(OH)_2 + 2HF$	7.6
$BFMe_2 + 2H_2O \rightarrow BMe(OH)_2 + CH_4 + HF$	-24.8
$BCl_2Me + 2H_2O \rightarrow BMe(OH)_2 + 2HCl$	-21.5
$BClMe_2 + 2H_2O \rightarrow BMe(OH)_2 + HCl + CH_4$	-37.4
$BBr_2Me + 2H_2O \rightarrow BMe(OH)_2 + 2HBr$	-26.6
$BBrMe_2 + 2H_2O \rightarrow BMe(OH)_2 + HBr + CH_4$	-39.4

Table S5: Calculated reaction energies (ΔG) for the reactions of substituted boranes with one H_2O molecule.

Reaction	ΔG (kcal mol^{-1})
$BF_3 + H_2O \rightarrow BF_2OH + HF$	0.7
$BCl_3 + H_2O \rightarrow BCl_2OH + HCl$	-15.2
$BBr_3 + H_2O \rightarrow BBr_2OH + HBr$	-18.0
$BF_2Cl + H_2O \rightarrow BF_2OH + HCl$	-15.3
$BF_2Br + H_2O \rightarrow BF_2OH + HBr$	-18.5
$BFCl_2 + H_2O \rightarrow BFClOH + HCl$	-15.8
$BCl_2Br + H_2O \rightarrow BCl_2OH + HBr$	-18.0
$BFBr_2 + H_2O \rightarrow BFBrOH + HBr$	-18.4
$BClBr_2 + H_2O \rightarrow BClBrOH + HBr$	-17.9
$BFClBr + H_2O \rightarrow BFClOH + HBr$	-18.8
$BF_2Me + H_2O \rightarrow BFMeOH + HF$	2.3
$BFMe_2 + H_2O \rightarrow BMe_2OH + HF$	3.8
$BCl_2Me + H_2O \rightarrow BClMeOH + HCl$	-14.0
$BClMe_2 + H_2O \rightarrow BMe_2OH + HCl$	-8.8
$BBr_2Me + H_2O \rightarrow BBrMeOH + HBr$	-16.9
$BBrMe_2 + H_2O \rightarrow BMe_2OH + HBr$	-10.8

0.5. Calculated Reaction Energies for the Reactions of Substituted Boranes with NH_3

Table S6: Calculated reaction energies (ΔG) for the reactions of substituted boranes with three NH_3 molecule.

Reaction	ΔG (kcal mol^{-1})
$BF_3 + 3NH_3 \rightarrow B(NH_2)_3 + 3HF$	27.5
$BCl_3 + 3NH_3 \rightarrow B(NH_2)_3 + 3HCl$	-17.5
$BBr_3 + 3NH_3 \rightarrow B(NH_2)_3 + 3HBr$	-25.5
$BF_2Cl + 3NH_3 \rightarrow B(NH_2)_3 + 2HF + HCl$	11.5
$BF_2Br + 3NH_3 \rightarrow B(NH_2)_3 + 2HF + HBr$	8.3
$BFCl_2 + 3NH_3 \rightarrow B(NH_2)_3 + HF + 2HCl$	-3.7
$BCl_2Br + 3NH_3 \rightarrow B(NH_2)_3 + 2HCl + HBr$	-20.3
$BFBr_2 + 3NH_3 \rightarrow B(NH_2)_3 + HF + 2HBr$	-9.7
$BClBr_2 + 3NH_3 \rightarrow B(NH_2)_3 + HCl + 2HBr$	-23.0
$BFClBr + 3NH_3 \rightarrow B(NH_2)_3 + HF + HCl + HBr$	-6.7
$BF_2Me + 3NH_3 \rightarrow B(NH_2)_3 + 2HF + CH_4$	0.2
$BFMe_2 + 3NH_3 \rightarrow B(NH_2)_3 + HF + 2CH_4$	-32.3
$BCl_2Me + 3NH_3 \rightarrow B(NH_2)_3 + 2HCl + CH_4$	-28.9
$BClMe_2 + 3NH_3 \rightarrow B(NH_2)_3 + HCl + 2CH_4$	-44.9
$BBr_2Me + 3NH_3 \rightarrow B(NH_2)_3 + 2HBr + CH_4$	-34.1
$BBrMe_2 + 3NH_3 \rightarrow B(NH_2)_3 + HBr + 2CH_4$	-46.8

Table S7: Calculated reaction energies (ΔG) for the reactions of substituted boranes with two NH_3 molecule.

Reaction	ΔG (kcal mol^{-1})
$BF_3 + 2NH_3 \rightarrow BF(NH_2)_2 + 2HF$	11.9
$BCl_3 + 2NH_3 \rightarrow BCl(NH_2)_2 + 2HCl$	-21.5
$BBr_3 + 2NH_3 \rightarrow BBr(NH_2)_2 + 2HBr$	-28.0
$BF_2Cl + 2NH_3 \rightarrow BF(NH_2)_2 + HF + HCl$	-4.1
$BF_2Br + 2NH_3 \rightarrow BF(NH_2)_2 + HF + HBr$	-7.3
$BFCl_2 + 2NH_3 \rightarrow BF(NH_2)_2 + 2HCl$	-19.3
$BCl_2Br + 2NH_3 \rightarrow BCl(NH_2)_2 + HCl + HBr$	-24.3
$BFBr_2 + 2NH_3 \rightarrow BF(NH_2)_2 + 2HBr$	-25.3
$BClBr_2 + 2NH_3 \rightarrow BCl(NH_2)_2 + 2HBr$	-27.0
$BFClBr + 2NH_3 \rightarrow BF(NH_2)_2 + HCl + HBr$	-22.3
$BF_2Me + 2NH_3 \rightarrow BMe(NH_2)_2 + 2HF$	16.4
$BFMe_2 + 2NH_3 \rightarrow BMe(NH_2)_2 + CH_4 + HF$	-16.1
$BCl_2Me + 2NH_3 \rightarrow BMe(NH_2)_2 + 2HCl$	-12.7
$BClMe_2 + 2NH_3 \rightarrow BMe(NH_2)_2 + HCl + CH_4$	-28.7
$BBr_2Me + 2NH_3 \rightarrow BMe(NH_2)_2 + 2HBr$	-17.9
$BBrMe_2 + 2NH_3 \rightarrow BMe(NH_2)_2 + HBr + CH_4$	-30.7

Table S8: Calculated reaction energies (ΔG) for the reactions of substituted boranes with one NH_3 molecule.

Reaction	ΔG (kcal mol^{-1})
$BF_3 + NH_3 \rightarrow BF_2NH_2 + HF$	3.0
$BCl_3 + NH_3 \rightarrow BCl_2NH_2 + HCl$	-15.8
$BBr_3 + NH_3 \rightarrow BBr_2NH_2 + HBr$	-19.5
$BF_2Cl + NH_3 \rightarrow BF_2NH_2 + HCl$	-12.9
$BF_2Br + NH_3 \rightarrow BF_2NH_2 + HBr$	-16.1
$BFCl_2 + NH_3 \rightarrow BFClNH_2 + HCl$	-14.4
$BCl_2Br + NH_3 \rightarrow BCl_2NH_2 + HBr$	-18.6
$BFBr_2 + NH_3 \rightarrow BFBrNH_2 + HBr$	-18.1
$BClBr_2 + NH_3 \rightarrow BClBrNH_2 + HBr$	-19.0
$BFClBr + NH_3 \rightarrow BFClNH_2 + HBr$	-17.4
$BF_2Me + NH_3 \rightarrow BFMeNH_2 + HF$	6.0
$BFMe_2 + NH_3 \rightarrow BMe_2NH_2 + HF$	5.6
$BCl_2Me + NH_3 \rightarrow BClMeNH_2 + HCl$	-12.4
$BClMe_2 + NH_3 \rightarrow BMe_2NH_2 + HCl$	-7.0
$BBr_2Me + NH_3 \rightarrow BBrMeNH_2 + HBr$	-16.0
$BBrMe_2 + NH_3 \rightarrow BMe_2NH_2 + HBr$	-9.0

0.6. Comparison of the Interaction Energies of Cl^-, Br^-, H_2O, and Me_2O across Different R Substituents

For comparison, Br^- and Me_2O were additionally considered as analogues of Cl^- and H_2O, respectively. The results obtained for the formation of $Br^- \cdot BR_3$ and $Me_2O{\cdot}BR_3$ adducts ($R^1 = R^2 = R^3$) are summarized in Table S2 of the Supporting Information. These data indicate that, as expected, Br^- generally exhibits slightly weaker Lewis basicity than Cl^-. In addition, in most cases Me_2O displays stronger Lewis basicity than H_2O toward the corresponding boranes.

Table S9: Comparison of binding energies toward Cl^-, Br^-, H_2O, and Me_2O for different substituents R.

R	Cl^-	Br^-	H_2O	Me_2O
CF_3	-64.3	-54.8	-35.5	-37
SiF_3	-63.9	-58.9	-27.5	-34.2
Br	-29.4	-19.1	-7.2	-10.6
Cl	-25.1	-14.9	-5.6	-10.5
$SiMe_3$	-24.9	-19.7	-6.3	-4.7
C_6F_5	-22.8	-13.9	-6.5	-8
F	-19.7	-10.4	-6.3	-11.5
H	-8.6	12.1	4.9	13.7
C_6H_5	-8.6	-1.3	4	4.9
Me	-4.5	1.8	4.4	3.1
OC_6H_4 4 OMe	-4.6	3.5	6.8	7.6
SMe	-5	3.7	11	9.6
OC_6H_4 4 F	-2.8	5.8	9.2	5.5
OC_6H_5	-1.6	7.3	10.1	6.3
OH	H-bond	H-bond	H-bond	4
NH_2	H-bond	H-bond	H-bond	H-bond

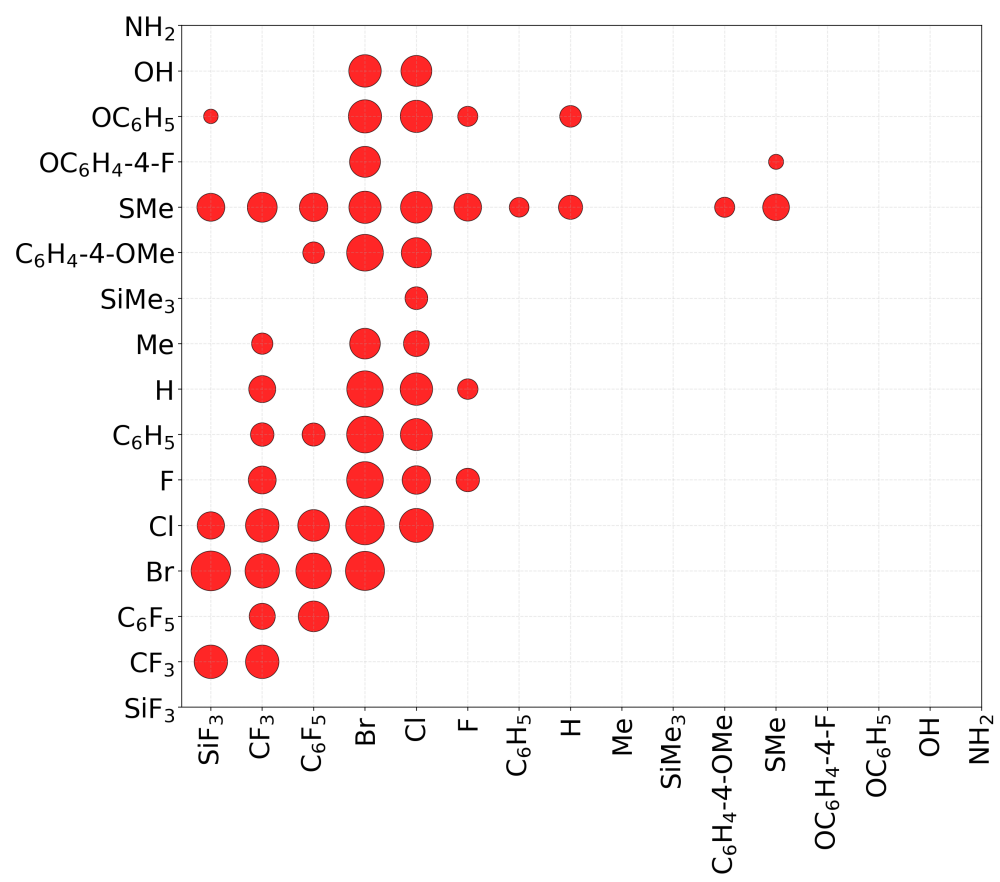

(a) Effect of substituents on the reaction energy difference, $\Delta\Delta G$ ($\Delta G_{\mathrm{H_2O}} - \Delta G_{\mathrm{NH_3}}$), when R^3 = NH_2.

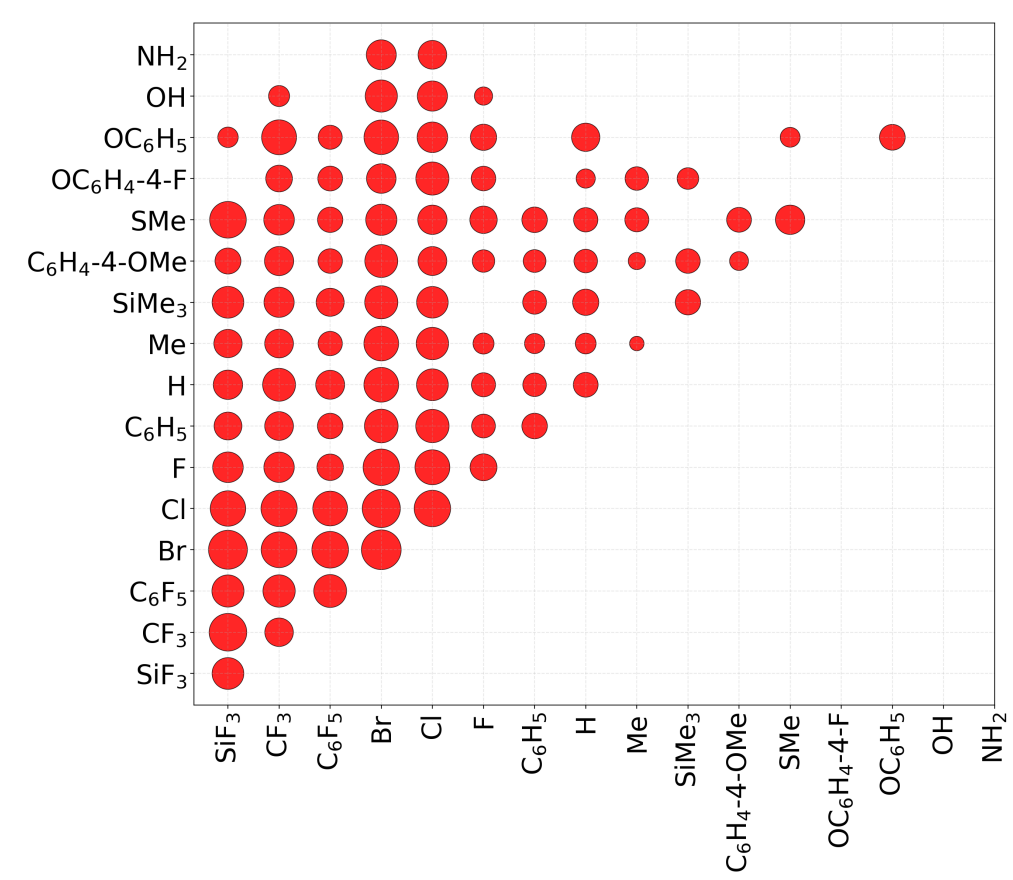

(b) Effect of substituents on the reaction energy difference, $\Delta\Delta G$ ($\Delta G_{\mathrm{H_2O}} - \Delta G_{\mathrm{NH_3}}$), when R^3 = OH.

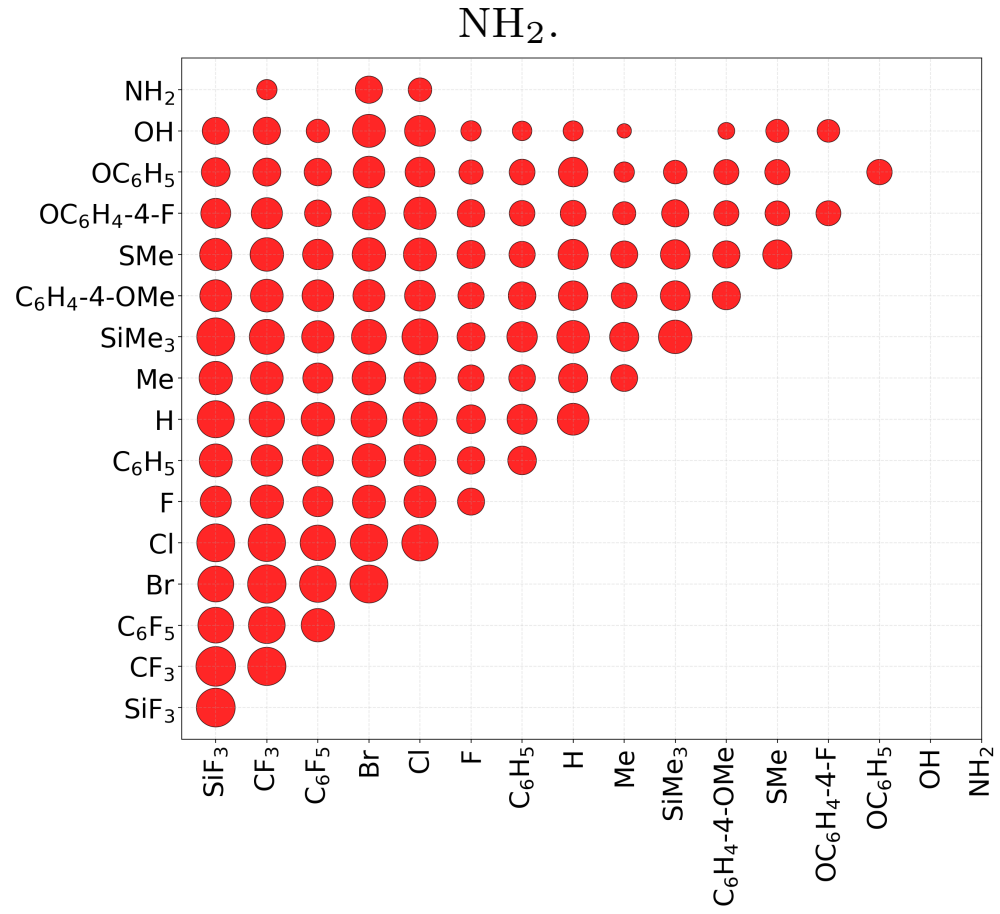

(c) Effect of substituents on the reaction energy difference, $\Delta\Delta G$ ($\Delta G_{\mathrm{H_2O}} - \Delta G_{\mathrm{NH_3}}$), when R^3 = Me.

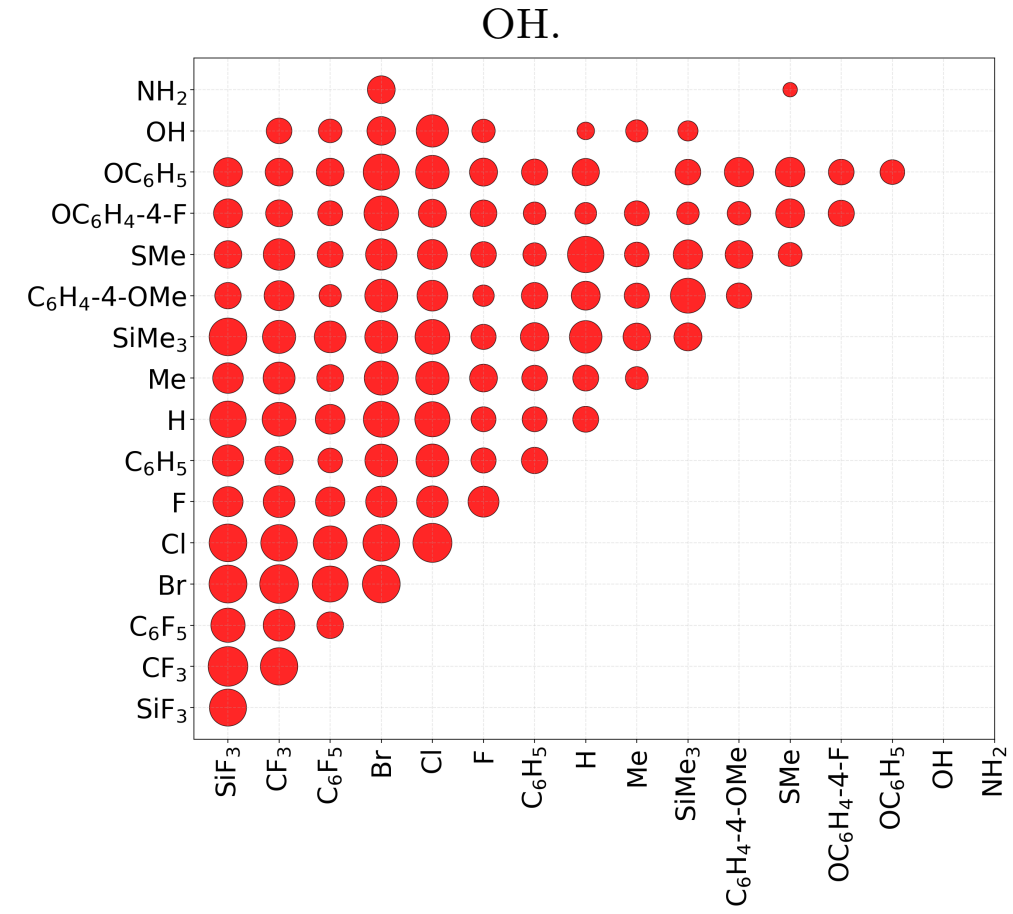

(d) Effect of substituents on the reaction energy difference, $\Delta\Delta G$ ($\Delta G_{\mathrm{H_2O}} - \Delta G_{\mathrm{NH_3}}$), when R^3 = OC_6H_4-4-F.

Figure S2: Effect of substituents on the reaction energy difference, $\Delta\Delta G$ ($\Delta G_{\mathrm{H_2O}} - \Delta G_{\mathrm{NH_3}}$). Positive $\Delta\Delta G$ values indicate a more favorable reaction with NH_3 as the Lewis base (represented by red circles), whereas negative $\Delta\Delta G$ values correspond to a greater stabilization with the H_2O (represented by blue circles). The diameter of each circle is proportional to the magnitude of $\Delta\Delta G$, with smaller circles indicating minor differences in reaction energies between the two bases.

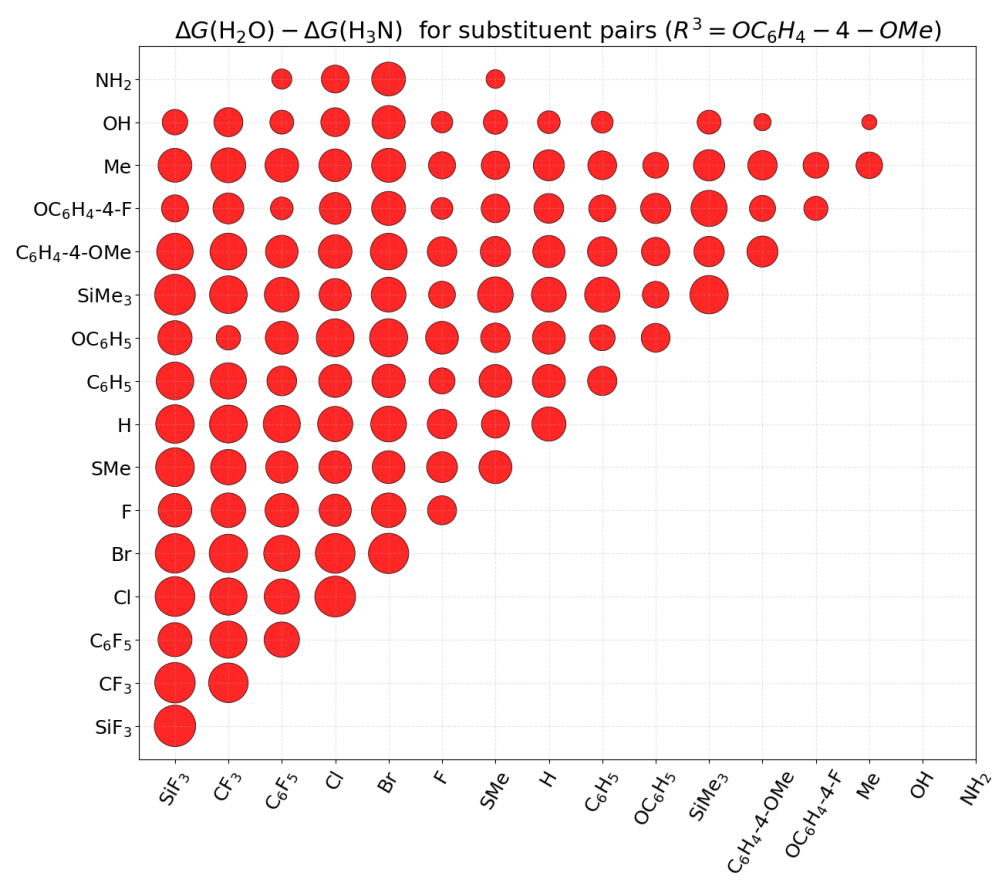

(a) Effect of substituents on the reaction energy difference, $\Delta\Delta G$ ($\Delta G_{H_2O} - \Delta G_{NH_3}$), when R^3 = C_6H_4-4-OMe.

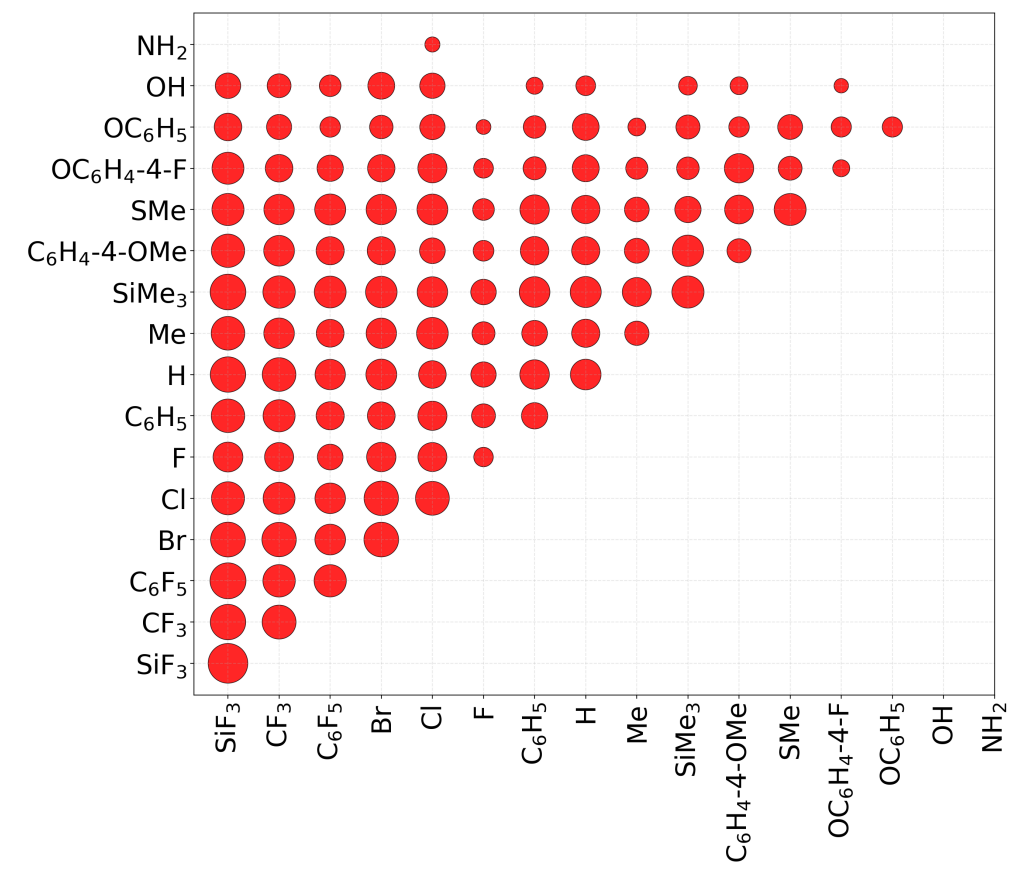

(b) Effect of substituents on the reaction energy difference, $\Delta\Delta G$ ($\Delta G_{H_2O} - \Delta G_{NH_3}$), when R^3 = $SiMe_3$.

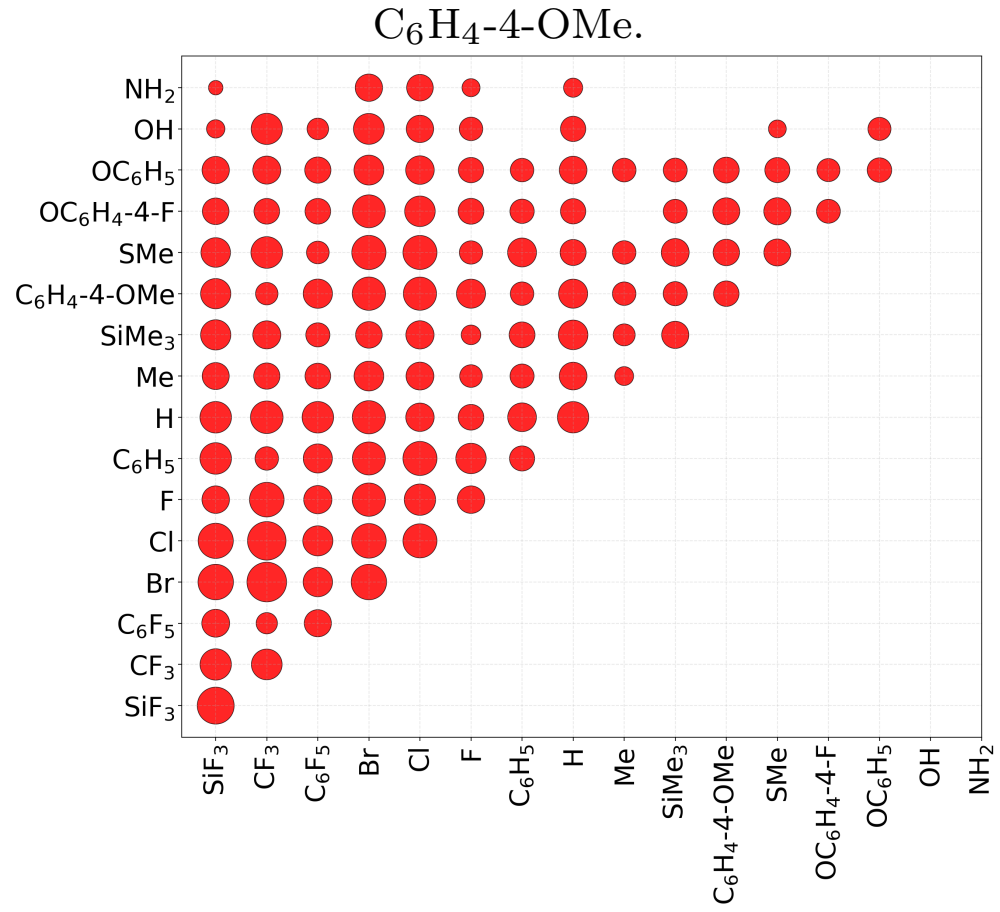

(c) Effect of substituents on the reaction energy difference, $\Delta\Delta G$ ($\Delta G_{H_2O} - \Delta G_{NH_3}$), when R^3 = OC_6H_5.

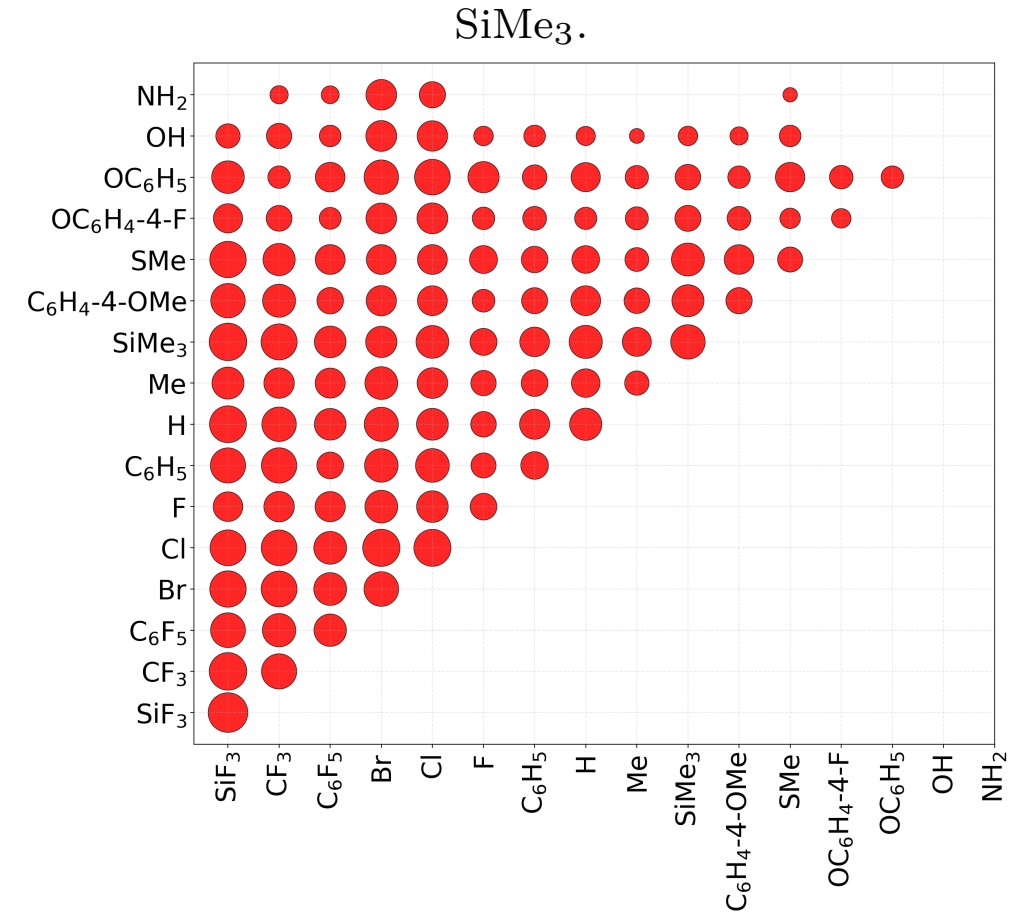

(d) Effect of substituents on the reaction energy difference, $\Delta\Delta G$ ($\Delta G_{H_2O} - \Delta G_{NH_3}$), when R^3 = C_6H_5.

Figure S3: Effect of substituents on the reaction energy difference, $\Delta\Delta G$ ($\Delta G_{H_2O} - \Delta G_{NH_3}$). Positive $\Delta\Delta G$ values indicate a more favorable reaction with NH_3 as the Lewis base (represented by red circles), whereas negative $\Delta\Delta G$ values correspond to a greater stabilization with the H_2O (represented by blue circles). The diameter of each circle is proportional to the magnitude of $\Delta\Delta G$, with smaller circles indicating minor differences in reaction energies between the two bases.

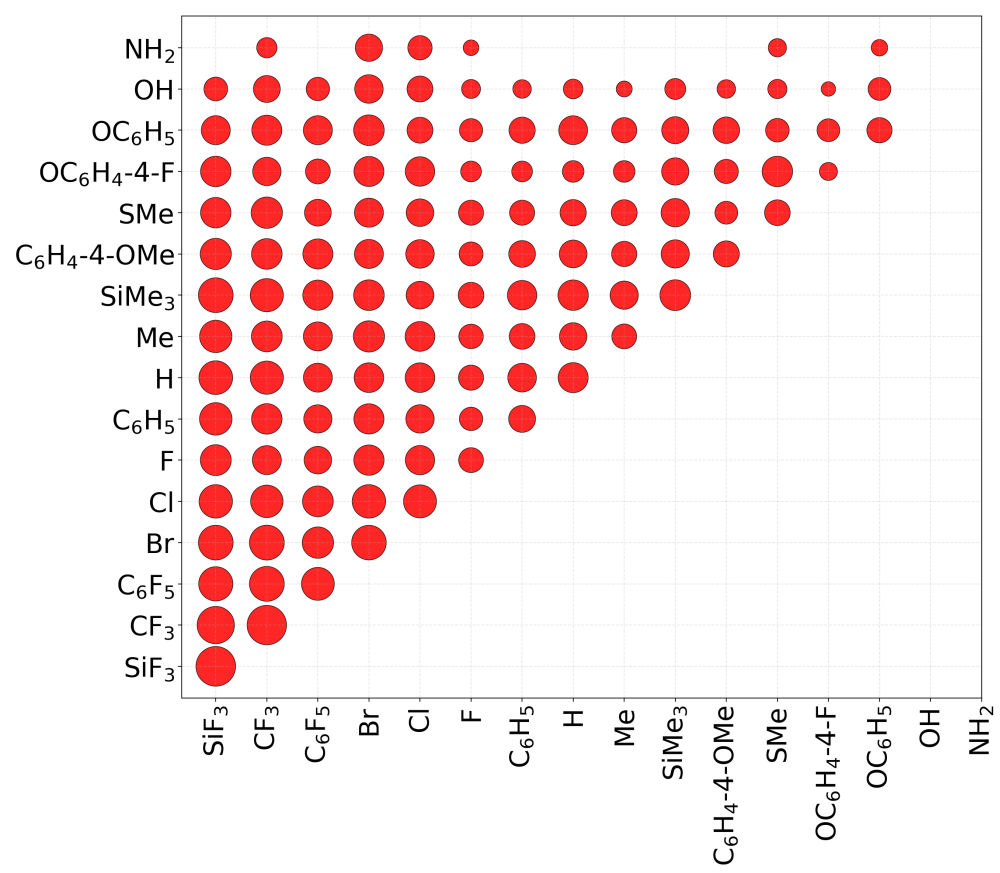

(a) Effect of substituents on the reaction energy difference, $\Delta\Delta G$ ($\Delta G_{\mathrm{H_2O}} - \Delta G_{\mathrm{NH_3}}$), when R^3 = H.

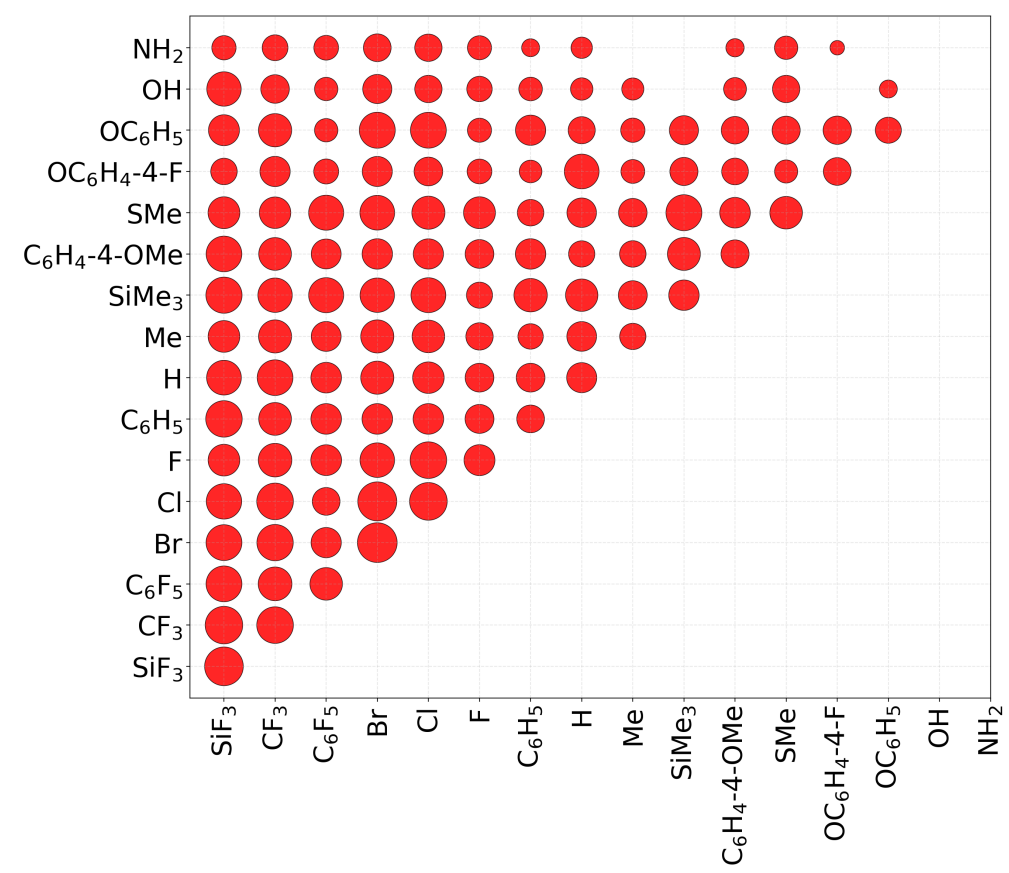

(b) Effect of substituents on the reaction energy difference, $\Delta\Delta G$ ($\Delta G_{\mathrm{H_2O}} - \Delta G_{\mathrm{NH_3}}$), when R^3 = SMe.

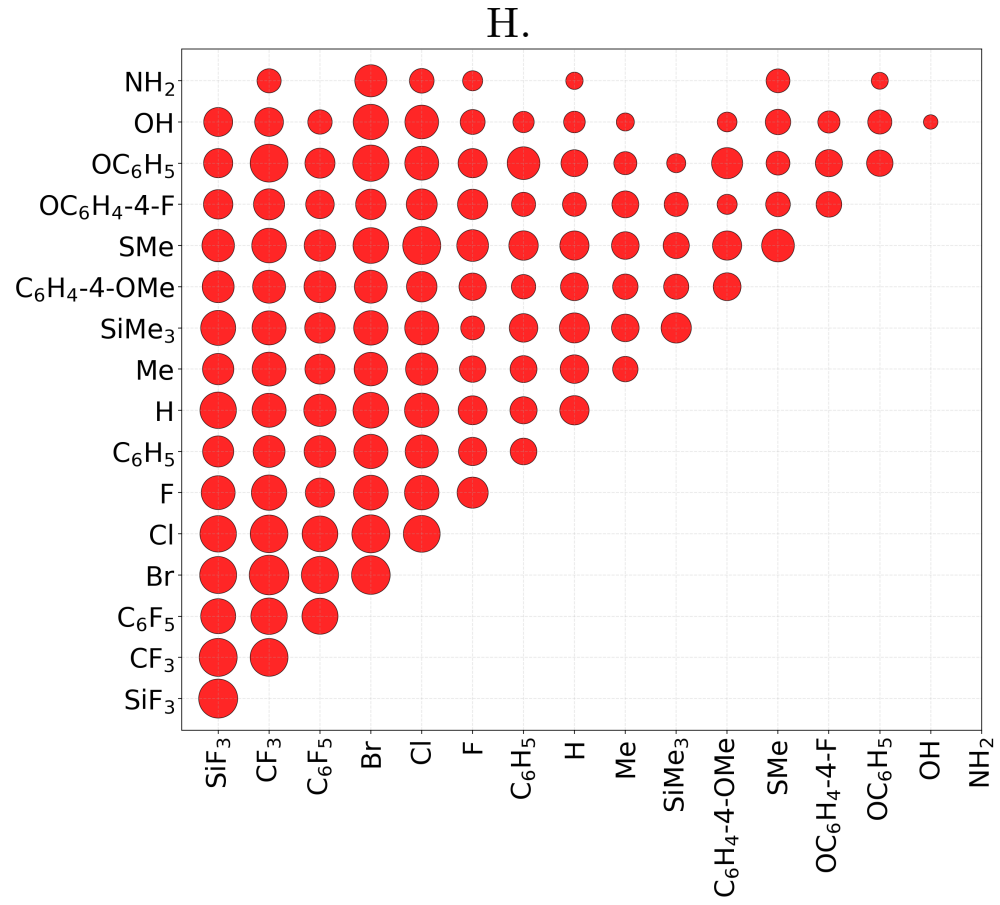

(c) Effect of substituents on the reaction energy difference, $\Delta\Delta G$ ($\Delta G_{\mathrm{H_2O}} - \Delta G_{\mathrm{NH_3}}$), when R^3 = F.

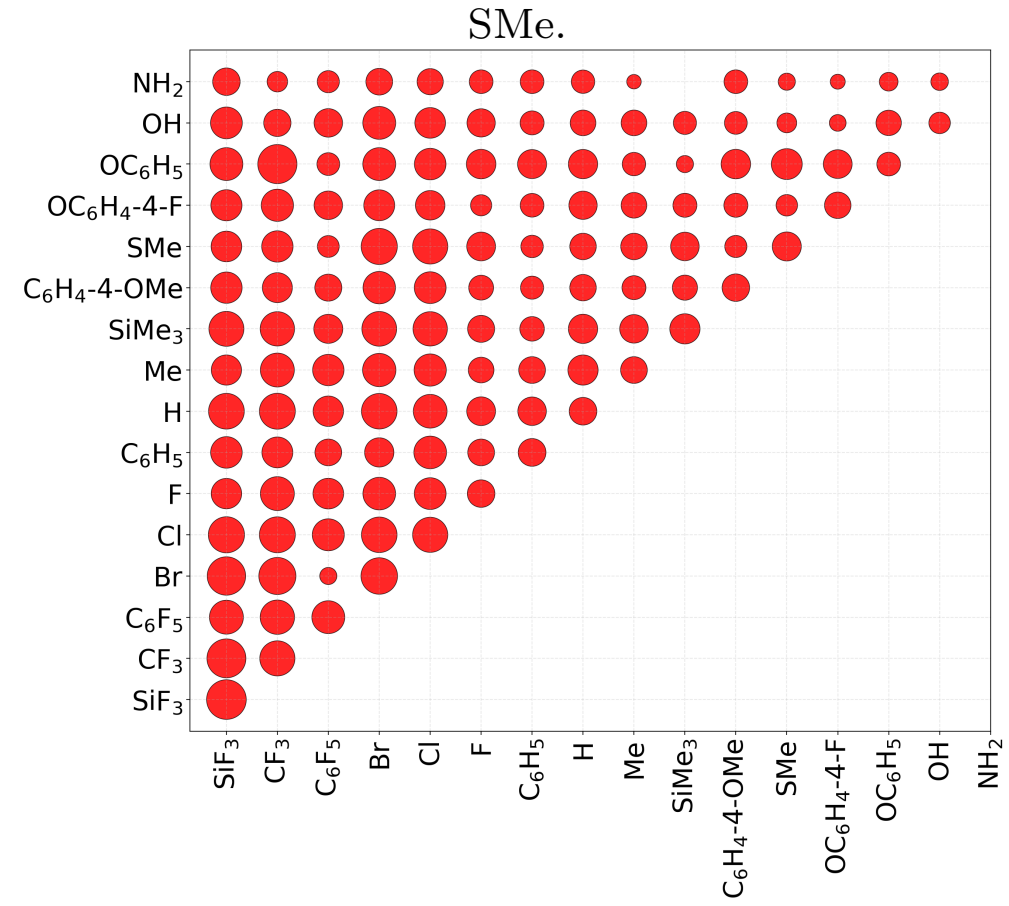

(d) Effect of substituents on the reaction energy difference, $\Delta\Delta G$ ($\Delta G_{\mathrm{H_2O}} - \Delta G_{\mathrm{NH_3}}$), when R^3 = Br.

Figure S4: Effect of substituents on the reaction energy difference, $\Delta\Delta G$ ($\Delta G_{\mathrm{H_2O}} - \Delta G_{\mathrm{NH_3}}$). Positive $\Delta\Delta G$ values indicate a more favorable reaction with NH_3 as the Lewis base (represented by red circles), whereas negative $\Delta\Delta G$ values correspond to a greater stabilization with the H_2O (represented by blue circles). The diameter of each circle is proportional to the magnitude of $\Delta\Delta G$, with smaller circles indicating minor differences in reaction energies between the two bases.

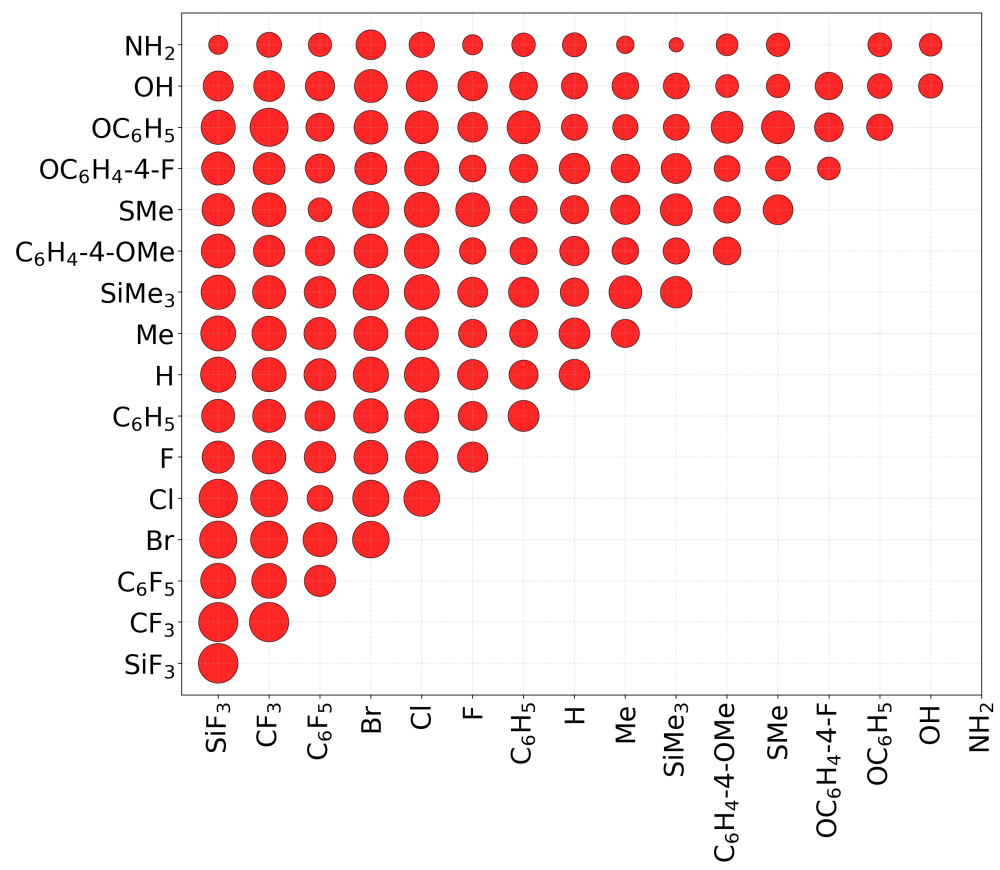

(a) Effect of substituents on the reaction energy difference, $\Delta\Delta G$ ($\Delta G_{\mathrm{H_2O}} - \Delta G_{\mathrm{NH_3}}$), when R^3 = Cl.

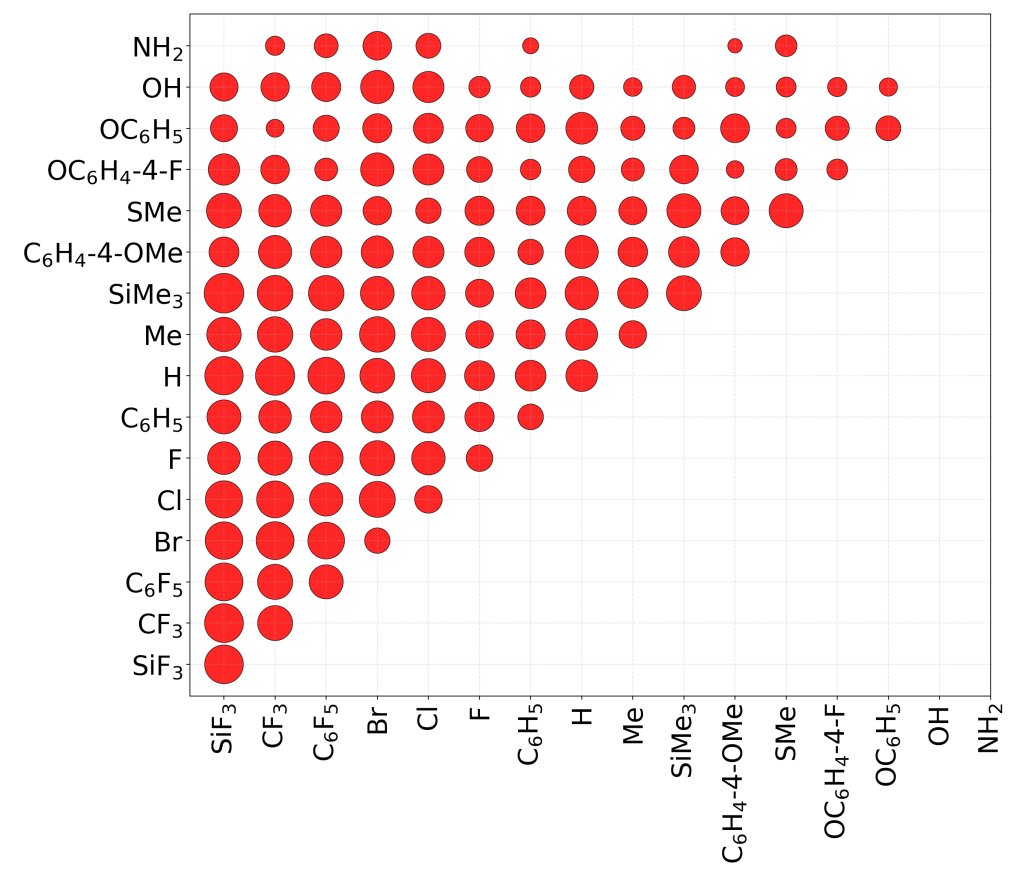

(b) Effect of substituents on the reaction energy difference, $\Delta\Delta G$ ($\Delta G_{\mathrm{H_2O}} - \Delta G_{\mathrm{NH_3}}$), when R^3 = C_6F_5.

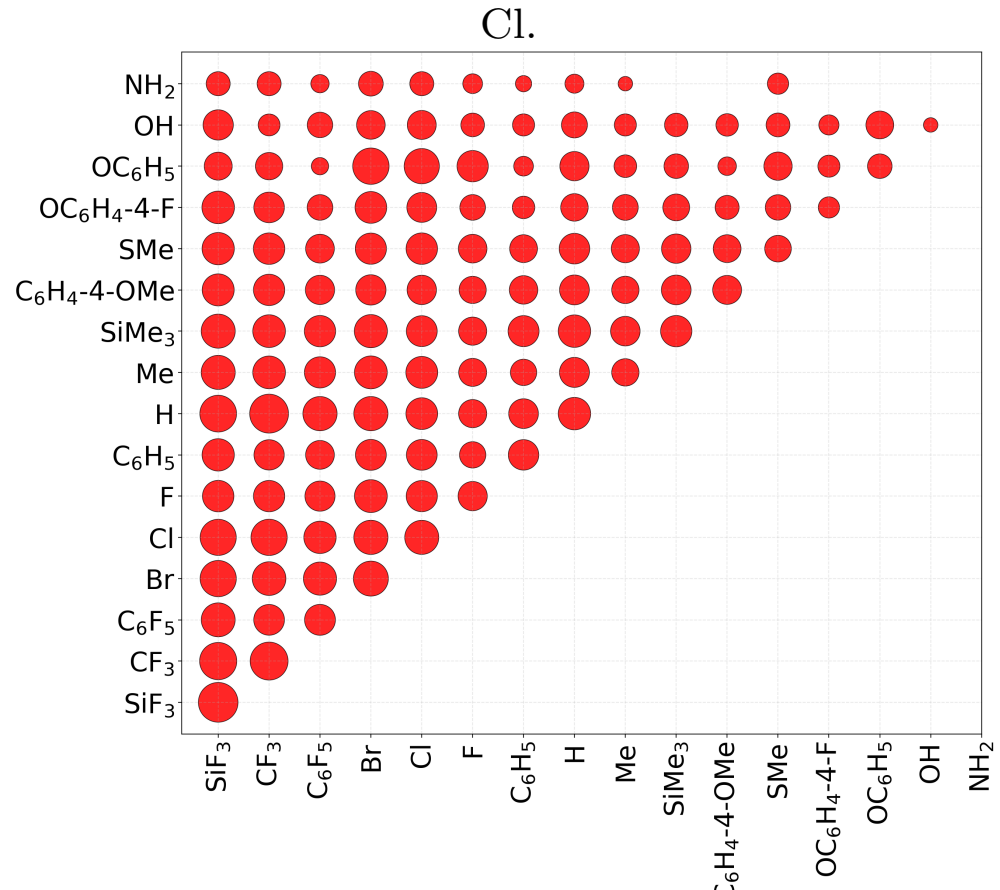

(c) Effect of substituents on the reaction energy difference, $\Delta\Delta G$ ($\Delta G_{\mathrm{H_2O}} - \Delta G_{\mathrm{NH_3}}$), when R^3 = CF_3.

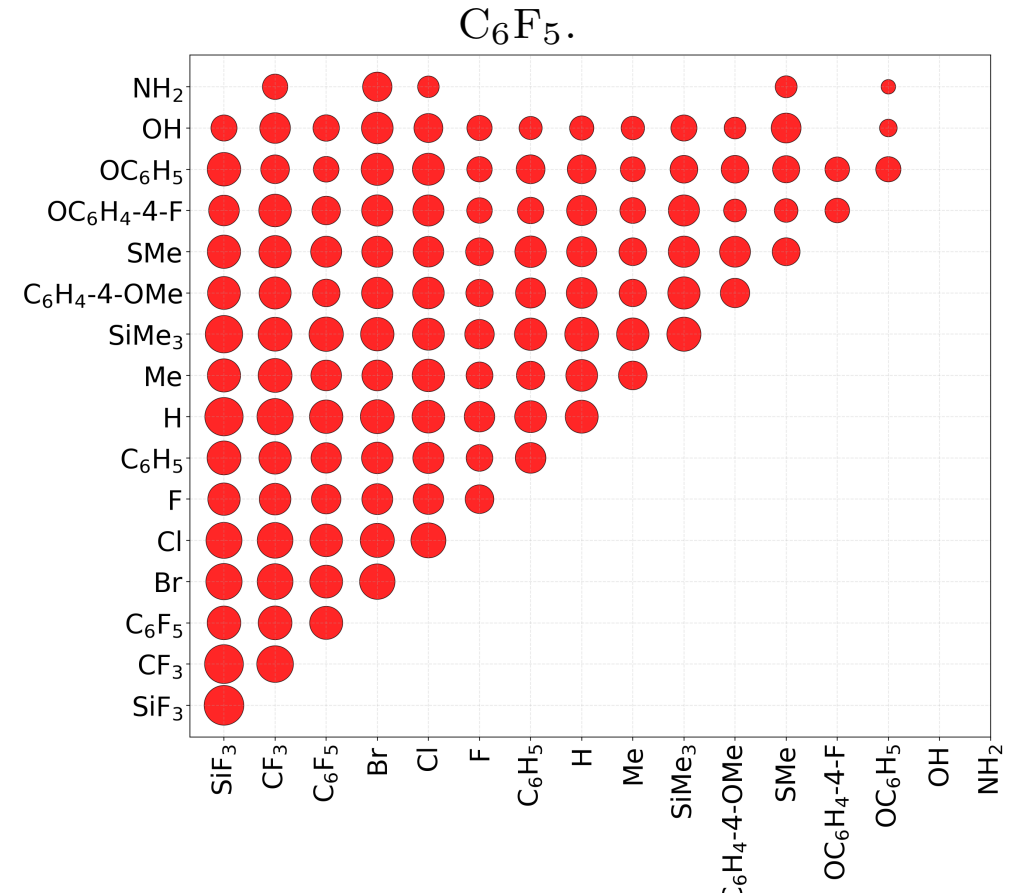

(d) Effect of substituents on the reaction energy difference, $\Delta\Delta G$ ($\Delta G_{\mathrm{H_2O}} - \Delta G_{\mathrm{NH_3}}$), when R^3 = SiF_3.

Figure S5: Effect of substituents on the reaction energy difference, $\Delta\Delta G$ ($\Delta G_{\mathrm{H_2O}} - \Delta G_{\mathrm{NH_3}}$). Positive $\Delta\Delta G$ values indicate a more favorable reaction with NH_3 as the Lewis base (represented by red circles), whereas negative $\Delta\Delta G$ values correspond to a greater stabilization with the H_2O (represented by blue circles). The diameter of each circle is proportional to the magnitude of $\Delta\Delta G$, with smaller circles indicating minor differences in reaction energies between the two bases.

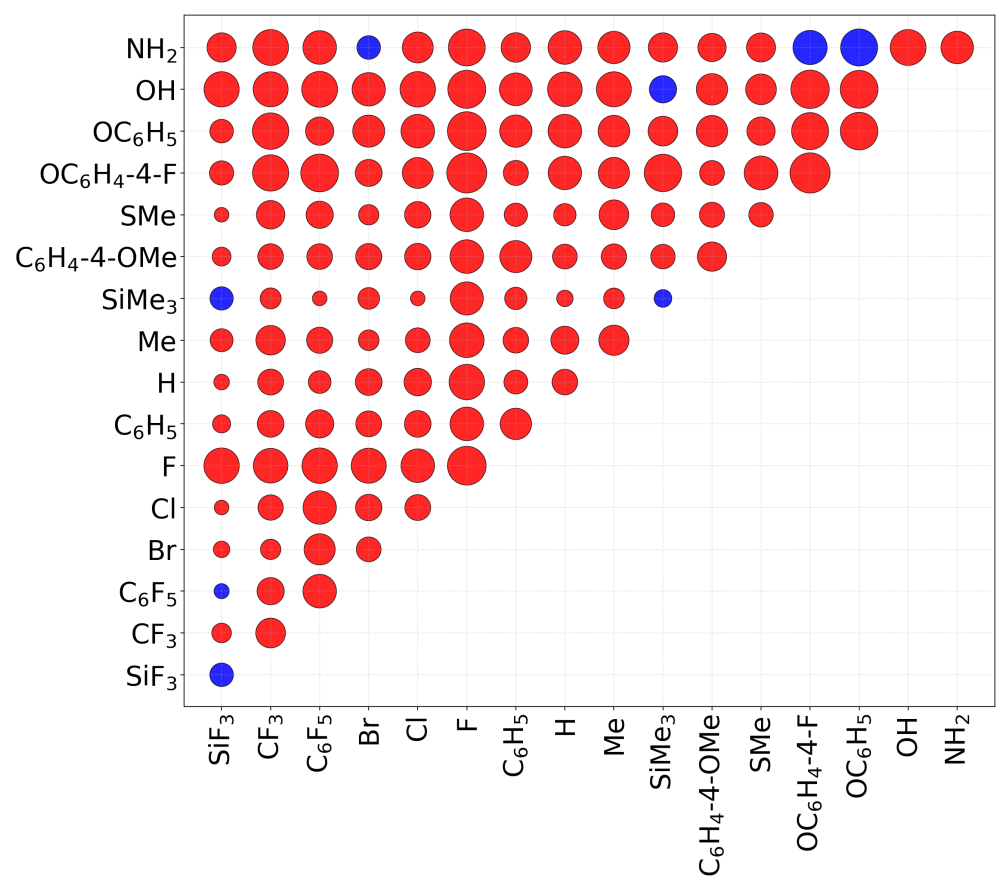

(a) Effect of substituents on the reaction energy difference, $\Delta\Delta G$ ($\Delta G_{\mathrm{Me_3P}} - \Delta G_{\mathrm{NH_3}}$), when R^3 = NH_2.

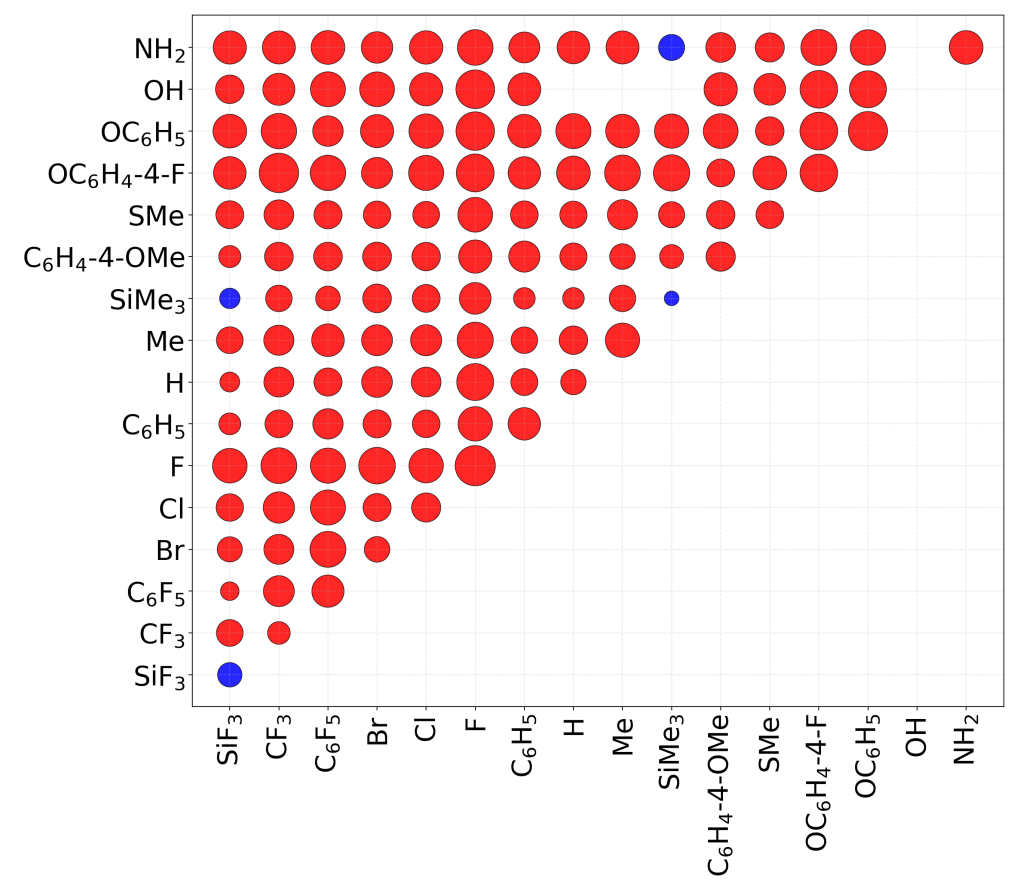

(b) Effect of substituents on the reaction energy difference, $\Delta\Delta G$ ($\Delta G_{\mathrm{Me_3P}} - \Delta G_{\mathrm{NH_3}}$), when R^3 = OH.

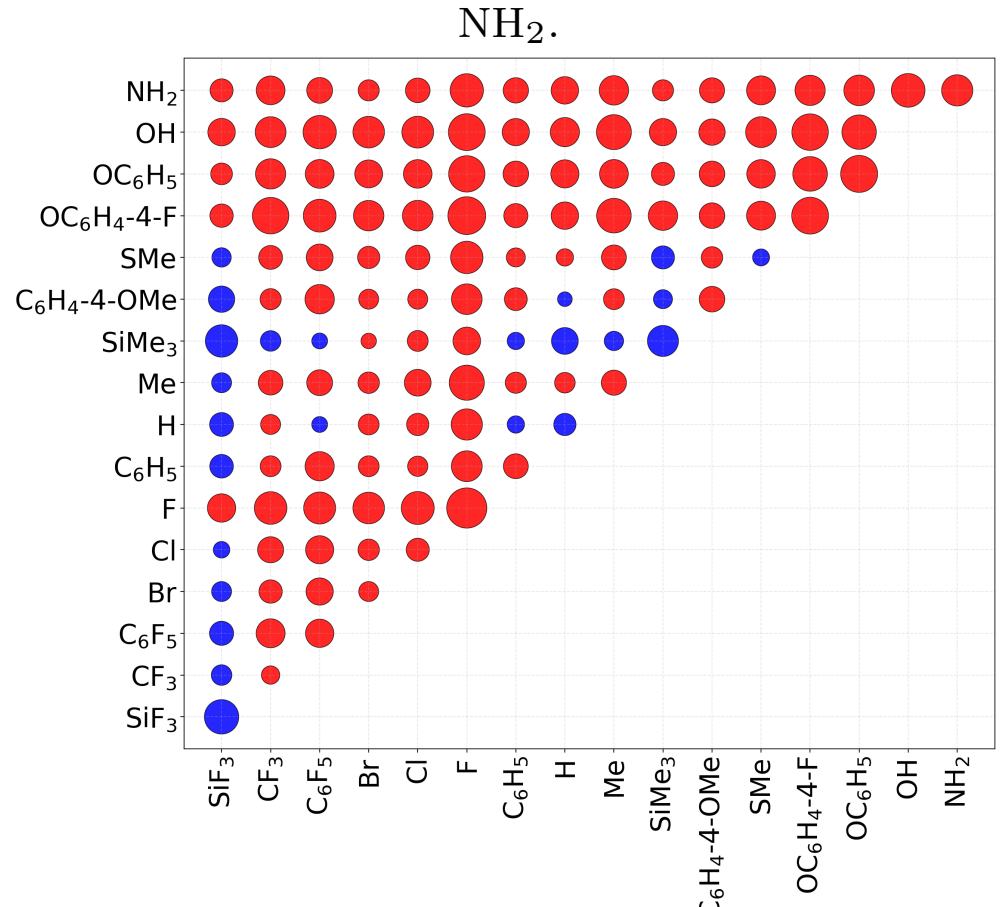

(c) Effect of substituents on the reaction energy difference, $\Delta\Delta G$ ($\Delta G_{\mathrm{Me_3P}} - \Delta G_{\mathrm{NH_3}}$), when R^3 = Me.

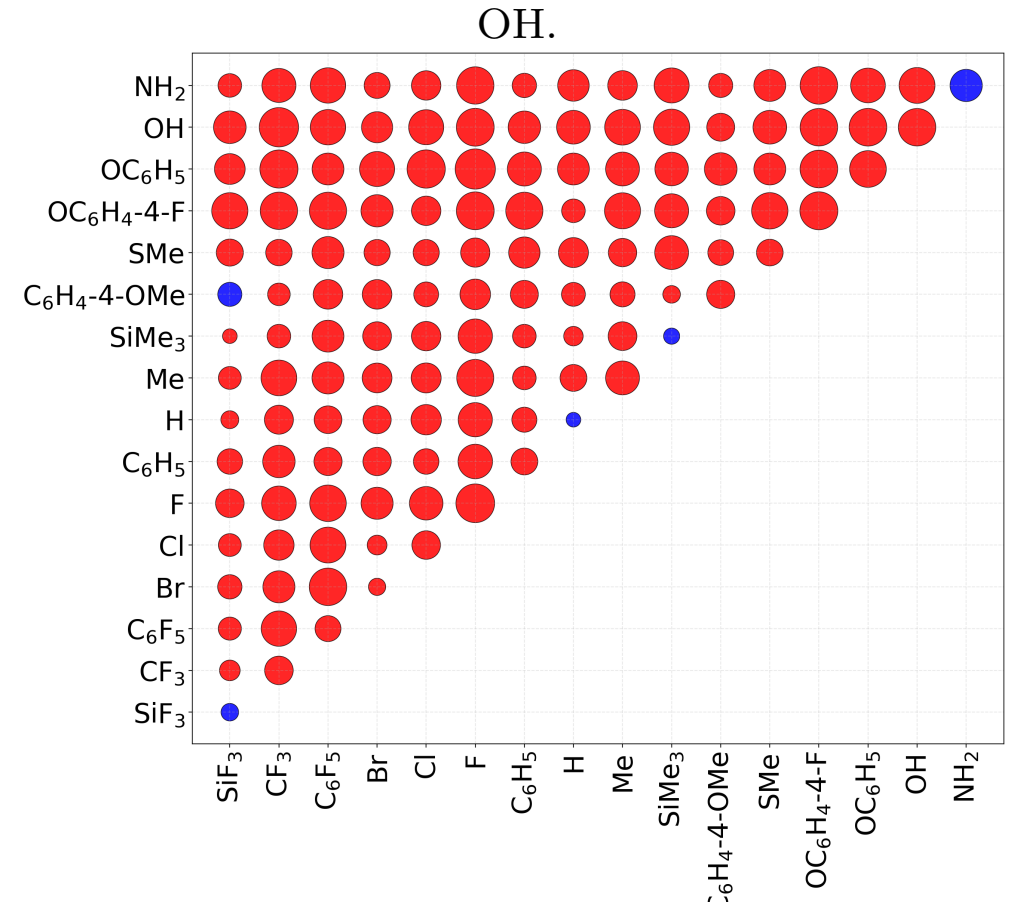

(d) Effect of substituents on the reaction energy difference, $\Delta\Delta G$ ($\Delta G_{\mathrm{Me_3P}} - \Delta G_{\mathrm{NH_3}}$), when R^3 = OC_6H_4-4-F.

Figure S6: Effect of substituents on the reaction energy difference, $\Delta\Delta G$ ($\Delta G_{\mathrm{Me_3P}} - \Delta G_{\mathrm{NH_3}}$). Positive $\Delta\Delta G$ values indicate a more favorable reaction with NH_3 as the Lewis base (represented by red circles), whereas negative $\Delta\Delta G$ values correspond to a greater stabilization with the Me_3P (represented by blue circles). The diameter of each circle is proportional to the magnitude of $\Delta\Delta G$, with smaller circles indicating minor differences in reaction energies between the two bases.

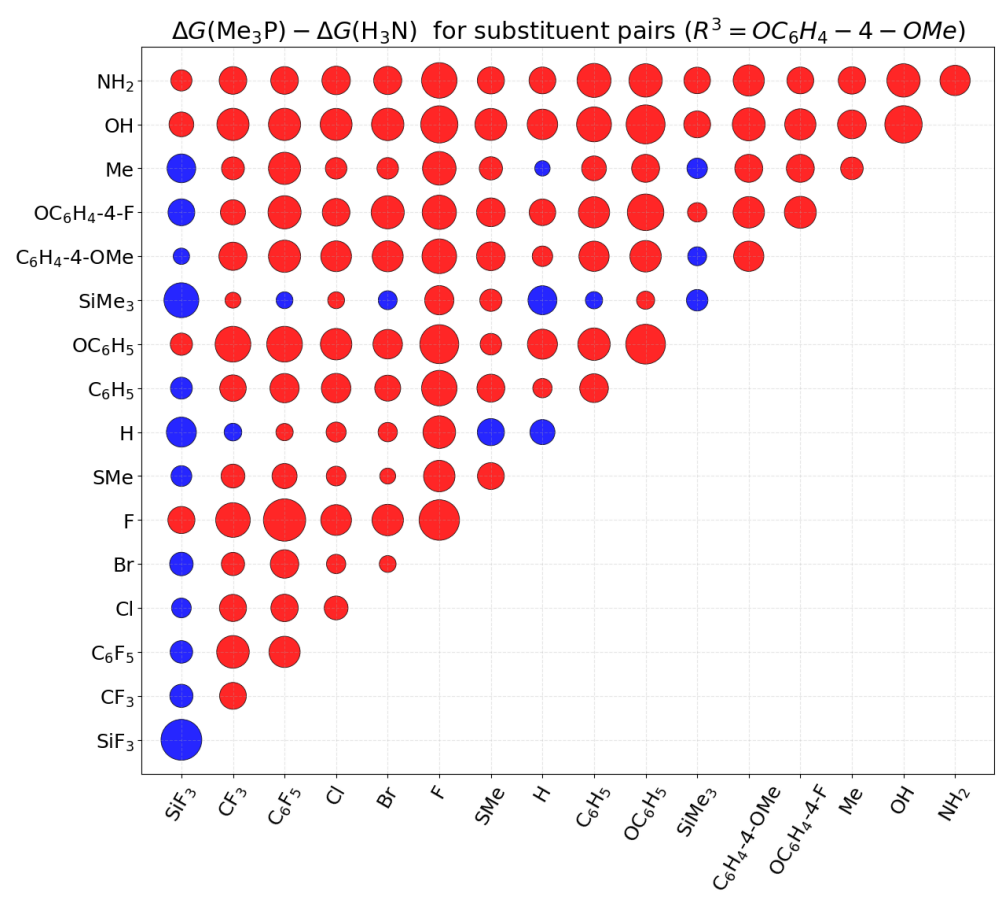

(a) Effect of substituents on the reaction energy difference, $\Delta\Delta G$ ($\Delta G_{\mathrm{Me_3P}} - \Delta G_{\mathrm{NH_3}}$), when R^3 = $\mathrm{C_6H_4}$-4-OMe.

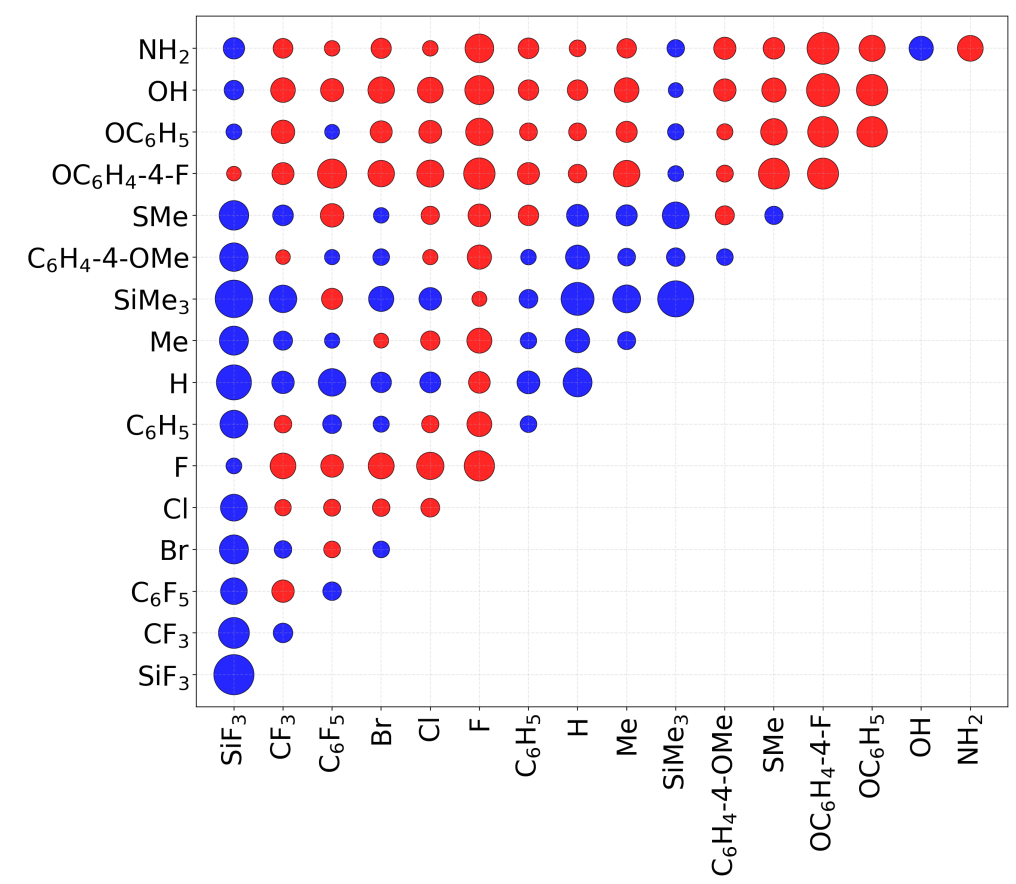

(b) Effect of substituents on the reaction energy difference, $\Delta\Delta G$ ($\Delta G_{\mathrm{Me_3P}} - \Delta G_{\mathrm{NH_3}}$), when R^3 = $\mathrm{SiMe_3}$.

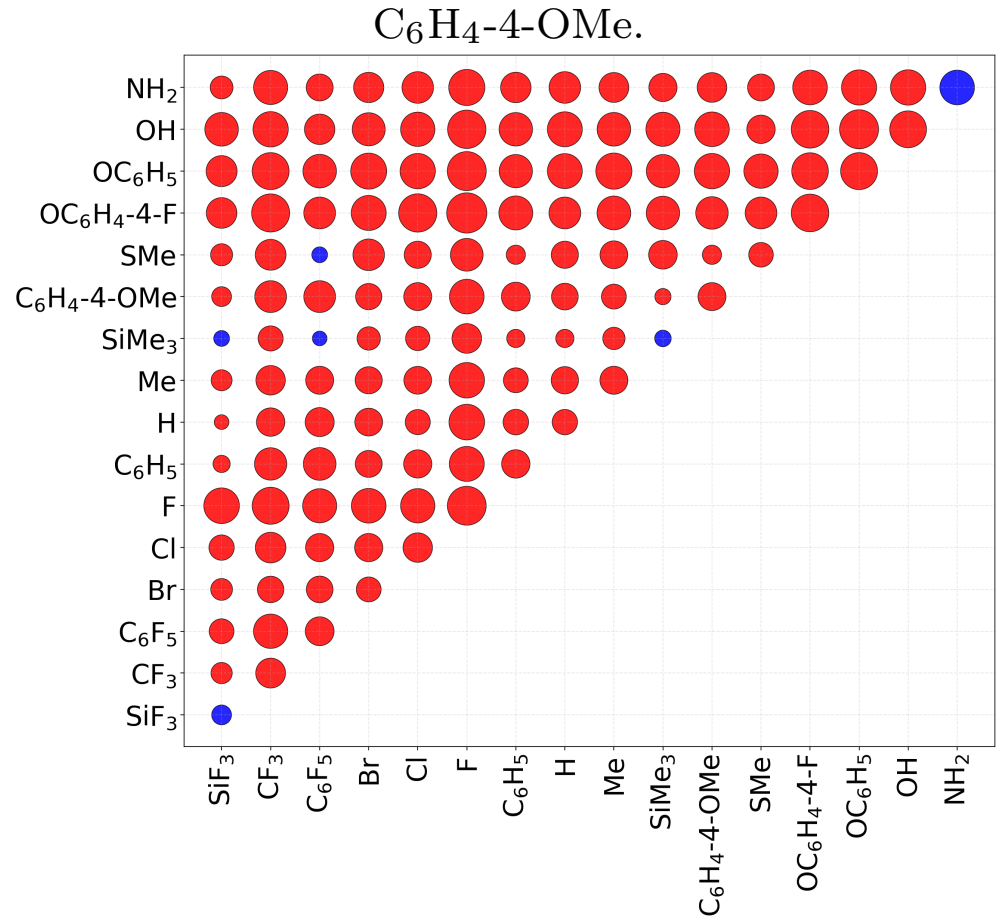

(c) Effect of substituents on the reaction energy difference, $\Delta\Delta G$ ($\Delta G_{\mathrm{Me_3P}} - \Delta G_{\mathrm{NH_3}}$), when R^3 = $\mathrm{OC_6H_5}$.

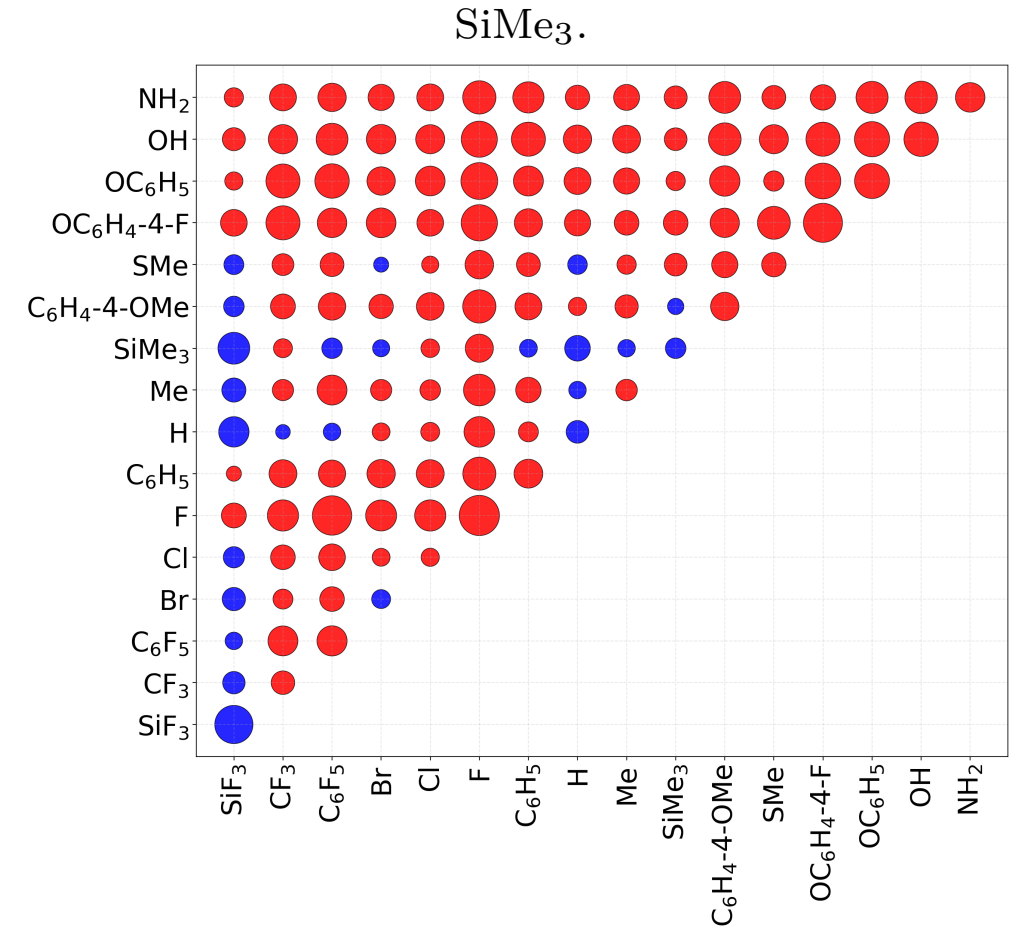

(d) Effect of substituents on the reaction energy difference, $\Delta\Delta G$ ($\Delta G_{\mathrm{Me_3P}} - \Delta G_{\mathrm{NH_3}}$), when R^3 = $\mathrm{C_6H_5}$.

Figure S7: Effect of substituents on the reaction energy difference, $\Delta\Delta G$ ($\Delta G_{\mathrm{Me_3P}} - \Delta G_{\mathrm{NH_3}}$). Positive $\Delta\Delta G$ values indicate a more favorable reaction with $\mathrm{NH_3}$ as the Lewis base (represented by red circles), whereas negative $\Delta\Delta G$ values correspond to a greater stabilization with the Me_3P (represented by blue circles). The diameter of each circle is proportional to the magnitude of $\Delta\Delta G$, with smaller circles indicating minor differences in reaction energies between the two bases.

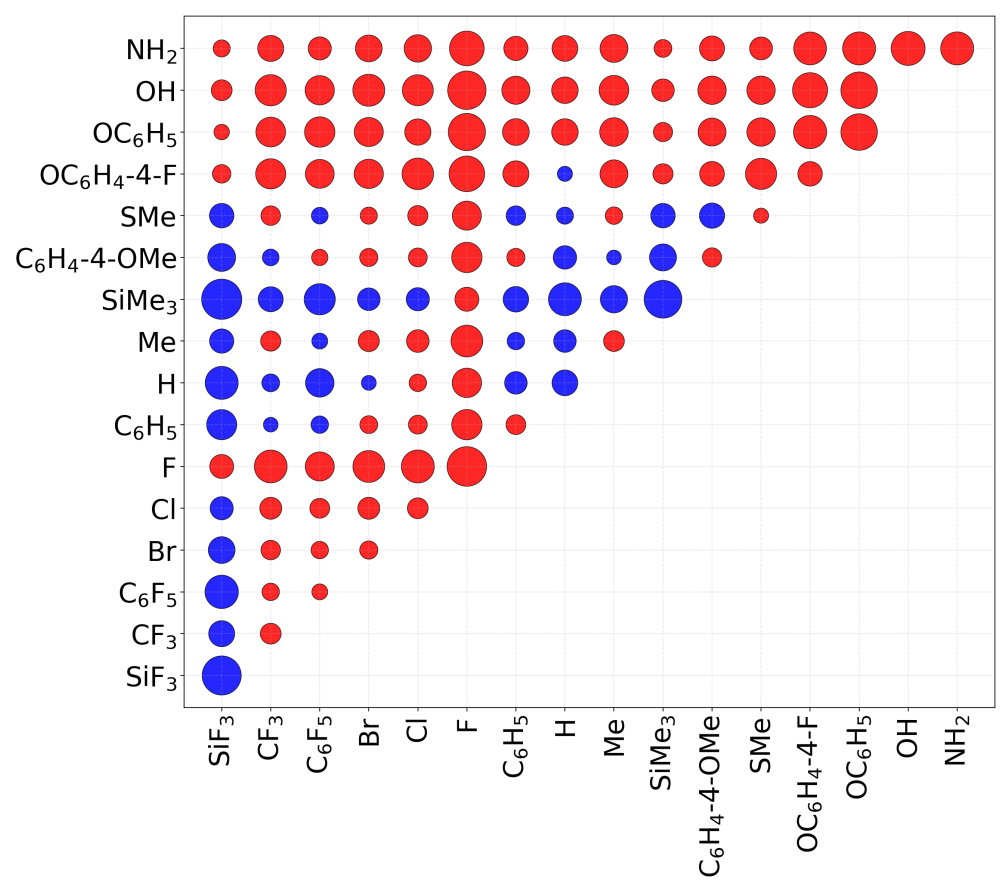

(a) Effect of substituents on the reaction energy difference, $\Delta\Delta G$ ($\Delta G_{\mathrm{Me_3P}} - \Delta G_{\mathrm{NH_3}}$), when R^3 = H.

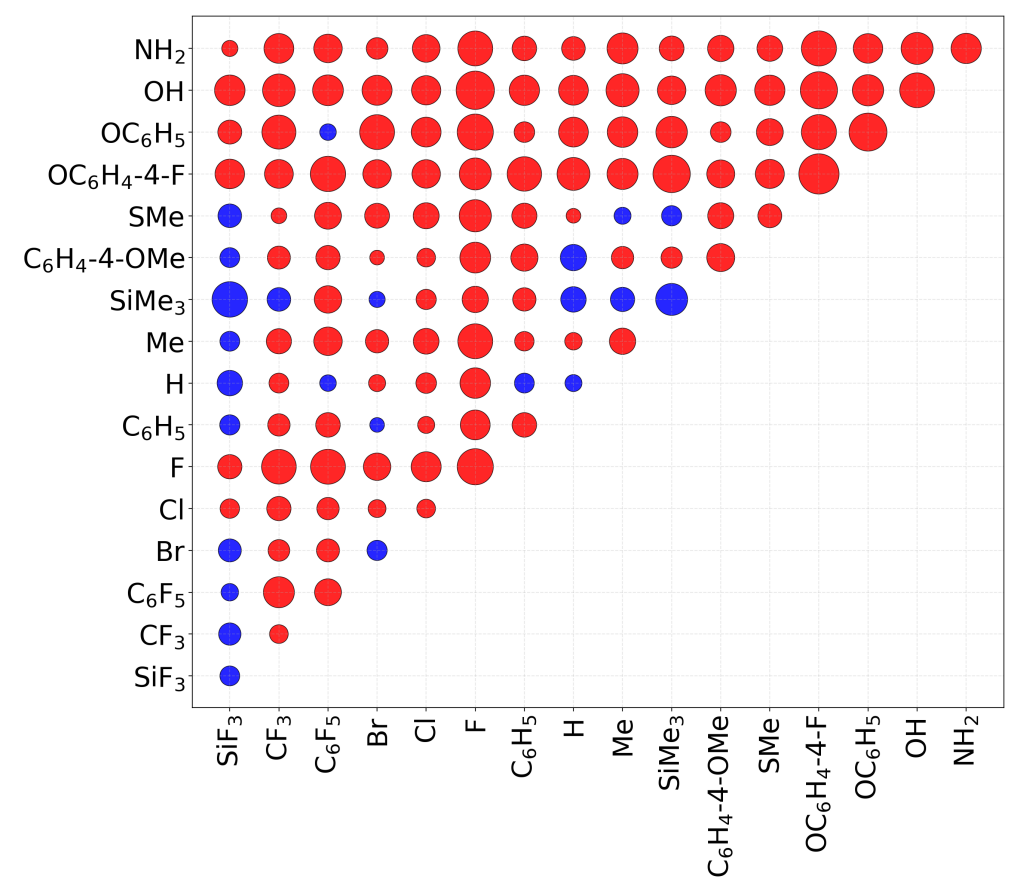

(b) Effect of substituents on the reaction energy difference, $\Delta\Delta G$ ($\Delta G_{\mathrm{Me_3P}} - \Delta G_{\mathrm{NH_3}}$), when R^3 = SMe.

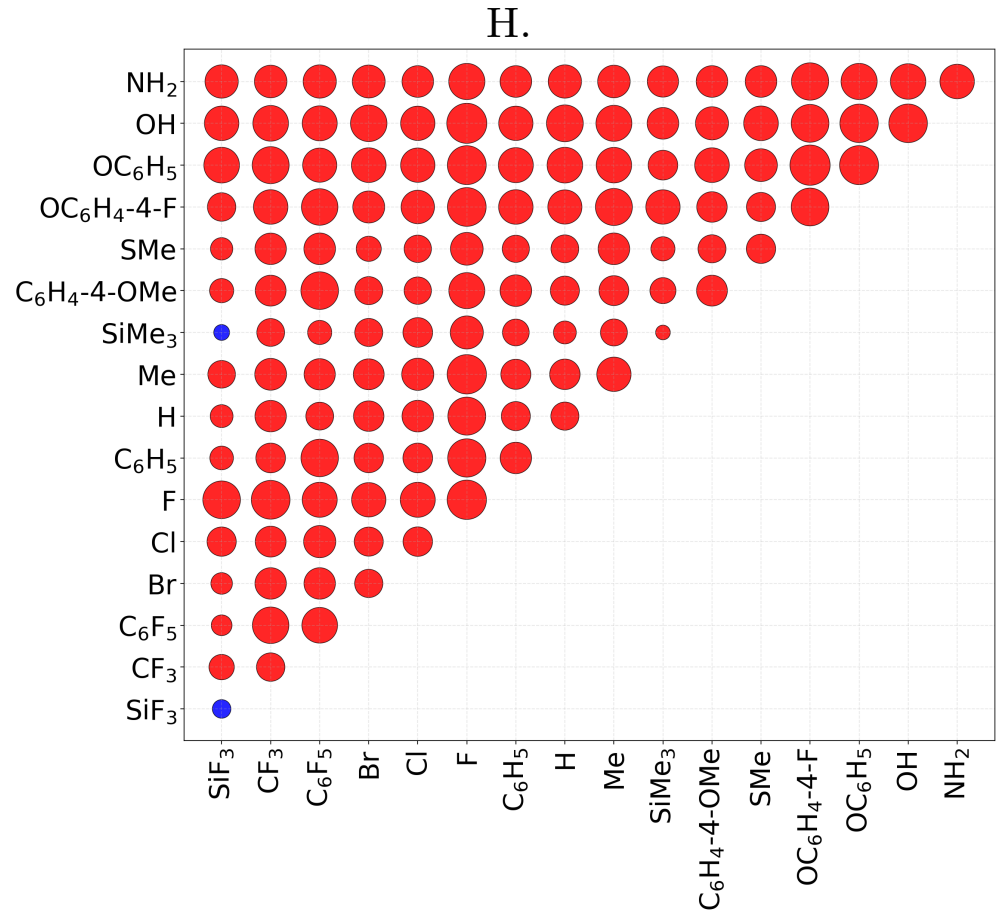

(c) Effect of substituents on the reaction energy difference, $\Delta\Delta G$ ($\Delta G_{\mathrm{Me_3P}} - \Delta G_{\mathrm{NH_3}}$), when R^3 = F.

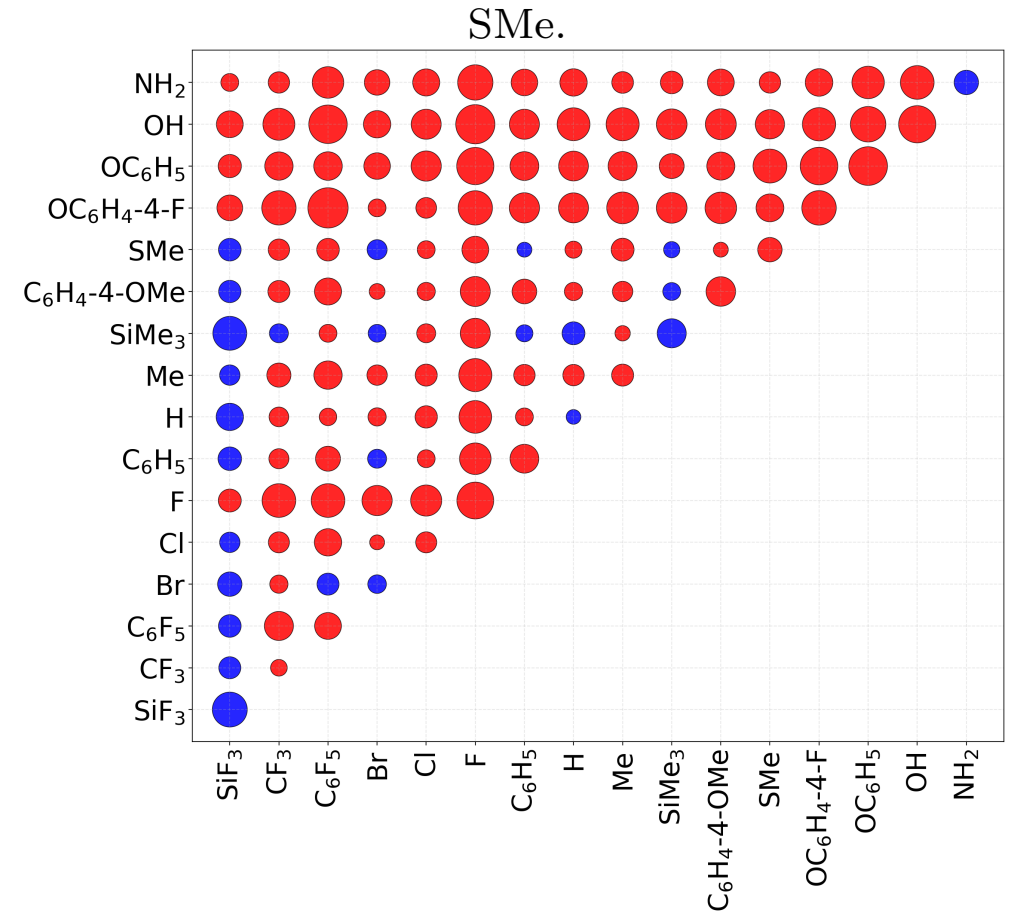

(d) Effect of substituents on the reaction energy difference, $\Delta\Delta G$ ($\Delta G_{\mathrm{Me_3P}} - \Delta G_{\mathrm{NH_3}}$), when R^3 = Br.

Figure S8: Effect of substituents on the reaction energy difference, $\Delta\Delta G$ ($\Delta G_{\mathrm{Me_3P}} - \Delta G_{\mathrm{NH_3}}$). Positive $\Delta\Delta G$ values indicate a more favorable reaction with NH_3 as the Lewis base (represented by red circles), whereas negative $\Delta\Delta G$ values correspond to a greater stabilization with the Me_3P (represented by blue circles). The diameter of each circle is proportional to the magnitude of $\Delta\Delta G$, with smaller circles indicating minor differences in reaction energies between the two bases.

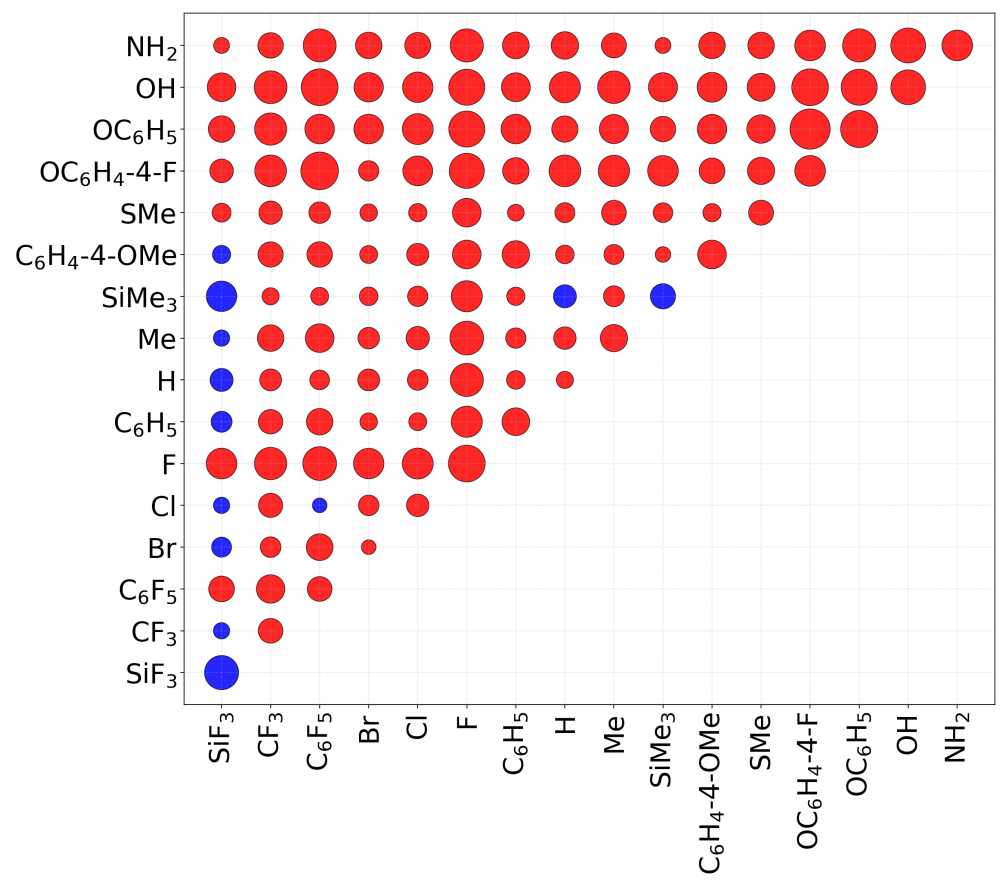

(a) Effect of substituents on the reaction energy difference, $\Delta\Delta G$ ($\Delta G_{\mathrm{Me_3P}} - \Delta G_{\mathrm{NH_3}}$), when R^3 = Cl.

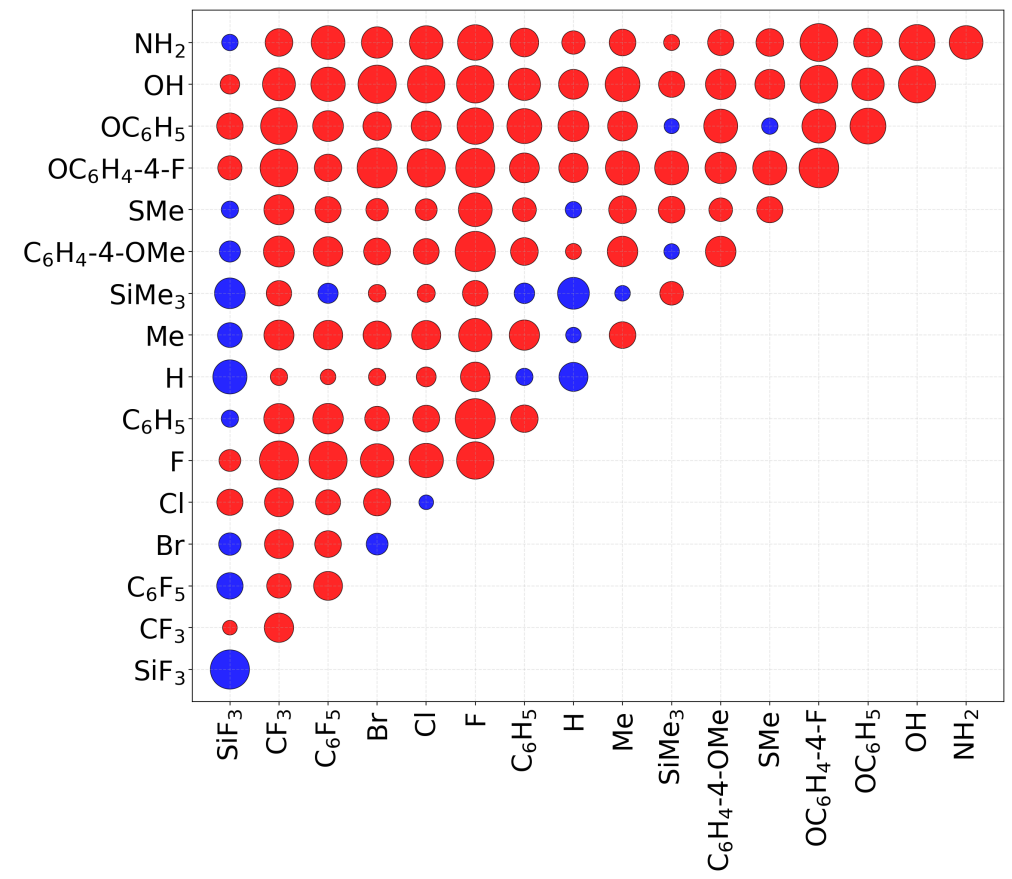

(b) Effect of substituents on the reaction energy difference, $\Delta\Delta G$ ($\Delta G_{\mathrm{Me_3P}} - \Delta G_{\mathrm{NH_3}}$), when R^3 = C_6F_5.

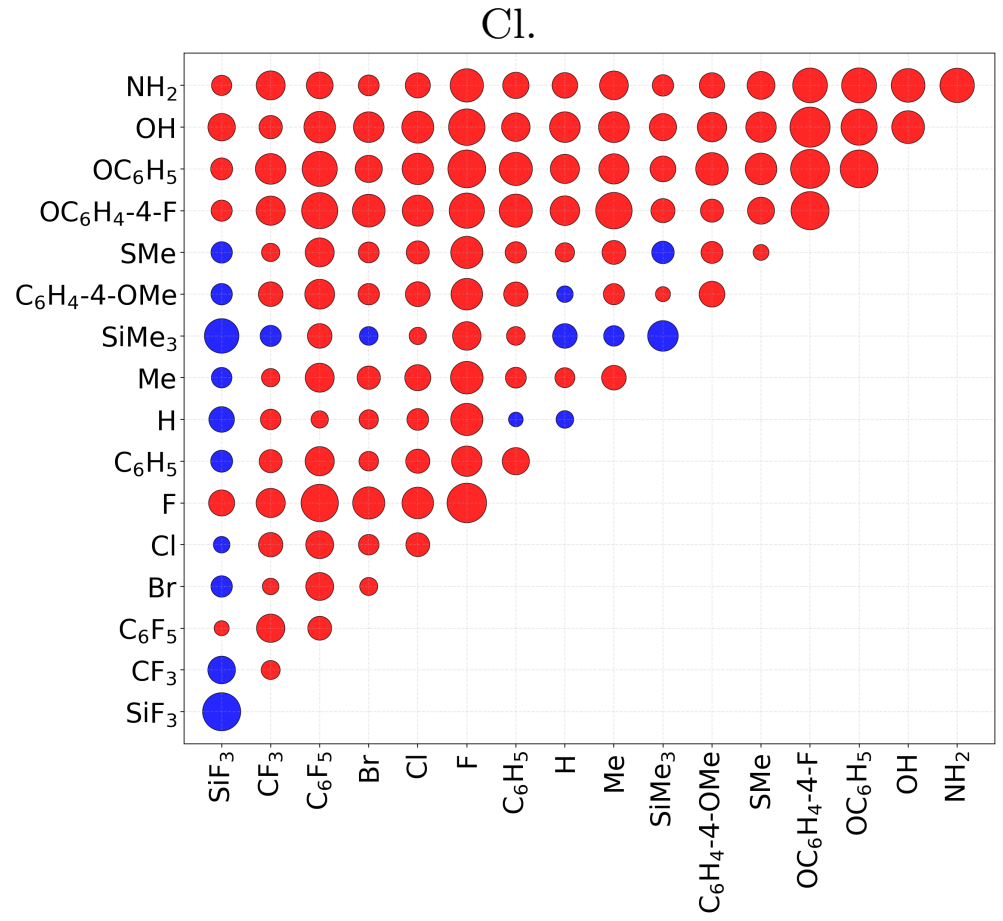

(c) Effect of substituents on the reaction energy difference, $\Delta\Delta G$ ($\Delta G_{\mathrm{Me_3P}} - \Delta G_{\mathrm{NH_3}}$), when R^3 = CF_3.

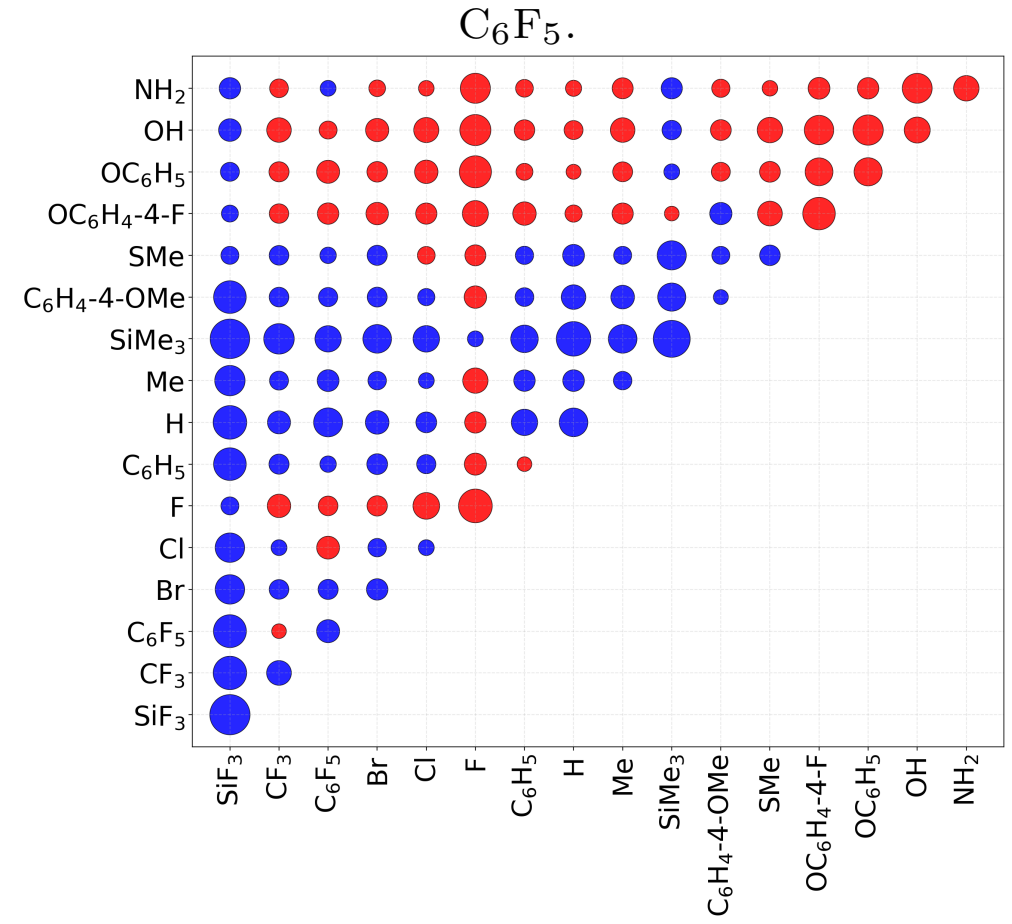

(d) Effect of substituents on the reaction energy difference, $\Delta\Delta G$ ($\Delta G_{\mathrm{Me_3P}} - \Delta G_{\mathrm{NH_3}}$), when R^3 = SiF_3.

Figure S9: Effect of substituents on the reaction energy difference, $\Delta\Delta G$ ($\Delta G_{\mathrm{Me_3P}} - \Delta G_{\mathrm{NH_3}}$). Positive $\Delta\Delta G$ values indicate a more favorable reaction with NH_3 as the Lewis base (represented by red circles), whereas negative $\Delta\Delta G$ values correspond to a greater stabilization with the Me_3P (represented by blue circles). The diameter of each circle is proportional to the magnitude of $\Delta\Delta G$, with smaller circles indicating minor differences in reaction energies between the two bases.

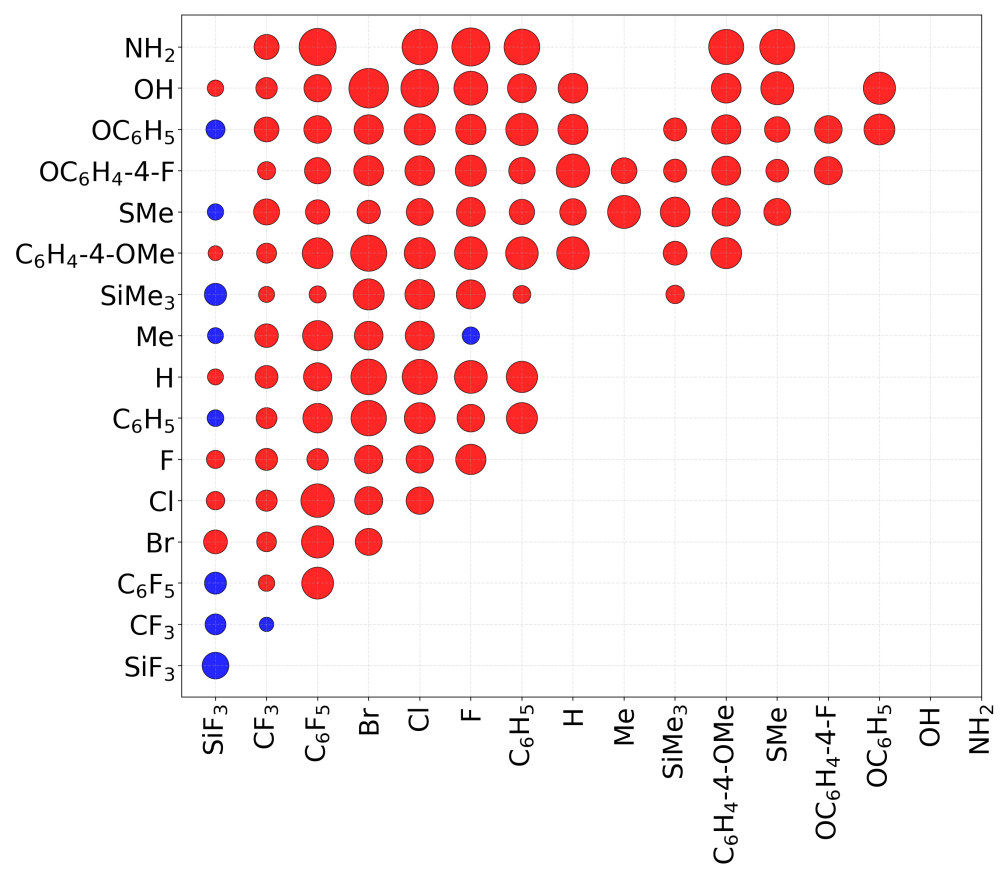

(a) Effect of substituents on the reaction energy difference, $\Delta\Delta G$ ($\Delta G_{\text{Cl}^-} - \Delta G_{\text{NH}_3}$), when R^3 = NH_2.

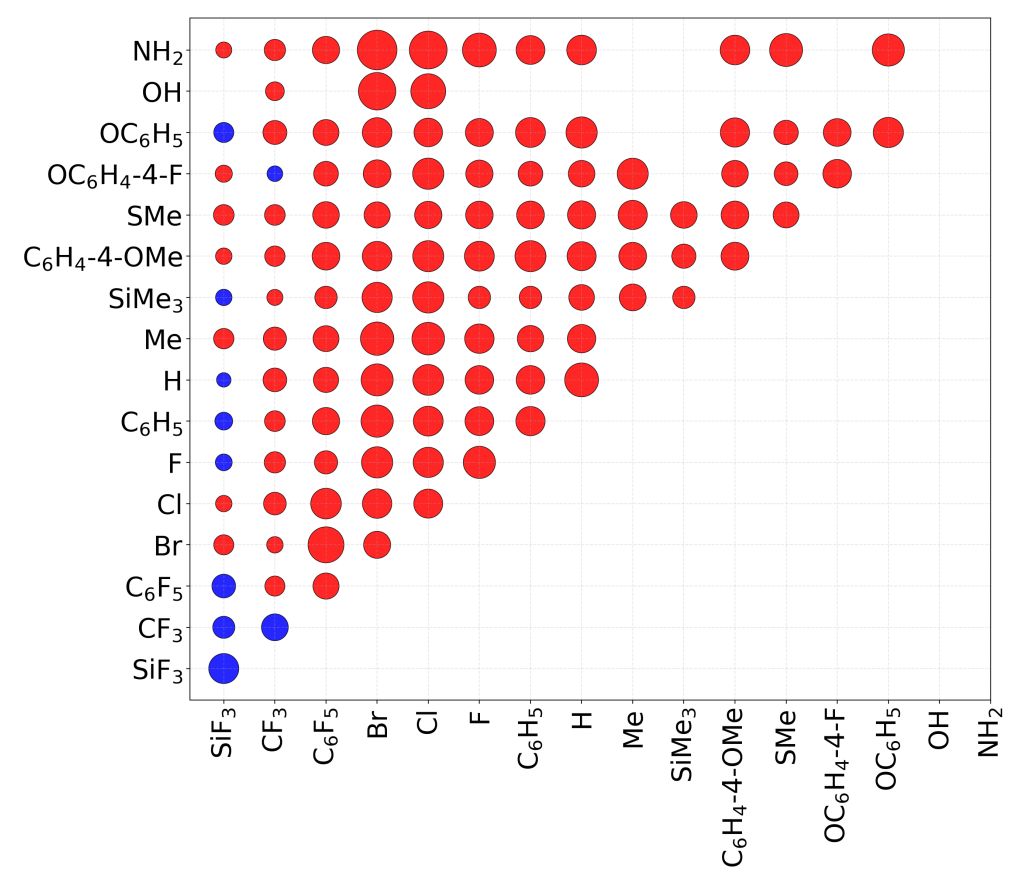

(b) Effect of substituents on the reaction energy difference, $\Delta\Delta G$ ($\Delta G_{\text{Cl}^-} - \Delta G_{\text{NH}_3}$), when R^3 = OH.

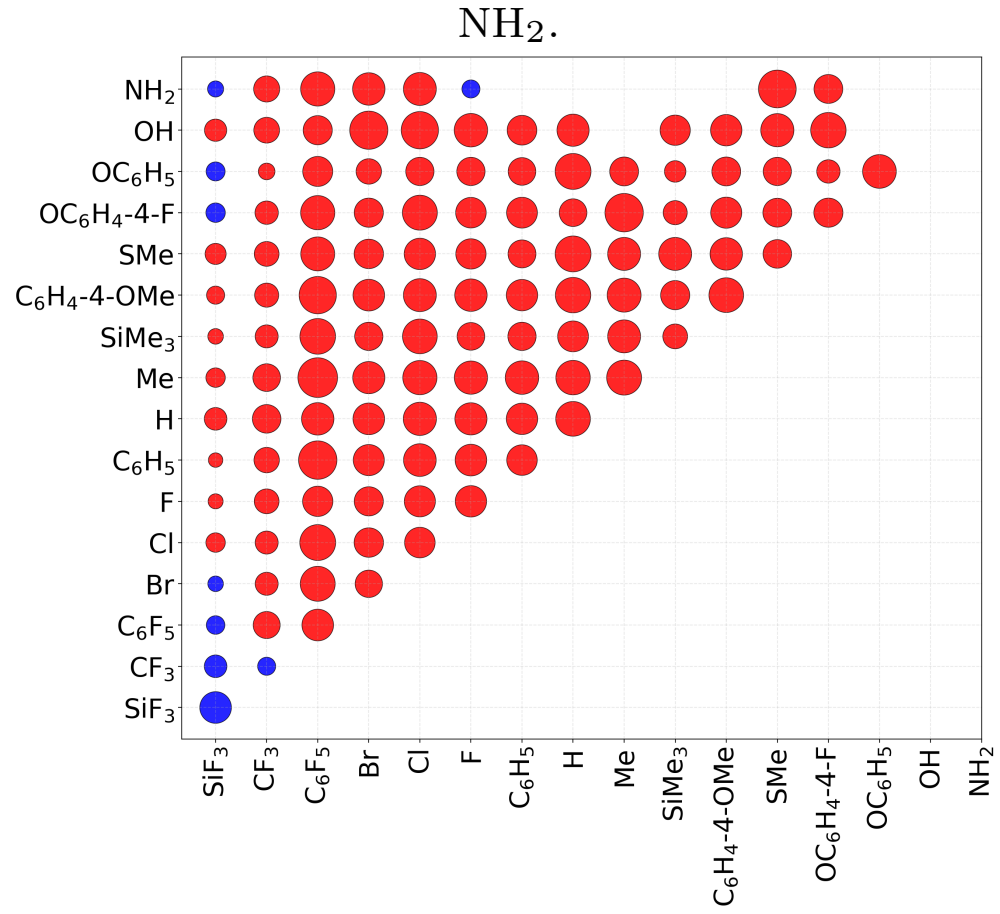

(c) Effect of substituents on the reaction energy difference, $\Delta\Delta G$ ($\Delta G_{\text{Cl}^-} - \Delta G_{\text{NH}_3}$), when R^3 = Me.

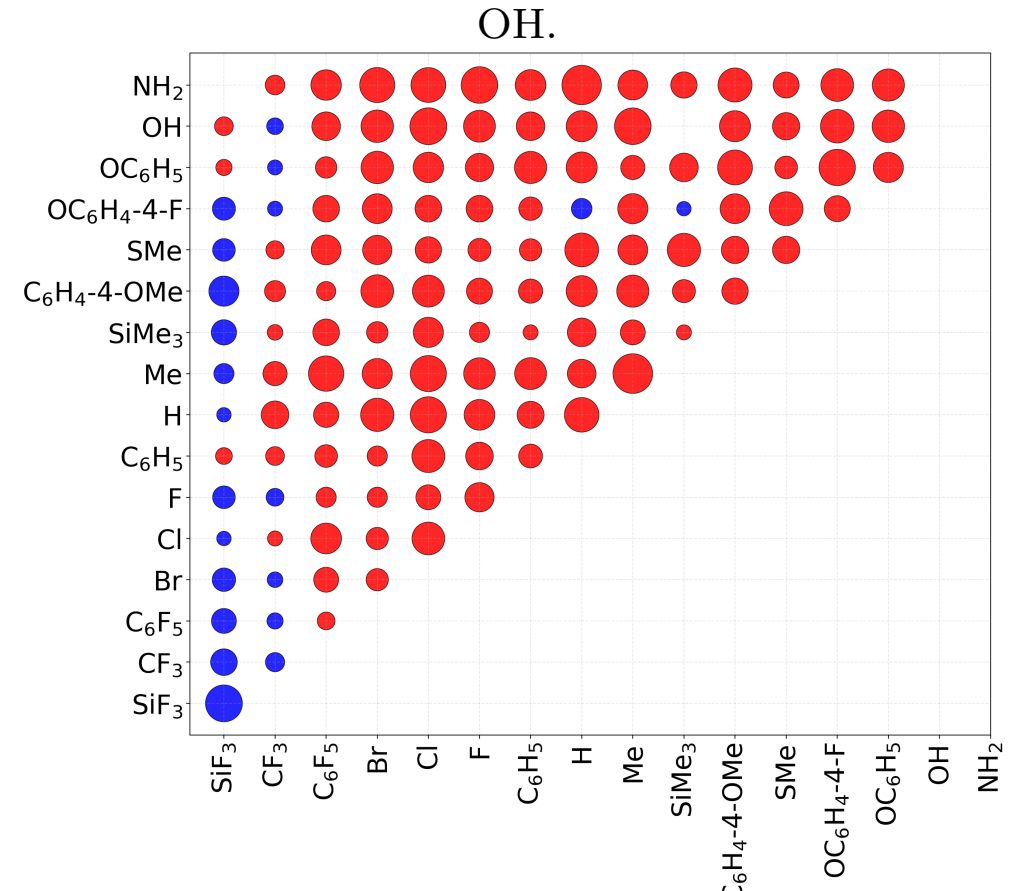

(d) Effect of substituents on the reaction energy difference, $\Delta\Delta G$ ($\Delta G_{\text{Cl}^-} - \Delta G_{\text{NH}_3}$), when R^3 = OC_6H_4-4-F.

Figure S10: Effect of substituents on the reaction energy difference, $\Delta\Delta G$ ($\Delta G_{\text{Cl}^-} - \Delta G_{\text{NH}_3}$). Positive $\Delta\Delta G$ values indicate a more favorable reaction with NH_3 as the Lewis base (represented by red circles), whereas negative $\Delta\Delta G$ values correspond to a greater stabilization with Cl^- (represented by blue circles). The diameter of each circle is proportional to the magnitude of $\Delta\Delta G$, with smaller circles indicating minor differences in reaction energies between the two bases.

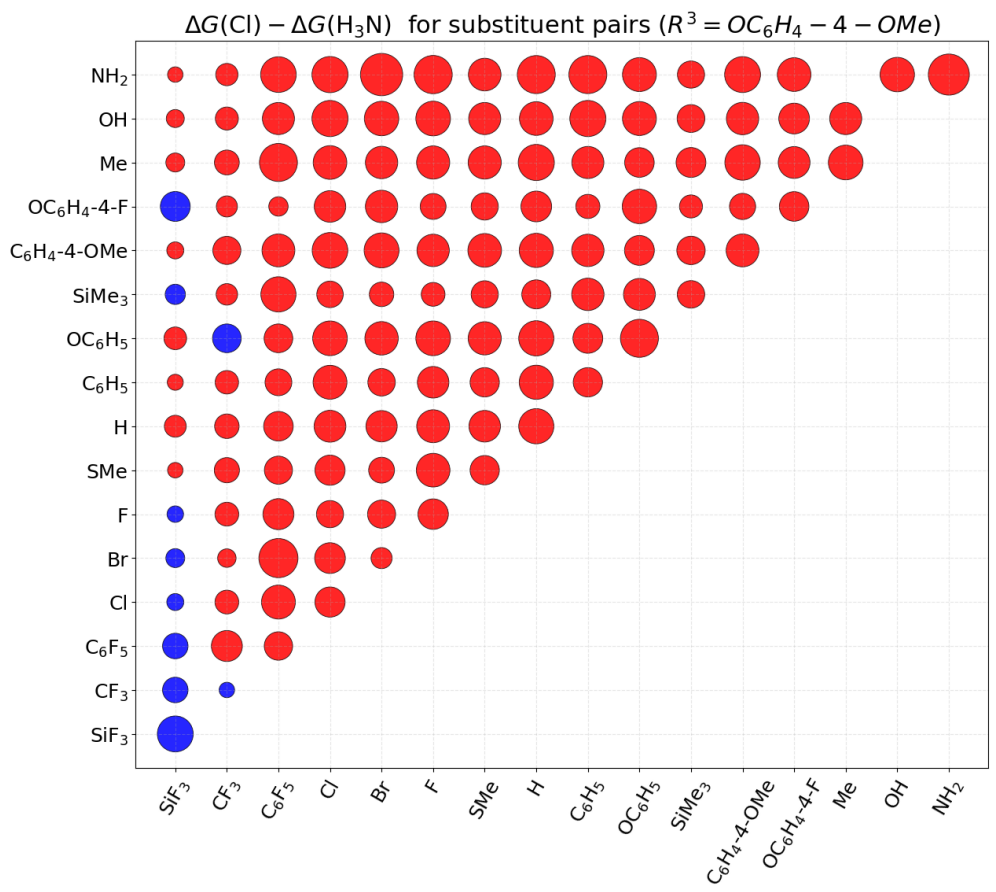

(a) Effect of substituents on the reaction energy difference, $\Delta\Delta G$ ($\Delta G_{\mathrm{Cl}^-} - \Delta G_{\mathrm{NH_3}}$), when R^3 = C_6H_4-4-OMe.

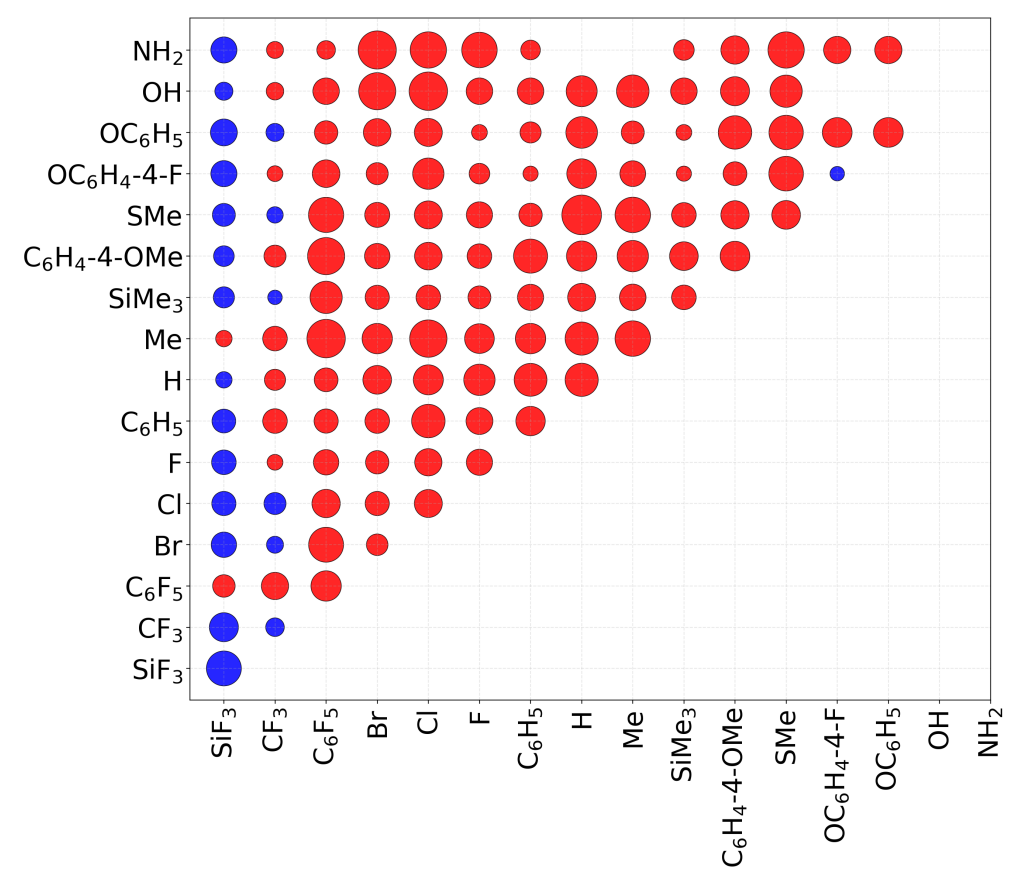

(b) Effect of substituents on the reaction energy difference, $\Delta\Delta G$ ($\Delta G_{\mathrm{Cl}^-} - \Delta G_{\mathrm{NH_3}}$), when R^3 = $SiMe_3$.

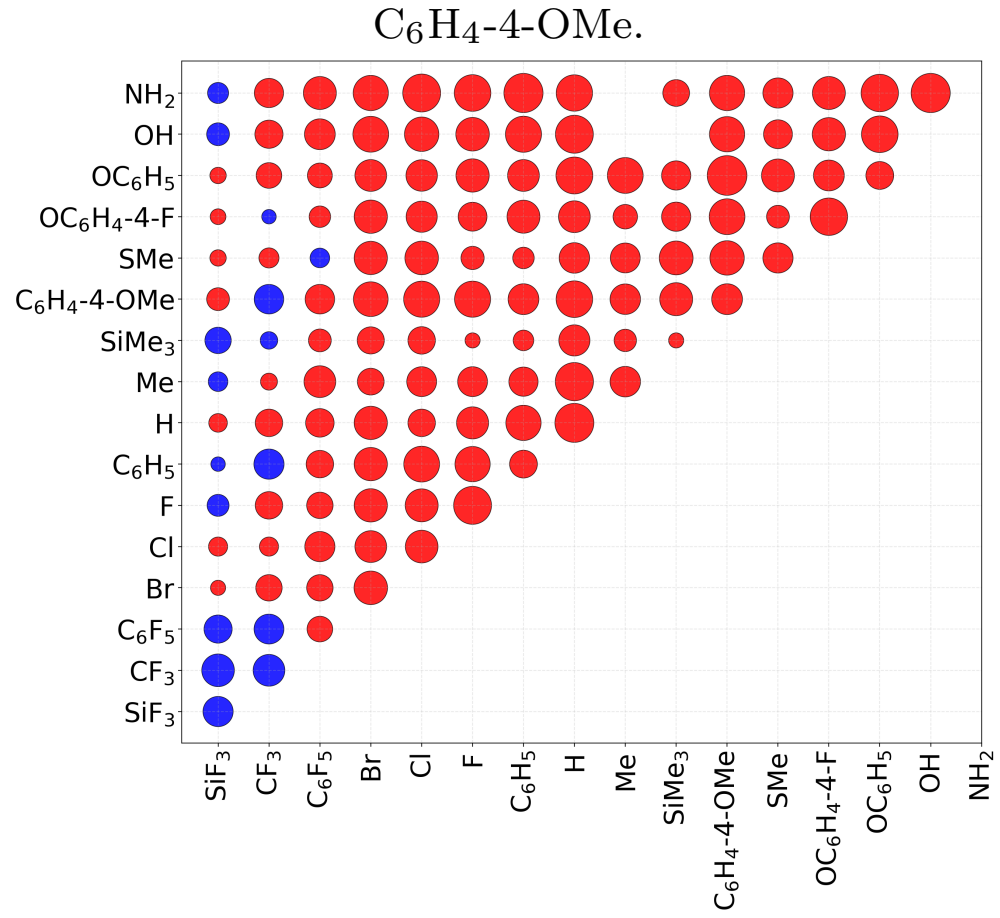

(c) Effect of substituents on the reaction energy difference, $\Delta\Delta G$ ($\Delta G_{\mathrm{Cl}^-} - \Delta G_{\mathrm{NH_3}}$), when R^3 = OC_6H_5.

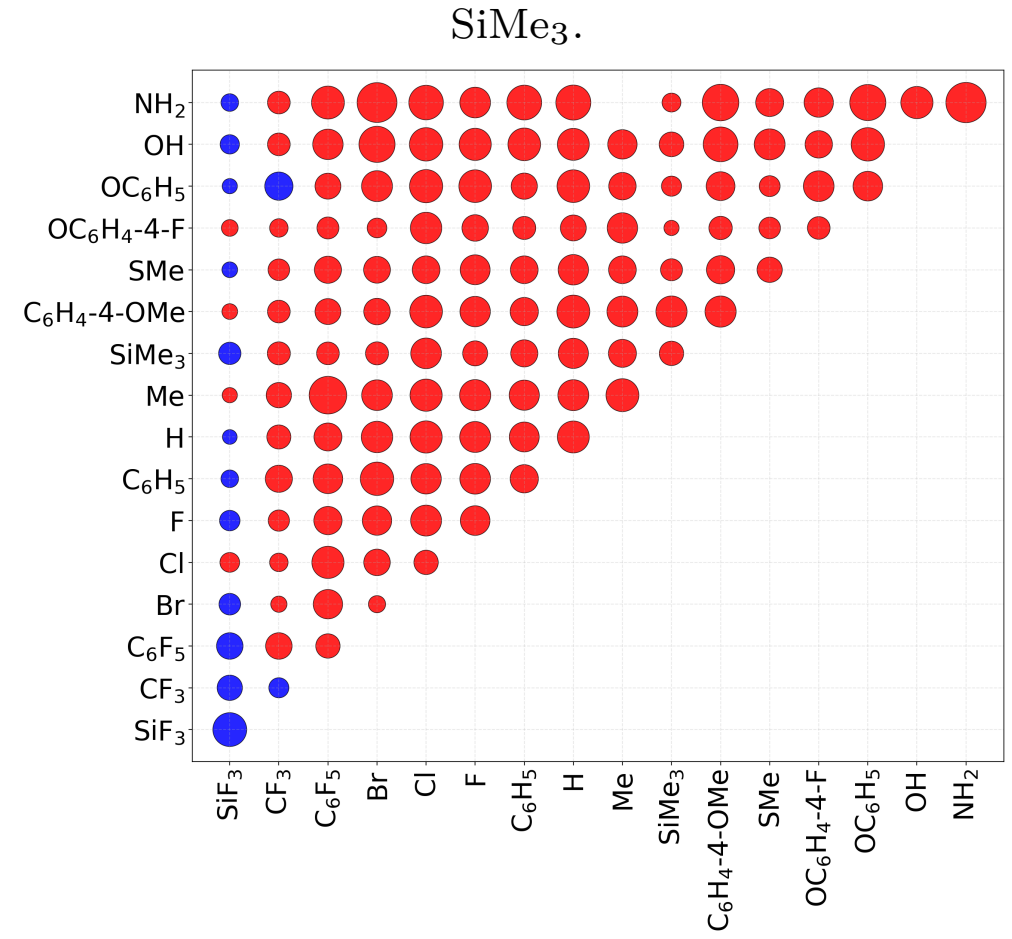

(d) Effect of substituents on the reaction energy difference, $\Delta\Delta G$ ($\Delta G_{\mathrm{Cl}^-} - \Delta G_{\mathrm{NH_3}}$), when R^3 = C_6H_5.

Figure S11: Effect of substituents on the reaction energy difference, $\Delta\Delta G$ ($\Delta G_{\mathrm{Cl}^-} - \Delta G_{\mathrm{NH_3}}$). Positive $\Delta\Delta G$ values indicate a more favorable reaction with NH_3 as the Lewis base (represented by red circles), whereas negative $\Delta\Delta G$ values correspond to a greater stabilization with the Cl^- (represented by blue circles). The diameter of each circle is proportional to the magnitude of $\Delta\Delta G$, with smaller circles indicating minor differences in reaction energies between the two bases.

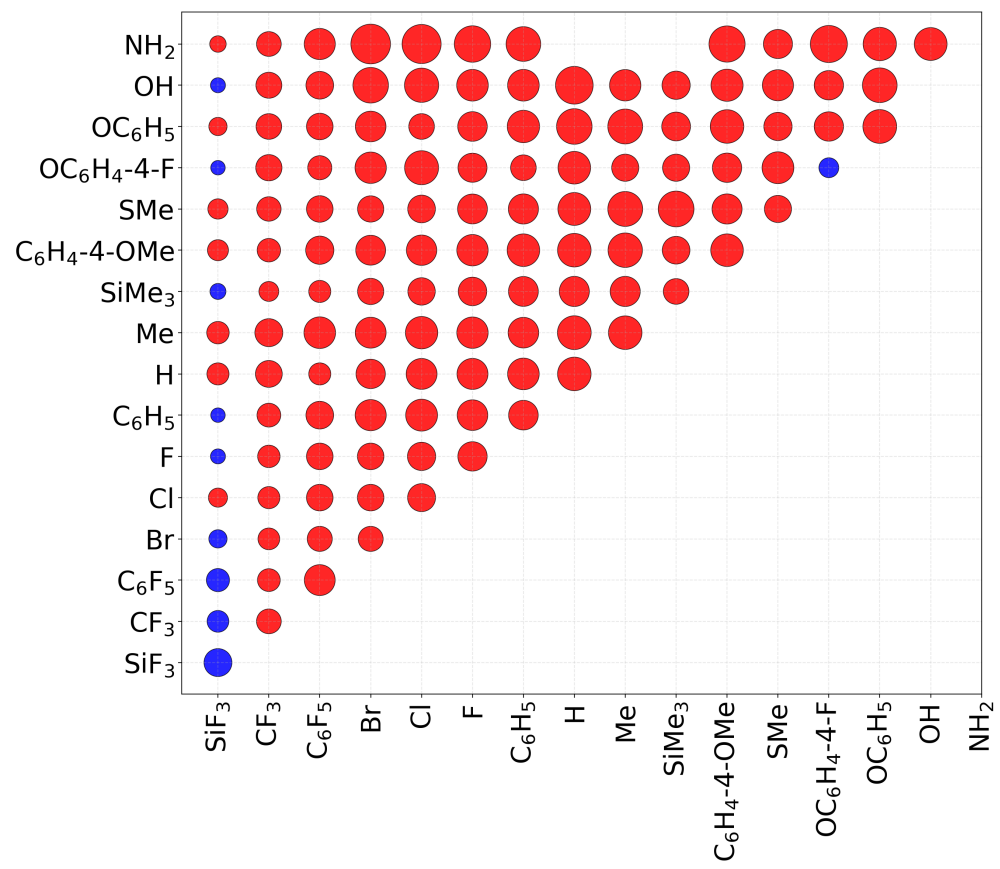

(a) Effect of substituents on the reaction energy difference, $\Delta\Delta G$ ($\Delta G_{\mathrm{Cl}^-} - \Delta G_{\mathrm{NH_3}}$), when R^3 = H.

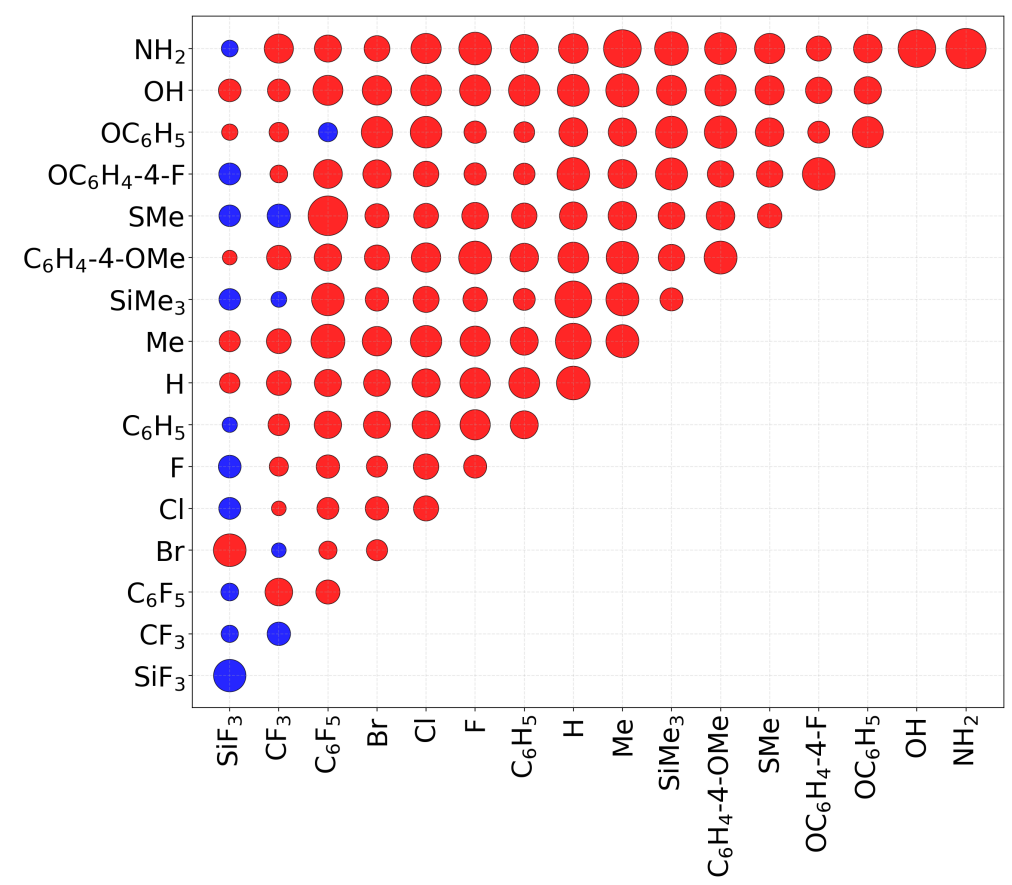

(b) Effect of substituents on the reaction energy difference, $\Delta\Delta G$ ($\Delta G_{\mathrm{Cl}^-} - \Delta G_{\mathrm{NH_3}}$), when R^3 = SMe.

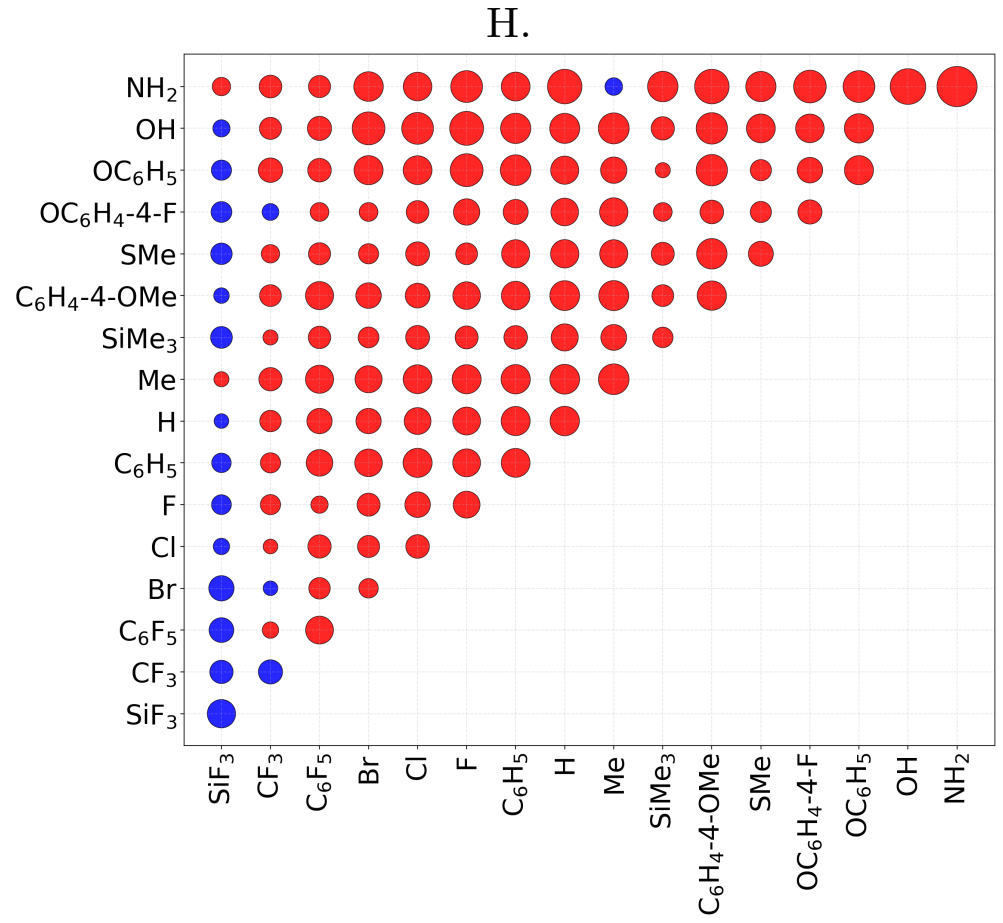

(c) Effect of substituents on the reaction energy difference, $\Delta\Delta G$ ($\Delta G_{\mathrm{Cl}^-} - \Delta G_{\mathrm{NH_3}}$), when R^3 = F.

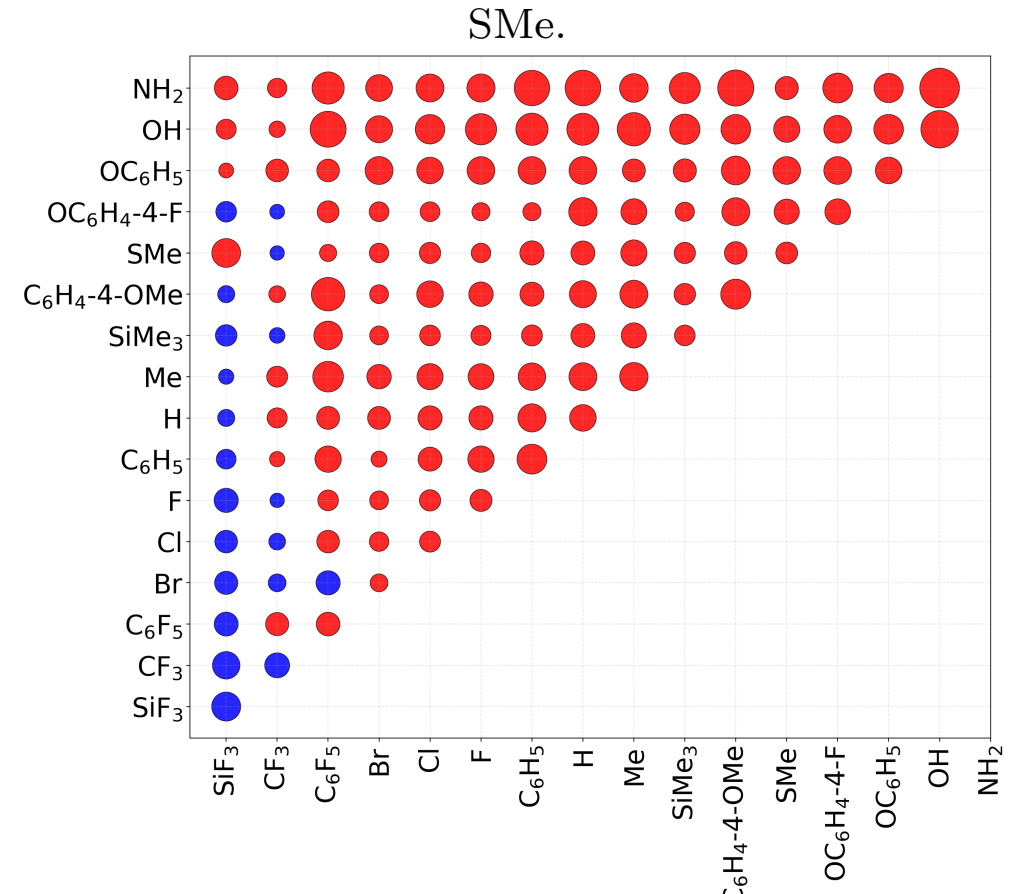

(d) Effect of substituents on the reaction energy difference, $\Delta\Delta G$ ($\Delta G_{\mathrm{Cl}^-} - \Delta G_{\mathrm{NH_3}}$), when R^3 = Br.

Figure S12: Effect of substituents on the reaction energy difference, $\Delta\Delta G$ ($\Delta G_{\mathrm{Cl}^-} - \Delta G_{\mathrm{NH_3}}$). Positive $\Delta\Delta G$ values indicate a more favorable reaction with NH_3 as the Lewis base (represented by red circles), whereas negative $\Delta\Delta G$ values correspond to a greater stabilization with the Cl^- (represented by blue circles). The diameter of each circle is proportional to the magnitude of $\Delta\Delta G$, with smaller circles indicating minor differences in reaction energies between the two bases.

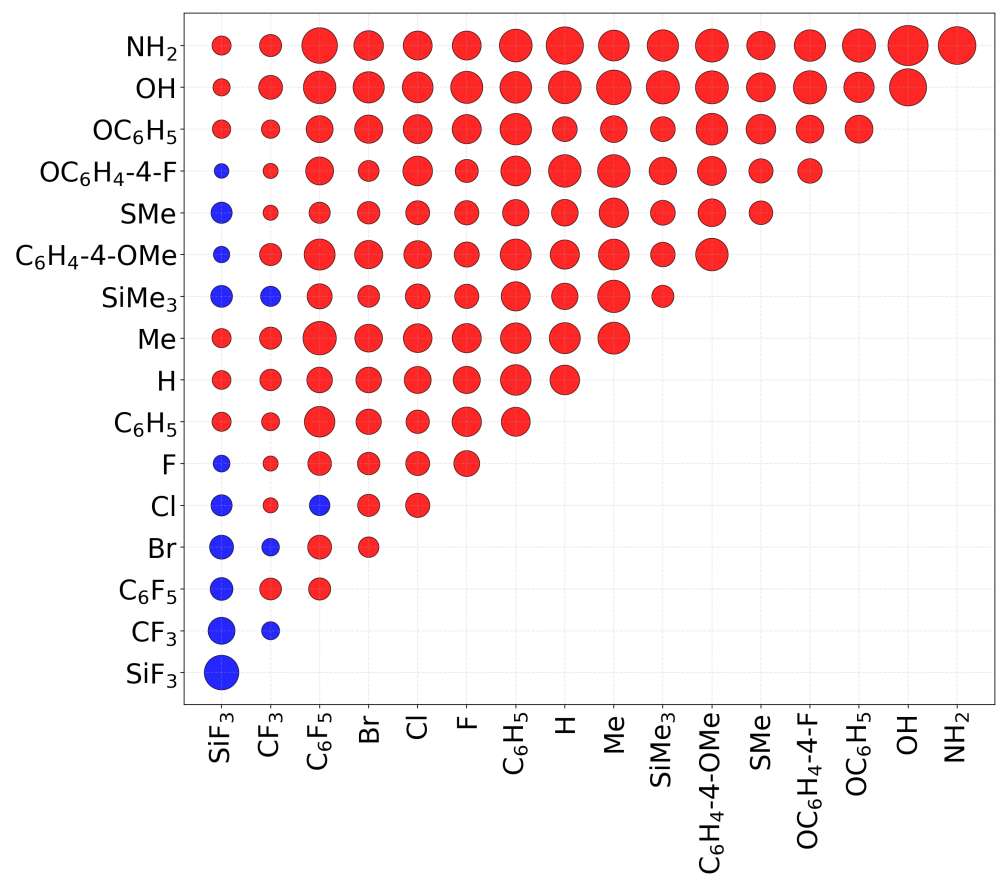

(a) Effect of substituents on the reaction energy difference, $\Delta\Delta G$ ($\Delta G_{\text{Cl}^-} - \Delta G_{\text{NH}_3}$), when R^3 = Cl.

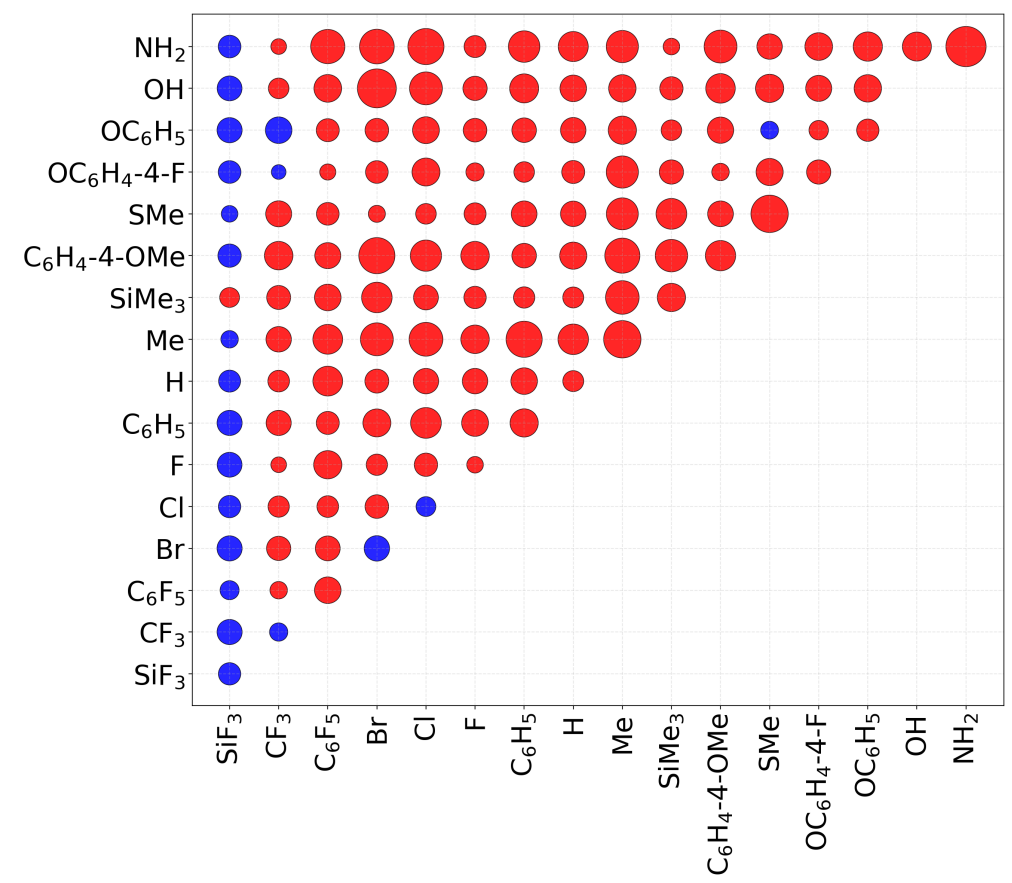

(b) Effect of substituents on the reaction energy difference, $\Delta\Delta G$ ($\Delta G_{\text{Cl}^-} - \Delta G_{\text{NH}_3}$), when R^3 = C_6F_5.

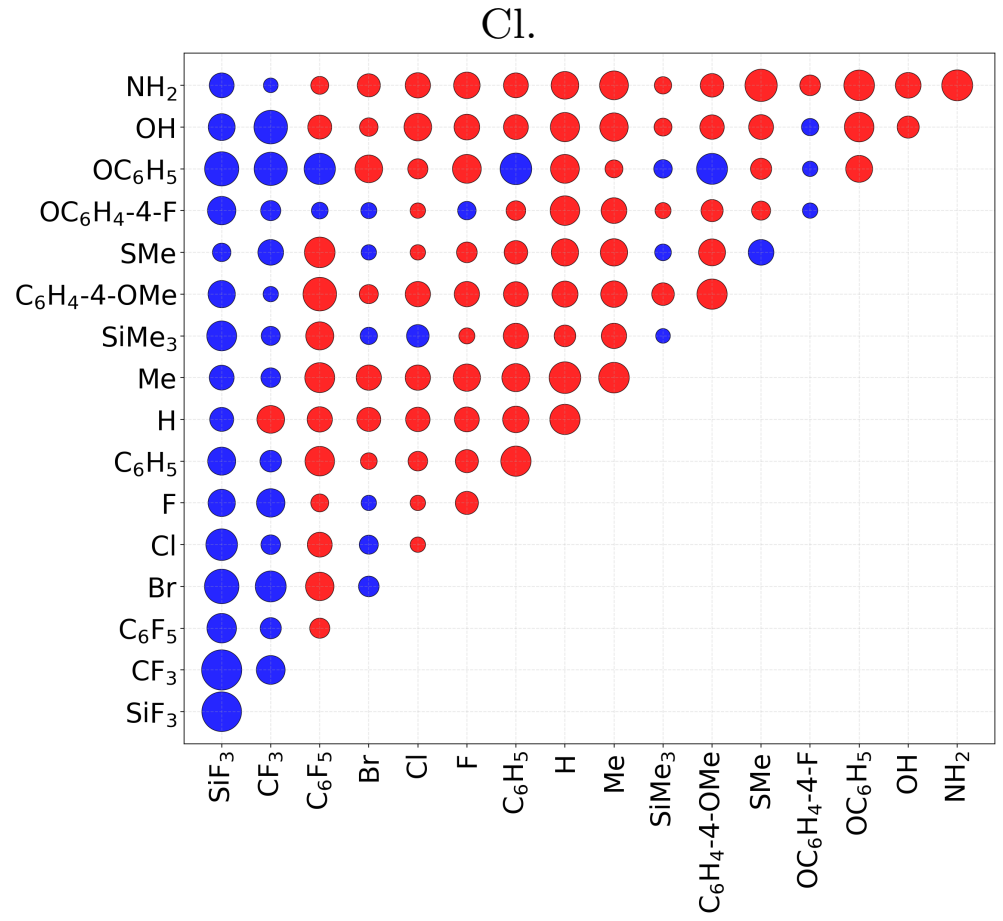

(c) Effect of substituents on the reaction energy difference, $\Delta\Delta G$ ($\Delta G_{\text{Cl}^-} - \Delta G_{\text{NH}_3}$), when R^3 = CF_3.

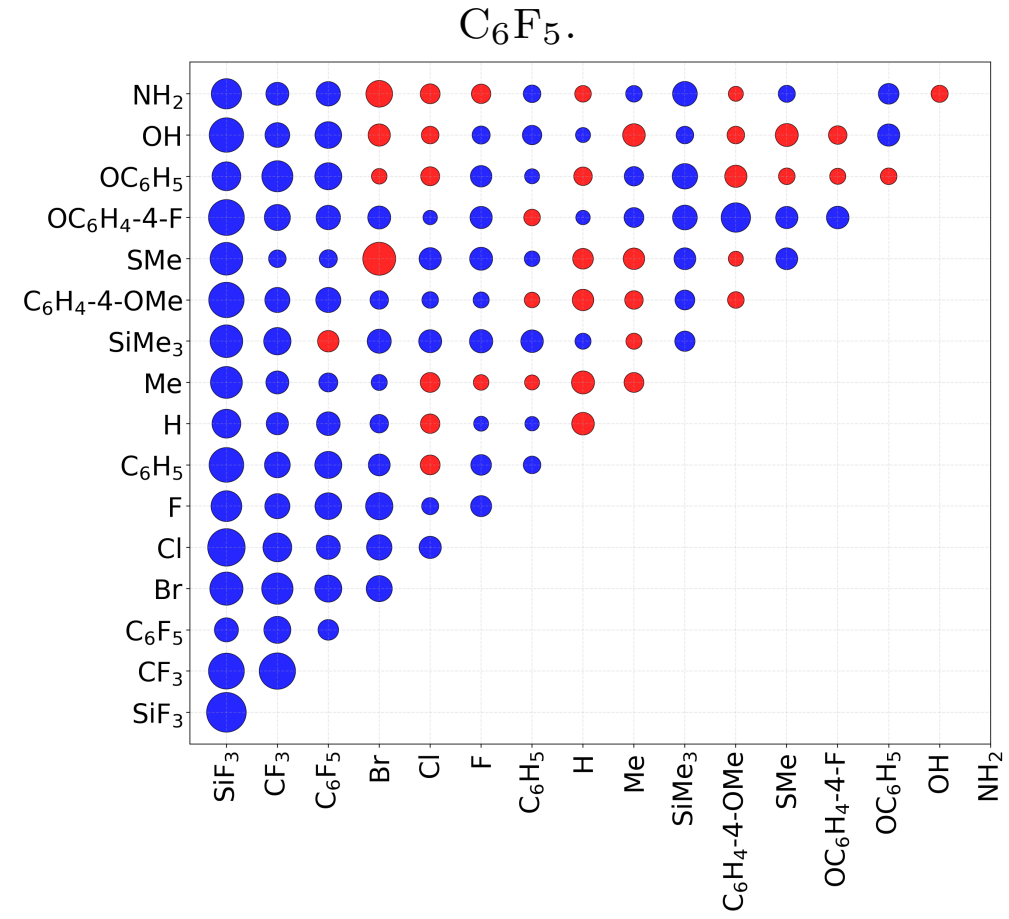

(d) Effect of substituents on the reaction energy difference, $\Delta\Delta G$ ($\Delta G_{\text{Cl}^-} - \Delta G_{\text{NH}_3}$), when R^3 = SiF_3.

Figure S13: Effect of substituents on the reaction energy difference, $\Delta\Delta G$ ($\Delta G_{\text{Cl}^-} - \Delta G_{\text{NH}_3}$). Positive $\Delta\Delta G$ values indicate a more favorable reaction with NH_3 as the Lewis base (represented by red circles), whereas negative $\Delta\Delta G$ values correspond to a greater stabilization with the Cl^- (represented by blue circles). The diameter of each circle is proportional to the magnitude of $\Delta\Delta G$, with smaller circles indicating minor differences in reaction energies between the two bases.

0.10. Substituent Effects on Relative Reaction Energies with Pyridine and NH_3

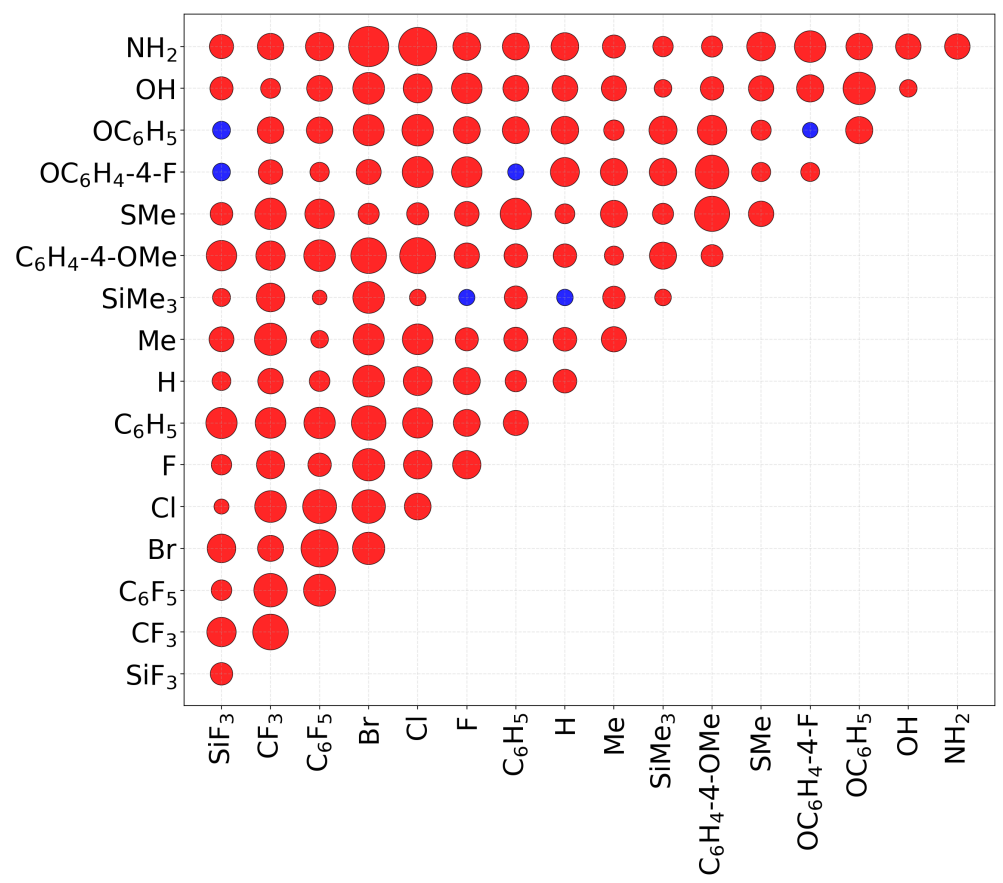

(a) Effect of substituents on the reaction energy difference, $\Delta\Delta G$ ($\Delta G_{\text{Pyridine}} - \Delta G_{NH_3}$), when R^3 = NH_2.

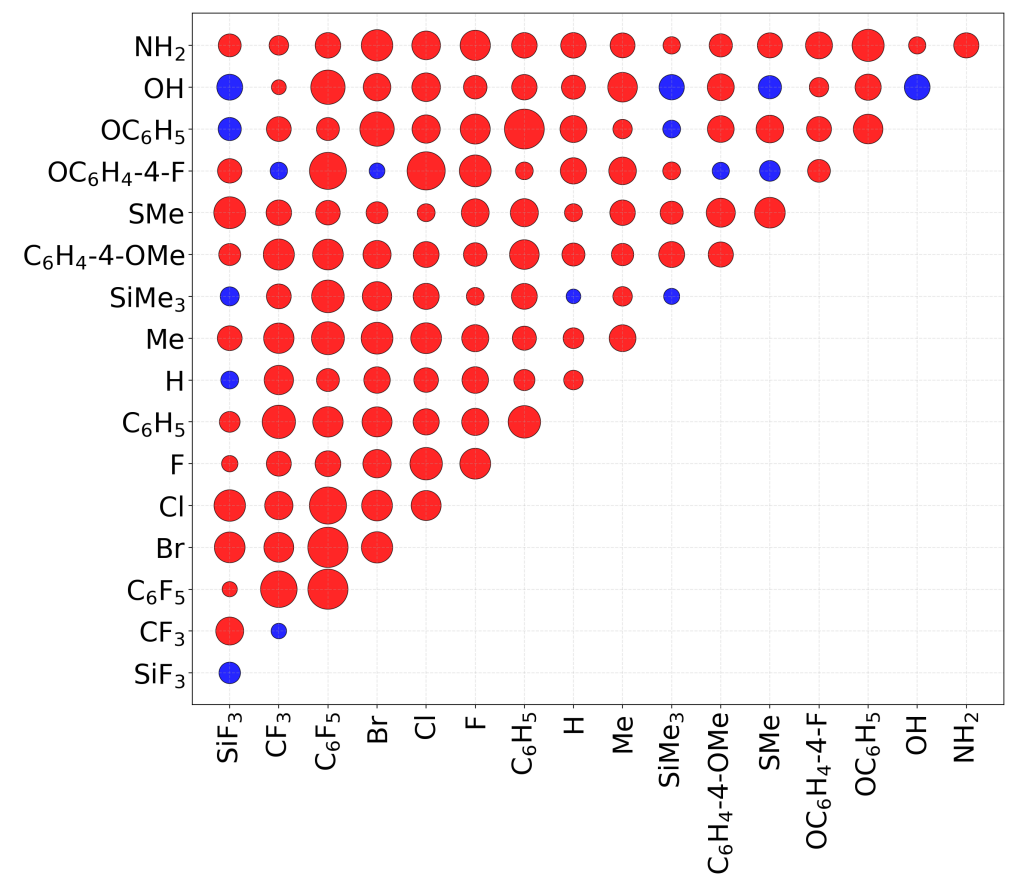

(b) Effect of substituents on the reaction energy difference, $\Delta\Delta G$ ($\Delta G_{\text{Pyridine}} - \Delta G_{NH_3}$), when R^3 = OH.

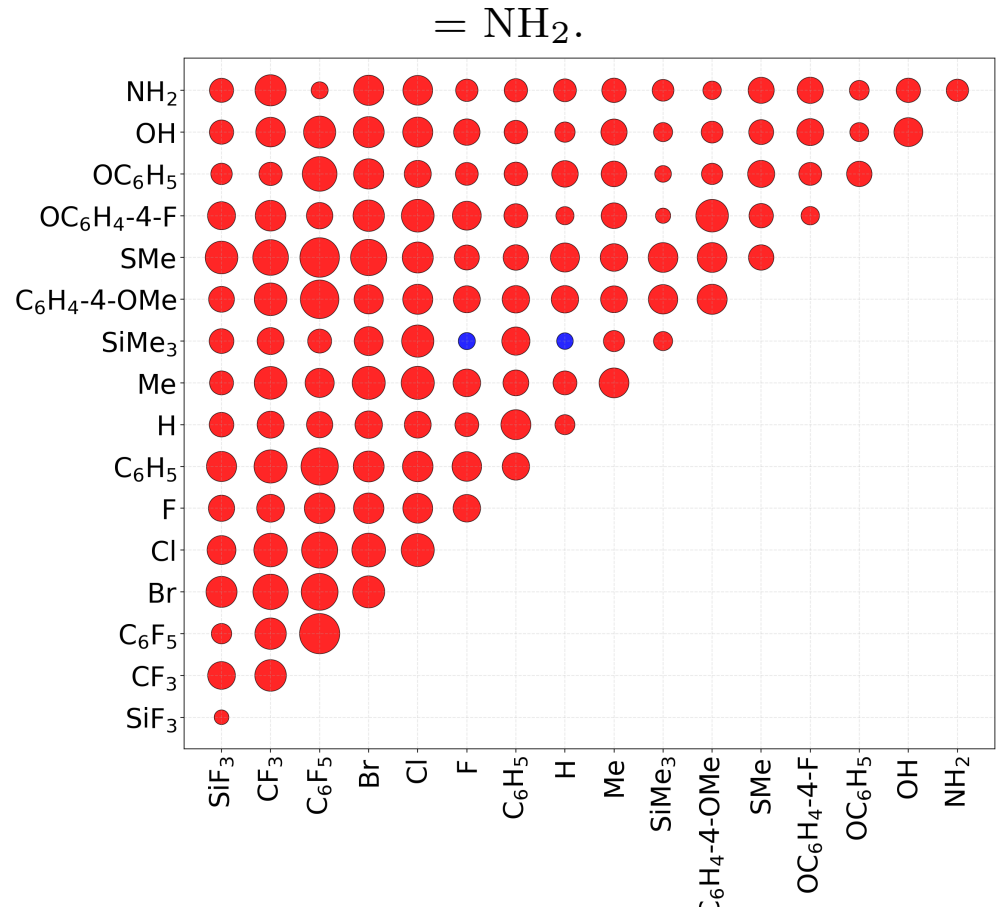

(c) Effect of substituents on the reaction energy difference, $\Delta\Delta G$ ($\Delta G_{\text{Pyridine}} - \Delta G_{NH_3}$), when R^3 = Me.

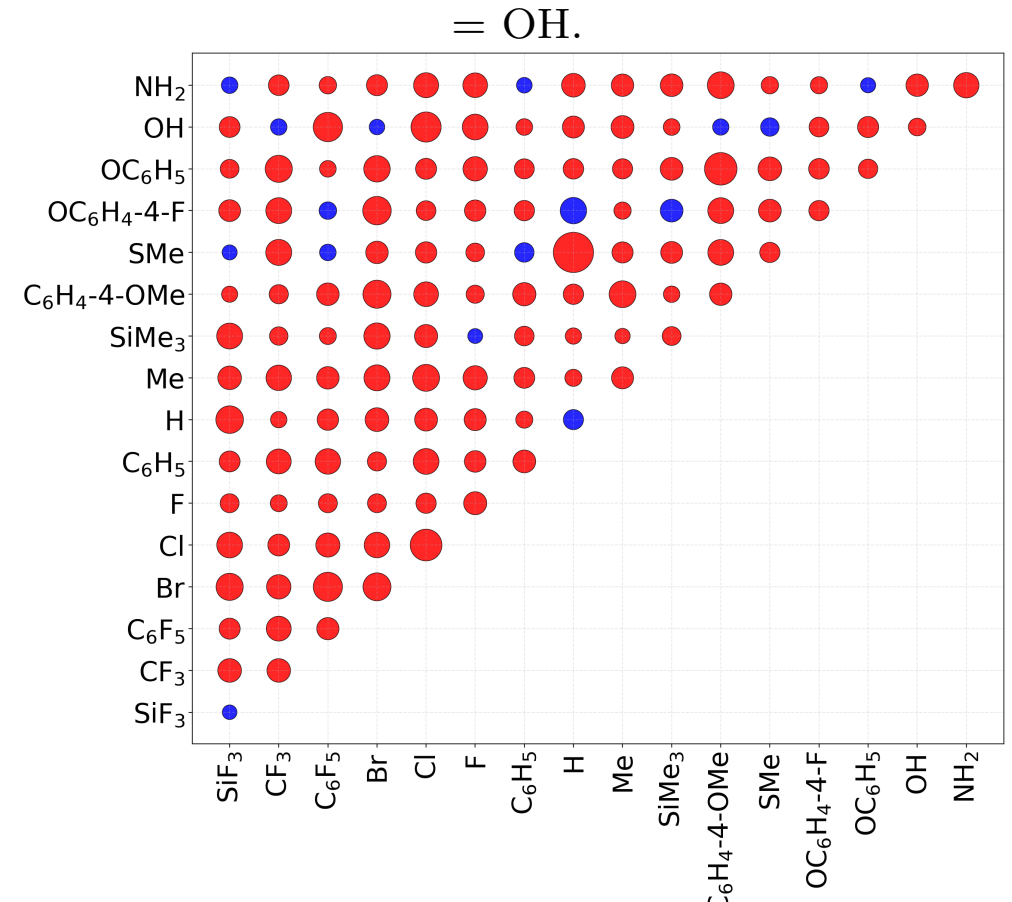

(d) Effect of substituents on the reaction energy difference, $\Delta\Delta G$ ($\Delta G_{\text{Pyridine}} - \Delta G_{NH_3}$), when R^3 = OC_6H_4-4-F.

Figure S14: Effect of substituents on the reaction energy difference, $\Delta\Delta G$ ($\Delta G_{\text{Pyridine}} - \Delta G_{NH_3}$). Positive $\Delta\Delta G$ values indicate a more favorable reaction with NH_3 as the Lewis base (represented by red circles), whereas negative $\Delta\Delta G$ values correspond to a greater stabilization with Pyridine (represented by blue circles). The diameter of each circle is proportional to the magnitude of $\Delta\Delta G$, with smaller circles indicating minor differences in reaction energies between the two bases.

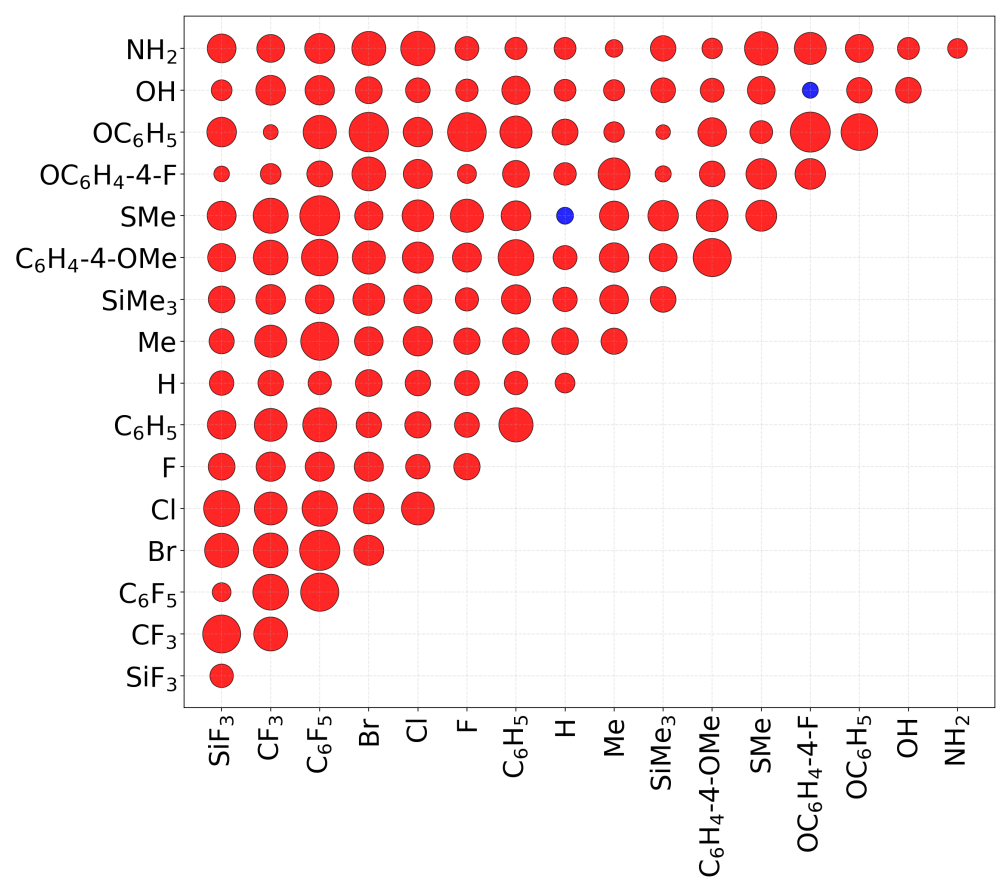

(a) Effect of substituents on the reaction energy difference, $\Delta\Delta G$ ($\Delta G_{\text{Pyridine}} - \Delta G_{\text{NH}_3}$), when R^3 = C_6H_4-4-OMe.

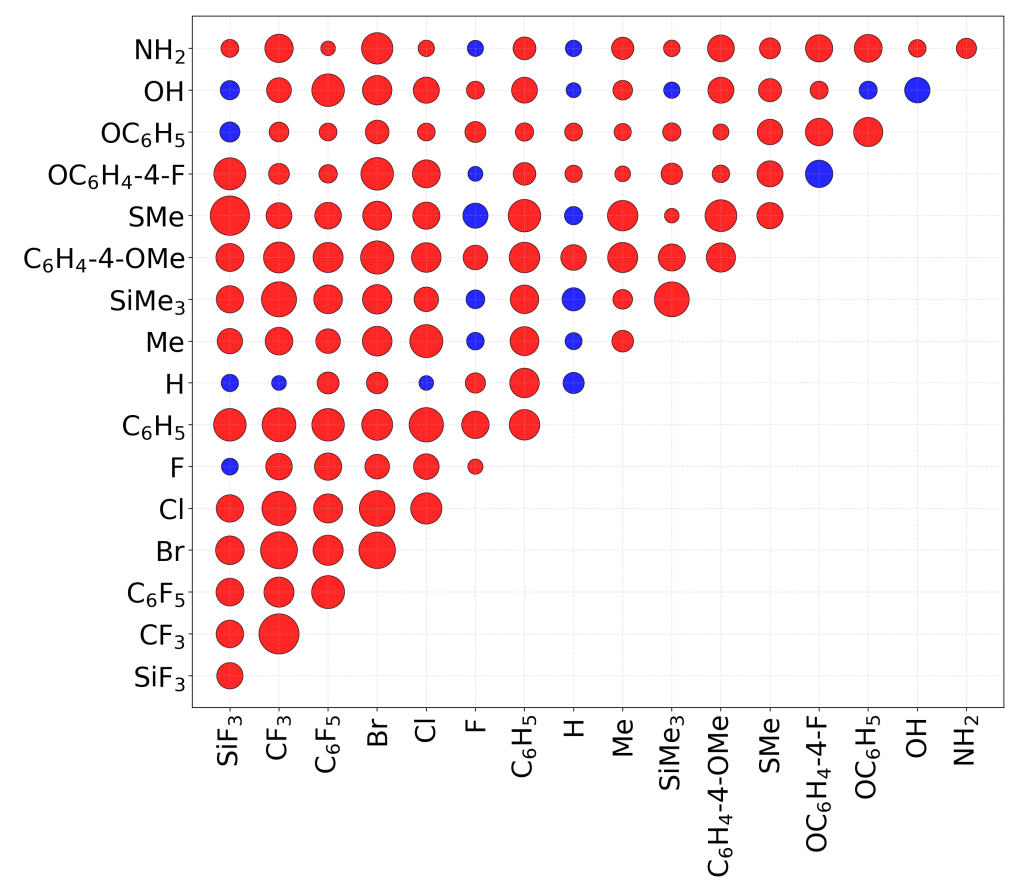

(b) Effect of substituents on the reaction energy difference, $\Delta\Delta G$ ($\Delta G_{\text{Pyridine}} - \Delta G_{\text{NH}_3}$), when R^3 = $SiMe_3$.

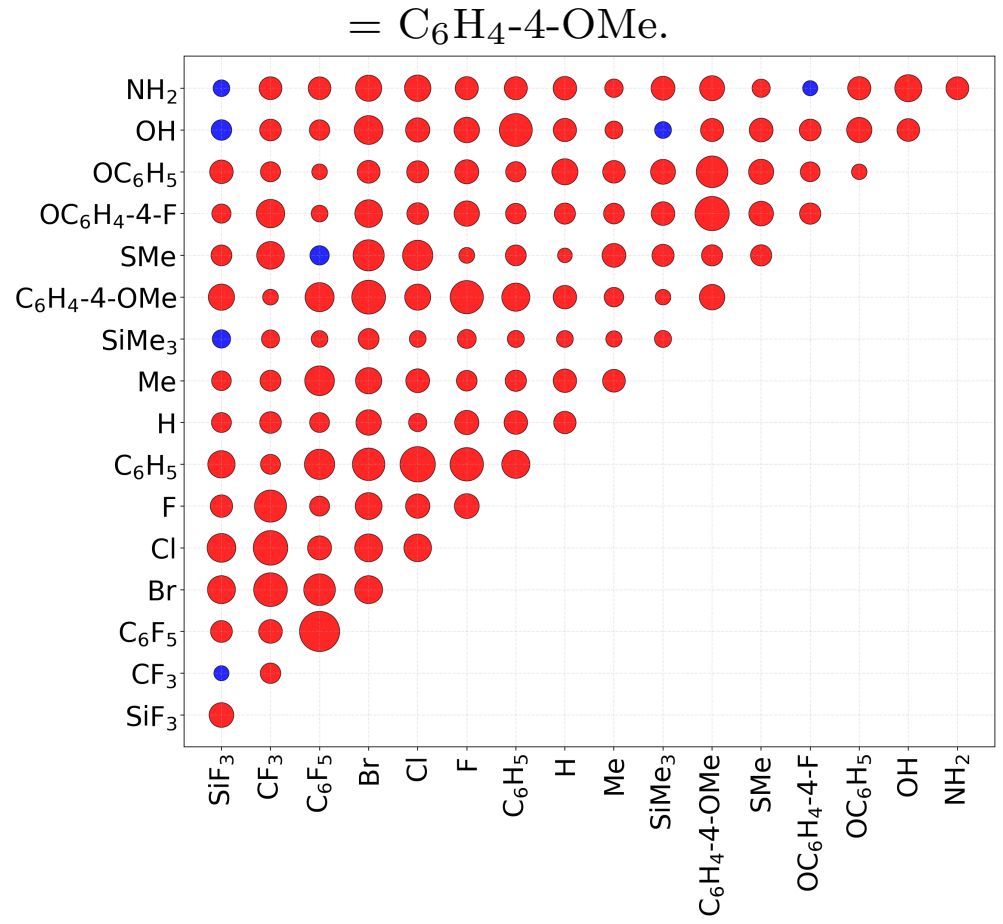

(c) Effect of substituents on the reaction energy difference, $\Delta\Delta G$ ($\Delta G_{\text{Pyridine}} - \Delta G_{\text{NH}_3}$), when R^3 = OC_6H_5.

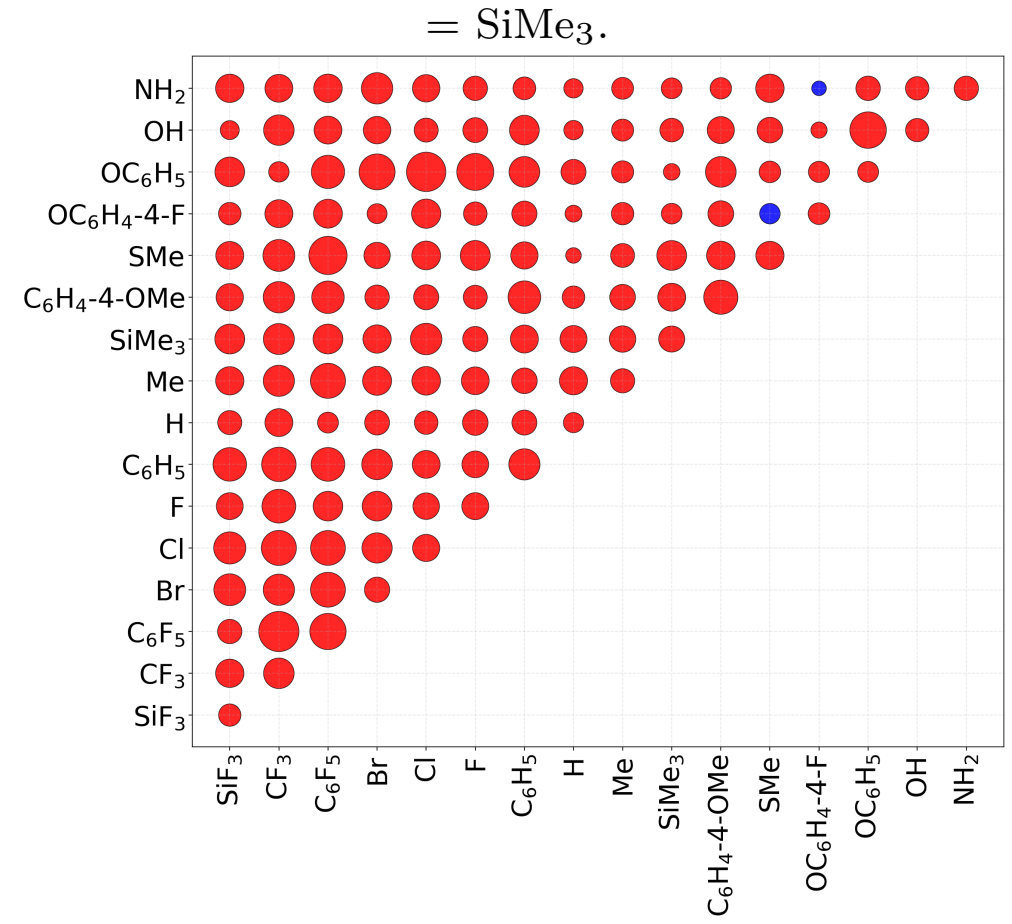

(d) Effect of substituents on the reaction energy difference, $\Delta\Delta G$ ($\Delta G_{\text{Pyridine}} - \Delta G_{\text{NH}_3}$), when R^3 = C_6H_5.

Figure S15: Effect of substituents on the reaction energy difference, $\Delta\Delta G$ ($\Delta G_{\text{Pyridine}} - \Delta G_{\text{NH}_3}$). Positive $\Delta\Delta G$ values indicate a more favorable reaction with NH_3 as the Lewis base (represented by red circles), whereas negative $\Delta\Delta G$ values correspond to a greater stabilization with the Pyridine (represented by blue circles). The diameter of each circle is proportional to the magnitude of $\Delta\Delta G$, with smaller circles indicating minor differences in reaction energies between the two bases.

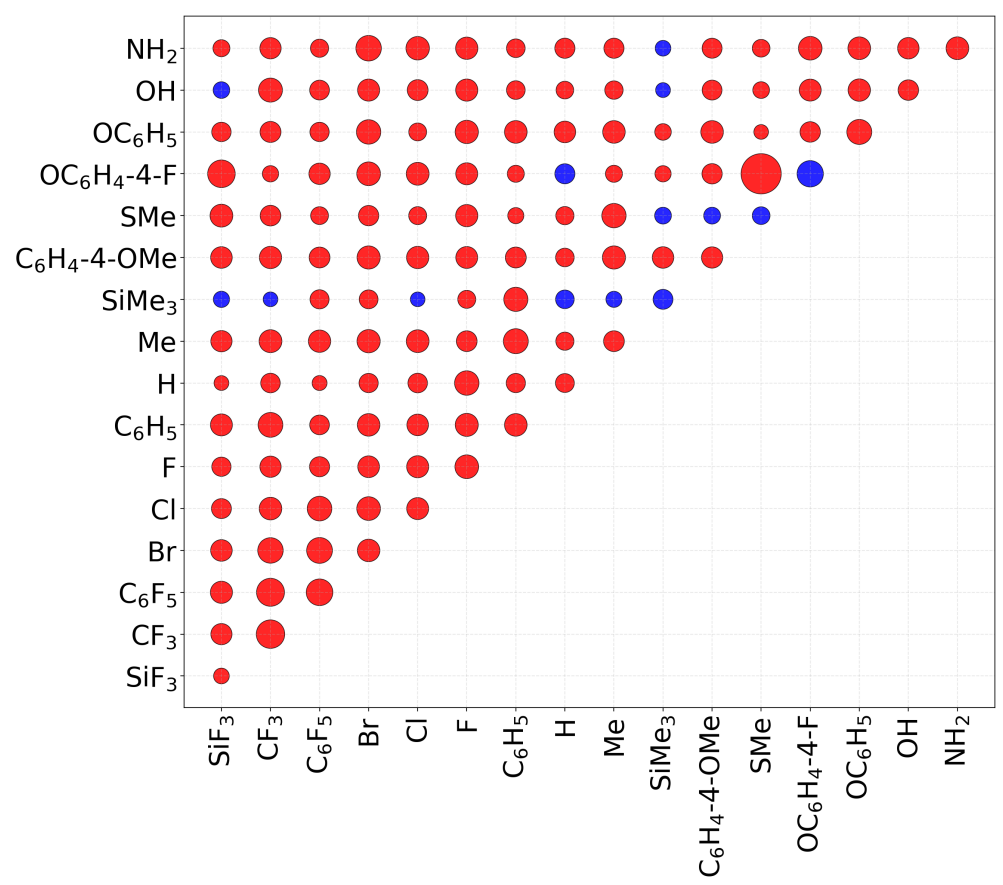

(a) Effect of substituents on the reaction energy difference, $\Delta\Delta G$ ($\Delta G_{\text{Pyridine}} - \Delta G_{\text{NH}_3}$), when R^3 = H.

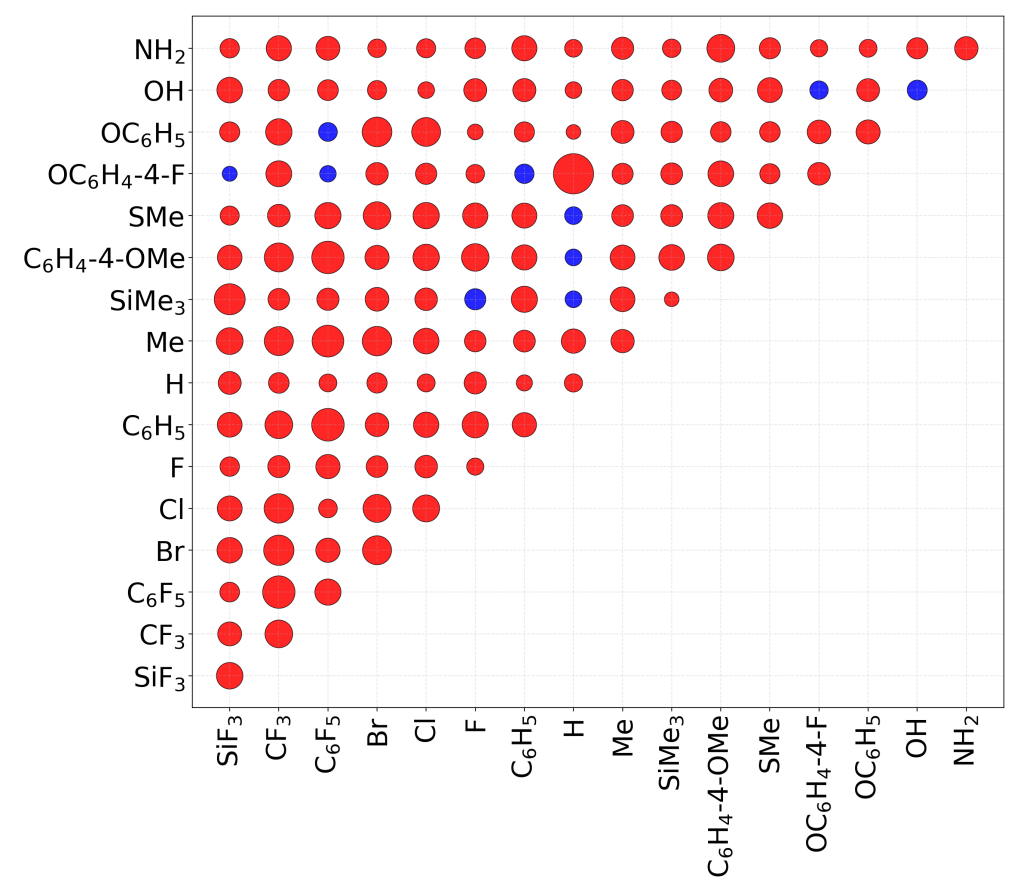

(b) Effect of substituents on the reaction energy difference, $\Delta\Delta G$ ($\Delta G_{\text{Pyridine}} - \Delta G_{\text{NH}_3}$), when R^3 = SMe.

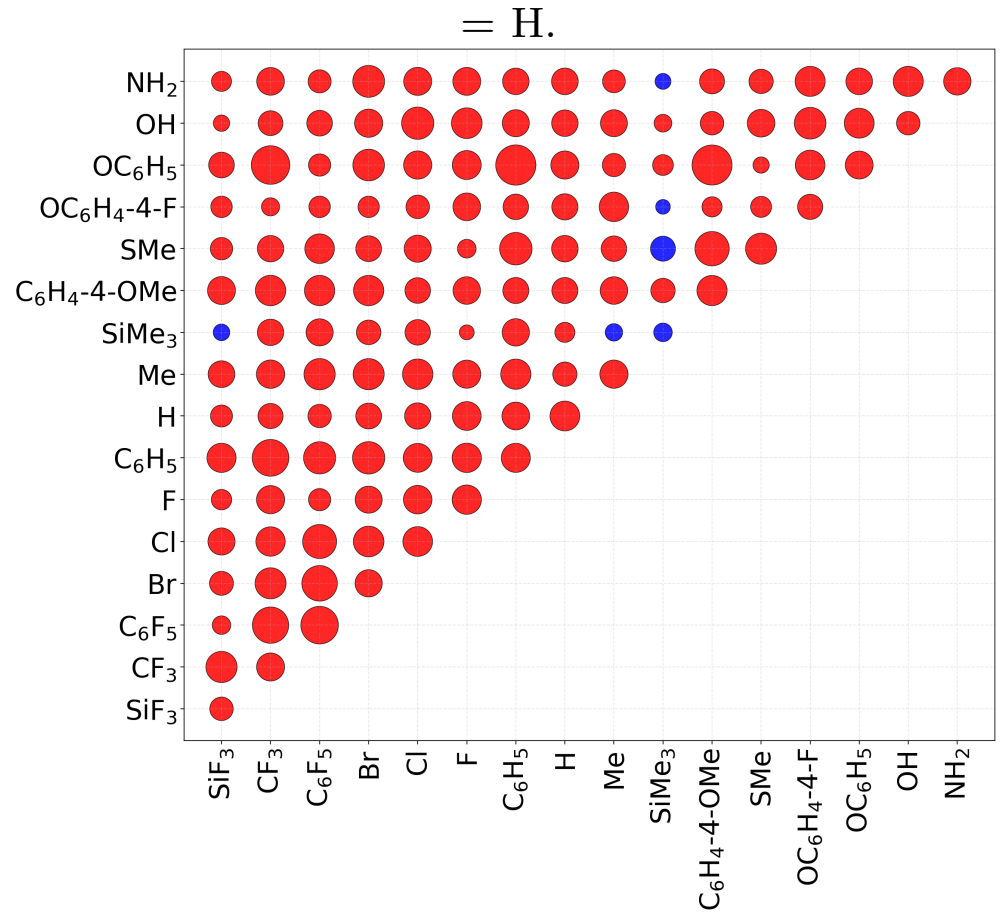

(c) Effect of substituents on the reaction energy difference, $\Delta\Delta G$ ($\Delta G_{\text{Pyridine}} - \Delta G_{\text{NH}_3}$), when R^3 =F.

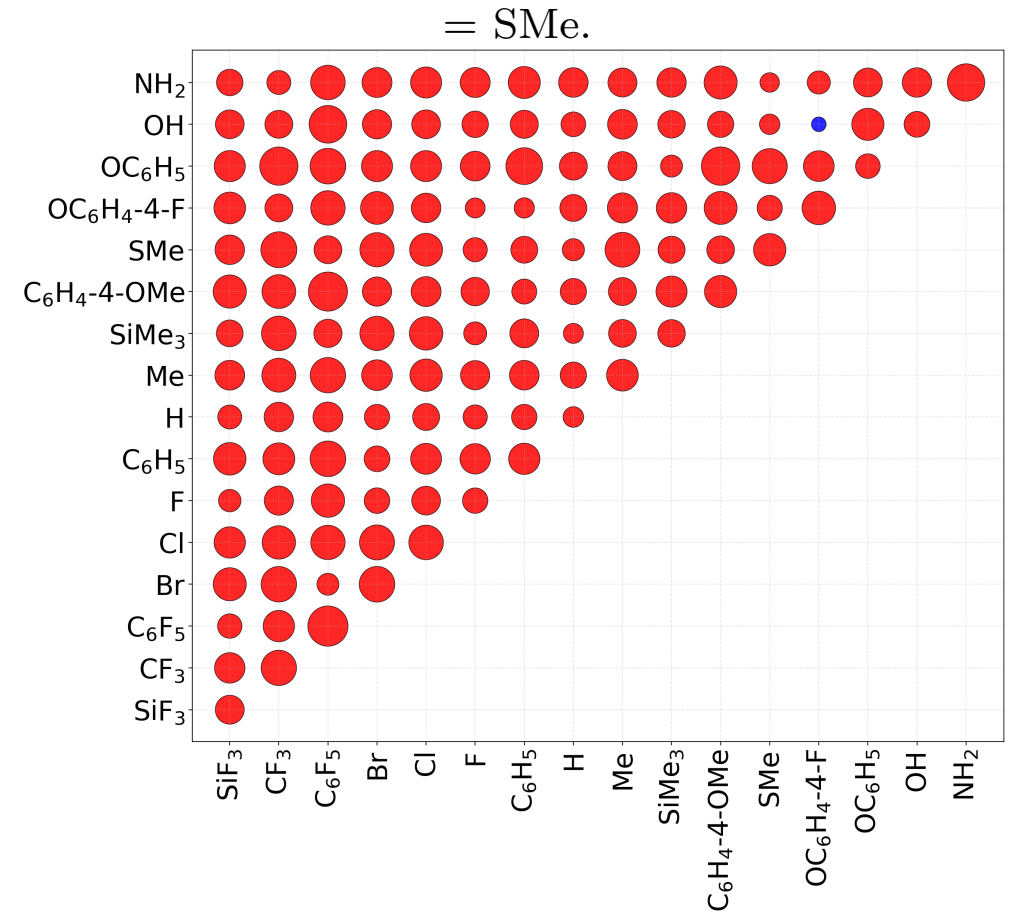

(d) Effect of substituents on the reaction energy difference, $\Delta\Delta G$ ($\Delta G_{\text{Pyridine}} - \Delta G_{\text{NH}_3}$), when R^3 = Br.

Figure S16: Effect of substituents on the reaction energy difference, $\Delta\Delta G$ ($\Delta G_{\text{Pyridine}} - \Delta G_{\text{NH}_3}$). Positive $\Delta\Delta G$ values indicate a more favorable reaction with NH_3 as the Lewis base (represented by red circles), whereas negative $\Delta\Delta G$ values correspond to a greater stabilization with the Pyridine (represented by blue circles). The diameter of each circle is proportional to the magnitude of $\Delta\Delta G$, with smaller circles indicating minor differences in reaction energies between the two bases.

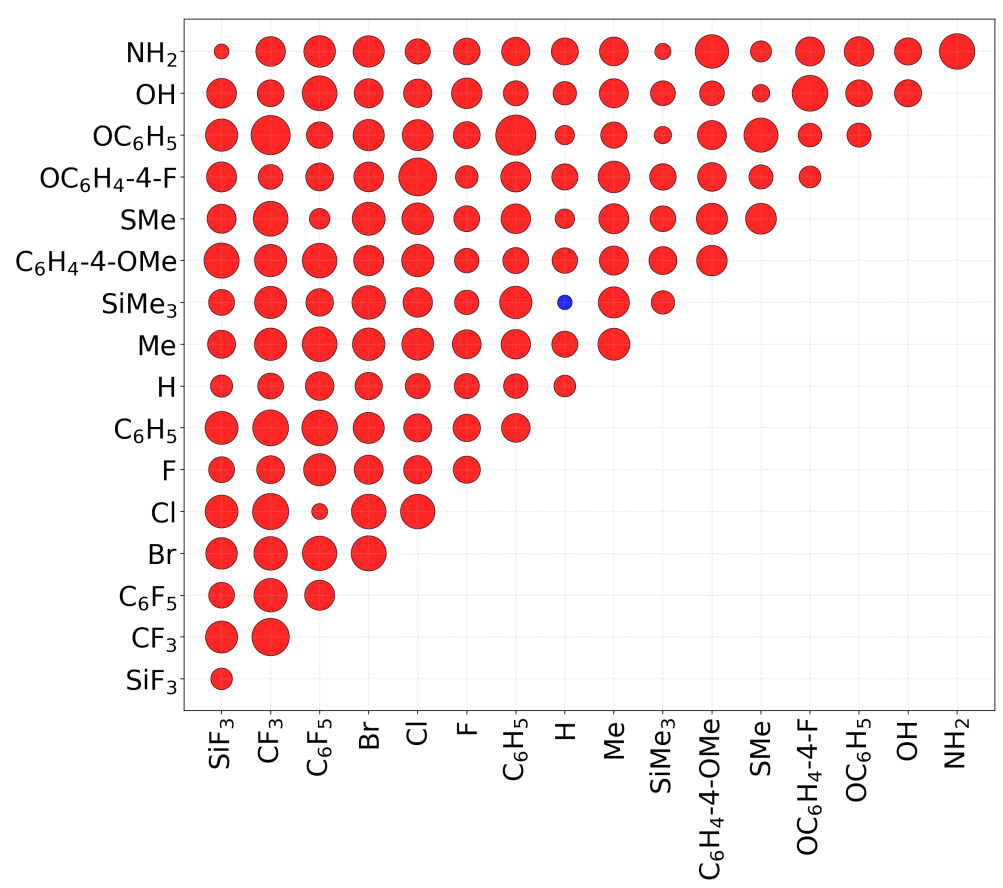

(a) Effect of substituents on the reaction energy difference, $\Delta\Delta G$ ($\Delta G_{\mathrm{Pyridine}} - \Delta G_{\mathrm{NH_3}}$), when R^3 = Cl.

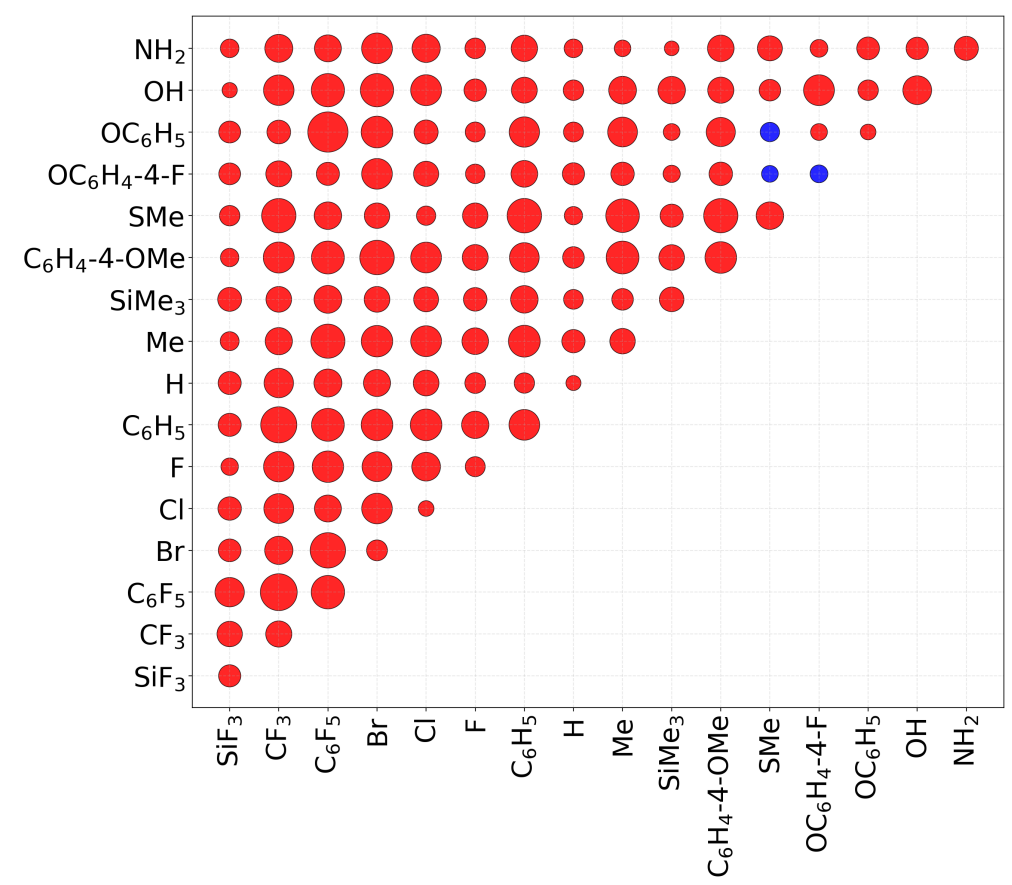

(b) Effect of substituents on the reaction energy difference, $\Delta\Delta G$ ($\Delta G_{\mathrm{Pyridine}} - \Delta G_{\mathrm{NH_3}}$), when R^3 = C_6F_5.

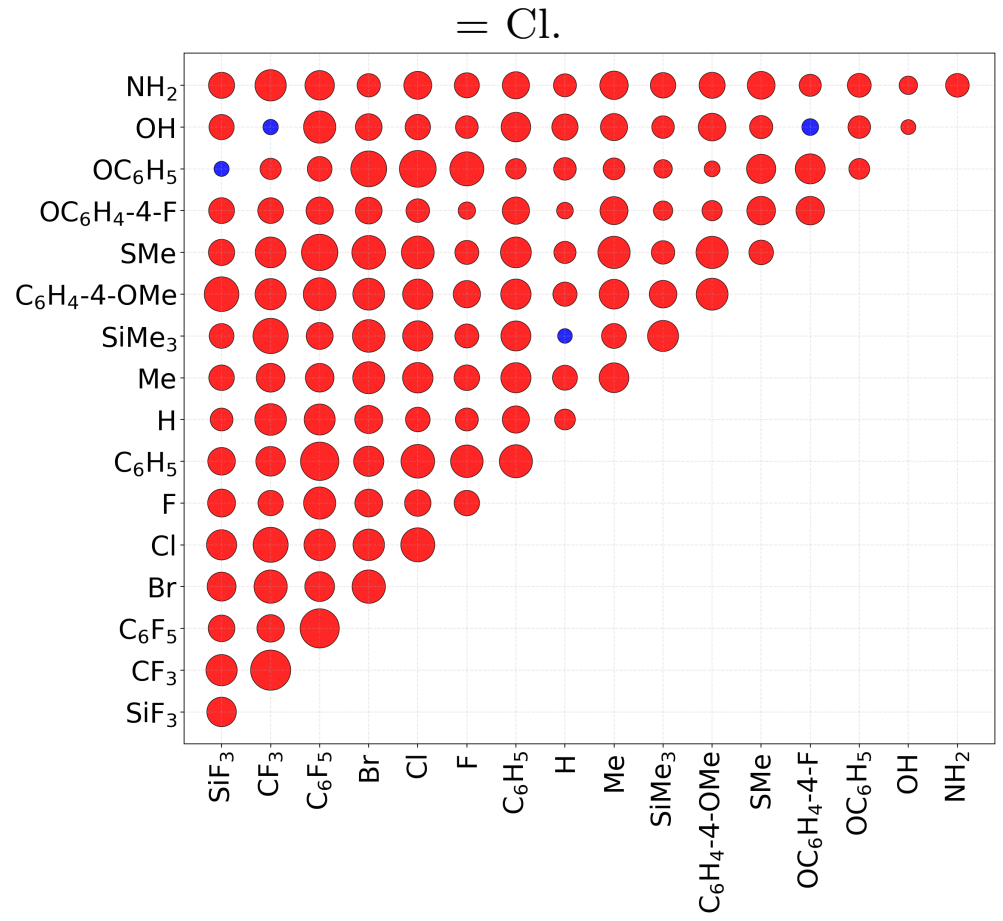

(c) Effect of substituents on the reaction energy difference, $\Delta\Delta G$ ($\Delta G_{\mathrm{Pyridine}} - \Delta G_{\mathrm{NH_3}}$), when R^3 = CF_3.

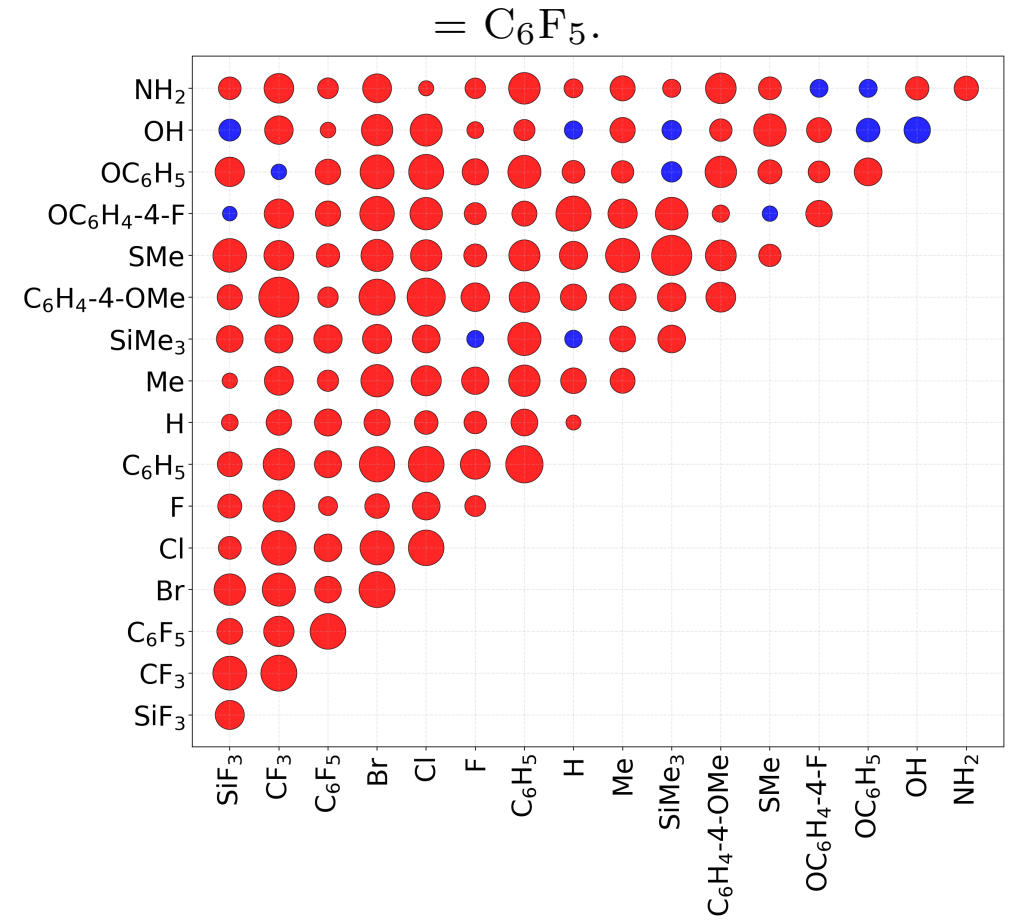

(d) Effect of substituents on the reaction energy difference, $\Delta\Delta G$ ($\Delta G_{\mathrm{Pyridine}} - \Delta G_{\mathrm{NH_3}}$), when R^3 = SiF_3.

Figure S17: Effect of substituents on the reaction energy difference, $\Delta\Delta G$ ($\Delta G_{\mathrm{Pyridine}} - \Delta G_{\mathrm{NH_3}}$). Positive $\Delta\Delta G$ values indicate a more favorable reaction with NH_3 as the Lewis base (represented by red circles), whereas negative $\Delta\Delta G$ values correspond to a greater stabilization with the Pyridine (represented by blue circles). The diameter of each circle is proportional to the magnitude of $\Delta\Delta G$, with smaller circles indicating minor differences in reaction energies between the two bases.

0.11. Linear Scaling Relationships between Hardness and Reaction Free Energy

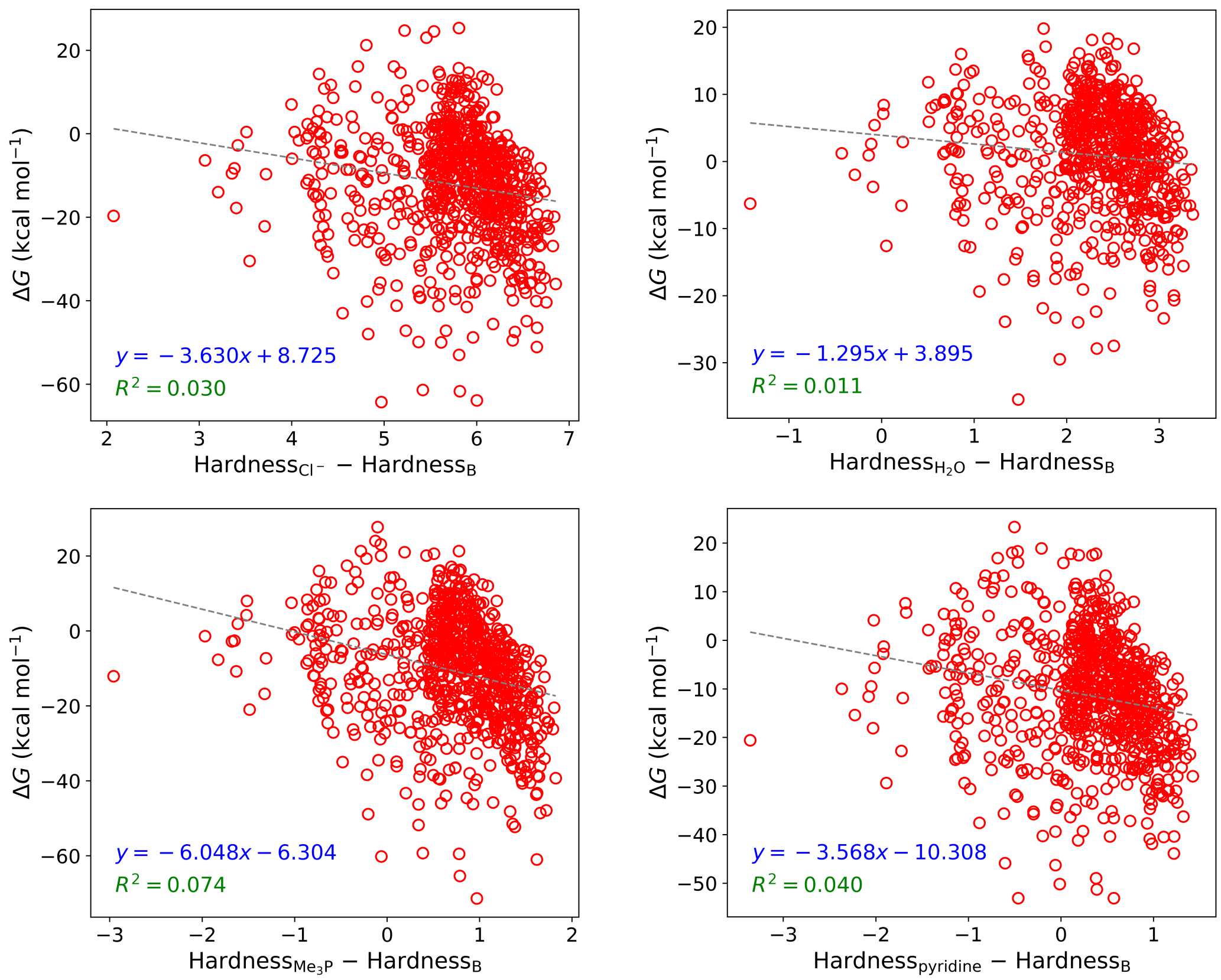

Figure S18: Linear scaling relationships between the hardness difference ($\eta_{\mathrm{Base}} - \eta_{\mathrm{B}}$) and the reaction free energy (ΔG) for four different Lewis bases. The regression equations and corresponding coefficients of determination (R^2) are shown in each panel.

0.12. Summary of Quantum Chemical Descriptors for Predictive Modeling

The 6 groups of electronic descriptors used for model training are summarized and described in detail in Table S10. The selected descriptors include the frontier molecular orbital energies of borane—namely the highest occupied molecular orbital (HOMO) and the lowest unoccupied molecular orbital (LUMO)—together with global reactivity indices derived from them: electrophilicity (ω), describing the tendency of a molecule to accept electron density; chemical potential (μ), indicating how readily a system can gain or lose electrons; and hardness (η), reflecting the resistance of a molecule to charge redistribution. Orbital interactions were further examined through the p_z orbital occupancies of the boron atoms directly involved in the reaction. To quantify substituent effects, we additionally employed the Hammett σ_p parameter, which provide insights into the electron-donating or -withdrawing character of substituents.

Table S10: Descriptors employed in ML approaches for modeling the reactivity of Borane derivatives.

**Name of descriptor**	**Explanation**
σ_p on Borane	A quantitative substituent constant describing the electron-donating or electron-withdrawing nature of groups attached to the Borane framework.
LUMO energy of Borane	Energy of the lowest unoccupied molecular orbital (E_LUMO), related to the electron-accepting ability of the Borane derivative.
HOMO energy of Borane	Energy of the highest occupied molecular orbital (E_HOMO), associated with the electron-donating capability of the Borane derivative.

Name of descriptor	Explanation
Electrophilicity index (ω)	A global reactivity descriptor quantifying the tendency of a Borane molecule to accept electron density: $$\omega = \frac{\mu^2}{2\eta}, \qquad \mu = -\frac{(I+A)}{2}, \qquad \eta = \frac{(I-A)}{2}.$$ Here, I and A are the ionization potential and electron affinity, typically approximated as $I \approx -E_{\mathrm{HOMO}}$ and $A \approx -E_{\mathrm{LUMO}}$.
Chemical potential (μ) of Borane derivatives	Describes the energetic tendency of a Borane system to exchange electrons with its environment: $$\mu = -\frac{(I+A)}{2} \approx \frac{E_{\mathrm{HOMO}} + E_{\mathrm{LUMO}}}{2}.$$
Electronic hardness (η) of Borane	Measures the intrinsic resistance of a Borane molecule to electron density redistribution: $$\eta = \frac{(I-A)}{2} \approx \frac{E_{\mathrm{LUMO}} - E_{\mathrm{HOMO}}}{2}.$$

0.13. Assessment of Machine Learning Approaches in Predicting Reaction Energies

This work evaluates a variety of machine learning algorithms—including linear regression, decision trees, support vector machines (SVM), k-nearest neighbors (KNN), gradient boosting (GB), and random forests (RF)—to predict reaction energies from a set of 11 electronic descriptors. Their predictive performance is outlined below, allowing a direct assessment of model applicability in describing borane reactivity.

R^3B-all base

Linear Regression: MAE: 5.43, R^2: 0.75
Decision Tree: MAE: 3.26, R^2: 0.89
Support Vector Machine: MAE: 4.12, R^2: 0.84
K-Nearest Neighbors: MAE: 3.45, R^2: 0.89
Gradient Boosting: MAE: 3.53, R^2: 0.88
Random Forest: MAE: 2.47 R^2: 0.94

Among the evaluated algorithms, RF regression delivered the highest predictive performance, as reflected by its elevated R^2 values and low mean absolute errors (MAEs). Consequently, this model was selected for in-depth analysis and interpretation in the main text.

R^3B-Cl^-

Linear Regression: MAE: 3.57, R^2: 0.88
Decision Tree: MAE: 3.55, R^2: 0.88
Support Vector Machine: MAE: 3.74, R^2: 0.85
K-Nearest Neighbors: MAE: 3.21, R^2: 0.90
Gradient Boosting: MAE: 2.56, R^2: 0.94
Random Forest: MAE: 2.75, R^2: 0.92

Among the evaluated algorithms, GB regression delivered the highest predictive performance, as reflected by its elevated R^2 values and low mean absolute errors (MAEs). Consequently, this model was selected for in-depth analysis and interpretation in the main text.

R^3B-OH_2

Linear Regression: MAE: 2.66, R^2: 0.82
Decision Tree: MAE: 3.51, R^2: 0.68
Support Vector Machine: MAE: 2.58, R^2: 0.77
K-Nearest Neighbors: MAE: 2.49, R^2: 0.82

Gradient Boosting: MAE: 2.14, R^2: 0.88

Random Forest: MAE: 2.39, R^2: 0.84

Among the evaluated algorithms, GB regression delivered the highest predictive performance, as reflected by its elevated R^2 values and low mean absolute errors (MAEs). Consequently, this model was selected for in-depth analysis and interpretation in the main text.

R^3B-NH_3

Linear Regression: MAE: 4.04, R^2: 0.85

Decision Tree: MAE: 4.02, R^2: 0.84

Support Vector Machine: MAE: 4.30, R^2: 0.79

K-Nearest Neighbors: MAE: 3.13, R^2: 0.90

Gradient Boosting: MAE: 2.60, R^2: 0.94

Random Forest: MAE: 2.93, R^2: 0.92

Among the evaluated algorithms, GB regression delivered the highest predictive performance, as reflected by its elevated R^2 values and low mean absolute errors (MAEs). Consequently, this model was selected for in-depth analysis and interpretation in the main text.

R^3B-PMe_3

Linear Regression: MAE: 4.50, R^2: 0.83

Decision Tree: MAE: 4.53, R^2: 0.82

Support Vector Machine: MAE: 4.34, R^2: 0.82

K-Nearest Neighbors: MAE: 3.71, R^2: 0.89

Gradient Boosting: MAE: 3.16, R^2: 0.92

Random Forest: MAE: 3.37, R^2: 0.91

Among the evaluated algorithms, GB regression delivered the highest predictive performance, as reflected by its elevated R^2 values and low mean absolute errors (MAEs). Consequently, this model was selected for in-depth analysis and interpretation in the main text.

R^3B-pyridine

Linear Regression: MAE: 3.92, R^2: 0.85

Decision Tree: MAE: 4.07, R^2: 0.82

Support Vector Machine: MAE: 4.15, R^2: 0.78

K-Nearest Neighbors: MAE: 3.19, R^2: 0.89

Gradient Boosting: MAE: 2.55, R^2: 0.93

Random Forest: MAE: 2.91, R^2: 0.91

Among the evaluated algorithms, GB regression delivered the highest predictive performance, as reflected by its elevated R^2 values and low mean absolute errors (MAEs). Consequently, this mode was selected for in-depth analysis and interpretation in the main text.

Descriptor Correlation Analysis. Pairwise Pearson correlation coefficients were calculated for the five descriptors used in the SHAP analysis, and the resulting matrix is presented in Table S11. The largest absolute correlation coefficient is $|r| = 0.83$, indicating substantial correlation between some descriptors. A pairwise correlation threshold alone does not establish the absence of multicollinearity or nonlinear dependence. Moreover, some descriptors share mathematical dependencies through their definitions in terms of frontier orbital energies. The SHAP contributions should therefore be interpreted with these dependencies in mind, rather than as independent physical contributions to adduct formation.

Table S11: Pearson correlation matrix of all molecular descriptors used in SHAP analysis.

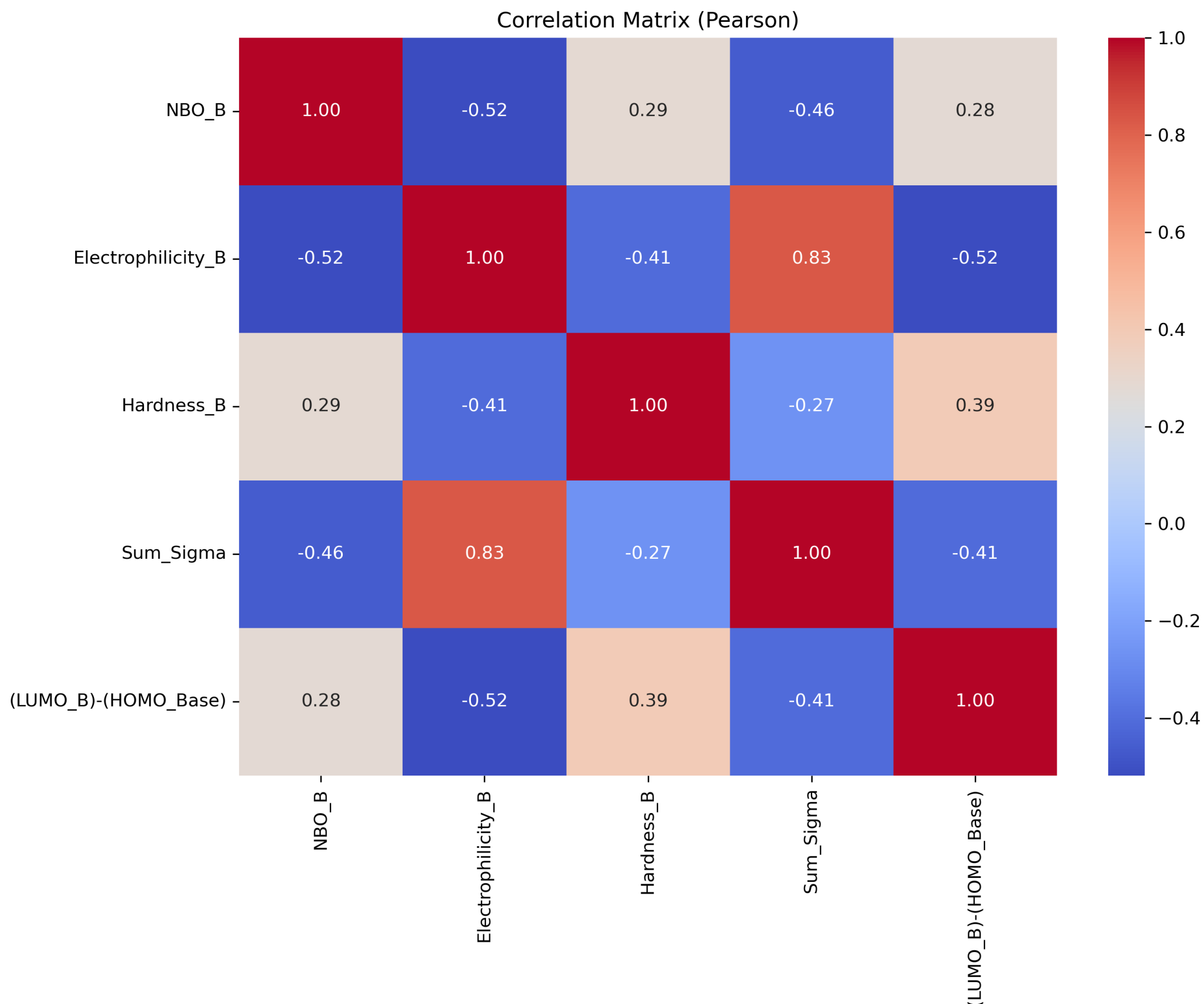

	NBO_B	Electrophilicity_B	Hardness_B	Sum_Sigma	(LUMO_B)-(HOMO_Base)
NBO_B	1.00	-0.52	0.29	-0.46	0.28
Electrophilicity_B	-0.52	1.00	-0.41	0.83	-0.52
Hardness_B	0.29	-0.41	1.00	-0.27	0.39
Sum_Sigma	-0.46	0.83	-0.27	1.00	-0.41
(LUMO_B)-(HOMO_Base)	0.28	-0.52	0.39	-0.41	1.00

0.14. Scatter Plots and SHAP Analysis of DFT Reaction Energies versus Machine Learning Predictions for Different Lewis Bases

After evaluating several machine learning algorithms, as detailed in Section S14 of the Supporting Information, gradient boosting regression was selected for training the models across all five datasets corresponding to the different Lewis bases. The resulting models exhibited strong predictive performance, with training R^2 values exceeding 0.90 for all bases and validation R^2 scores above 0.80 in every case.

Consistent with these observations, SHAP analysis performed across all Lewis bases identifies the σ_p parameters and $\text{Electrophilicity}_{\text{Borane}}$ as the two most dominant features governing the reaction energetics. Beyond these primary descriptors, $\text{HOMO}_{\text{Borane}}$, the $\text{LUMO}_{\text{Borane}}$–$\text{HOMO}_{\text{Base}}$ energy gap, $\text{Hardness}_{\text{Borane}}$, and $\text{NBO}_{\text{Borane}}$ follow in descending order of importance. Notably, Me_3P represents an exception to this trend, for which $\text{NBO}_{\text{Borane}}$ exerts a stronger influence than both $\text{HOMO}_{\text{Borane}}$ and the $\text{LUMO}_{\text{Borane}}$–$\text{HOMO}_{\text{Base}}$ gap.

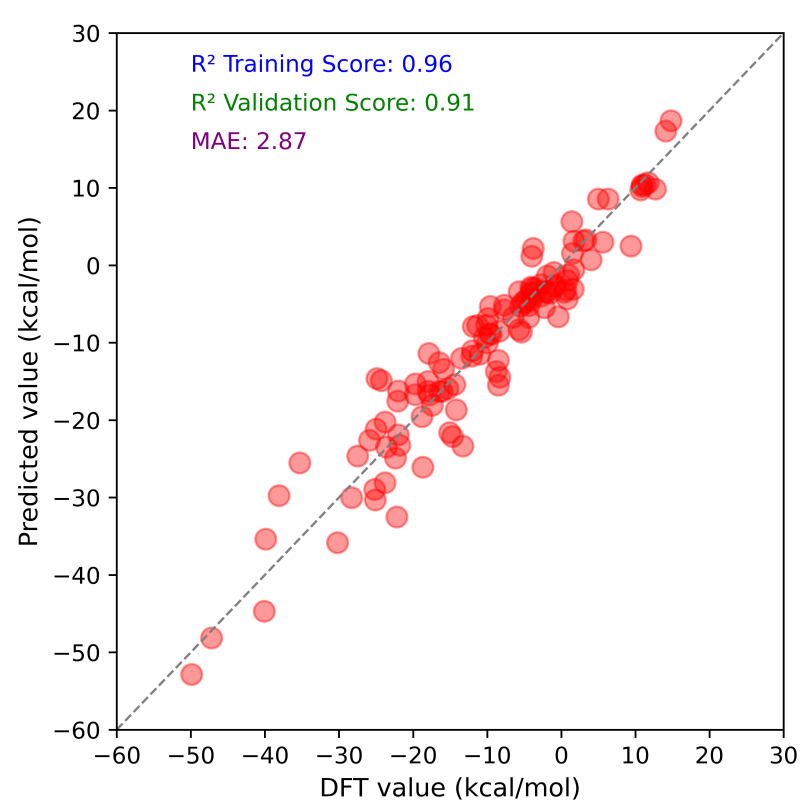

(a)

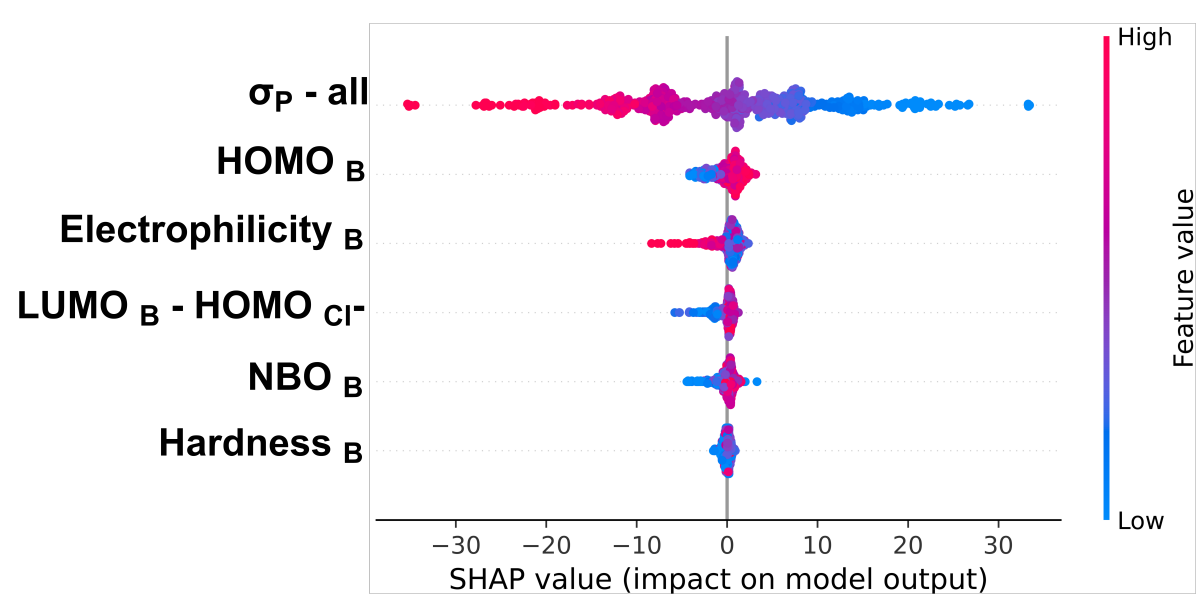

(b)

Figure S19: Scatter plot of DFT-computed ΔG_{rxn} versus predicted values from the gradient boosting (GB) regression model for Cl^-. (b) SHAP values (kcal mol^{-1}) showing the contribution of individual features across all samples. The color scale represents the feature magnitude, with blue indicating low values and red indicating high values.

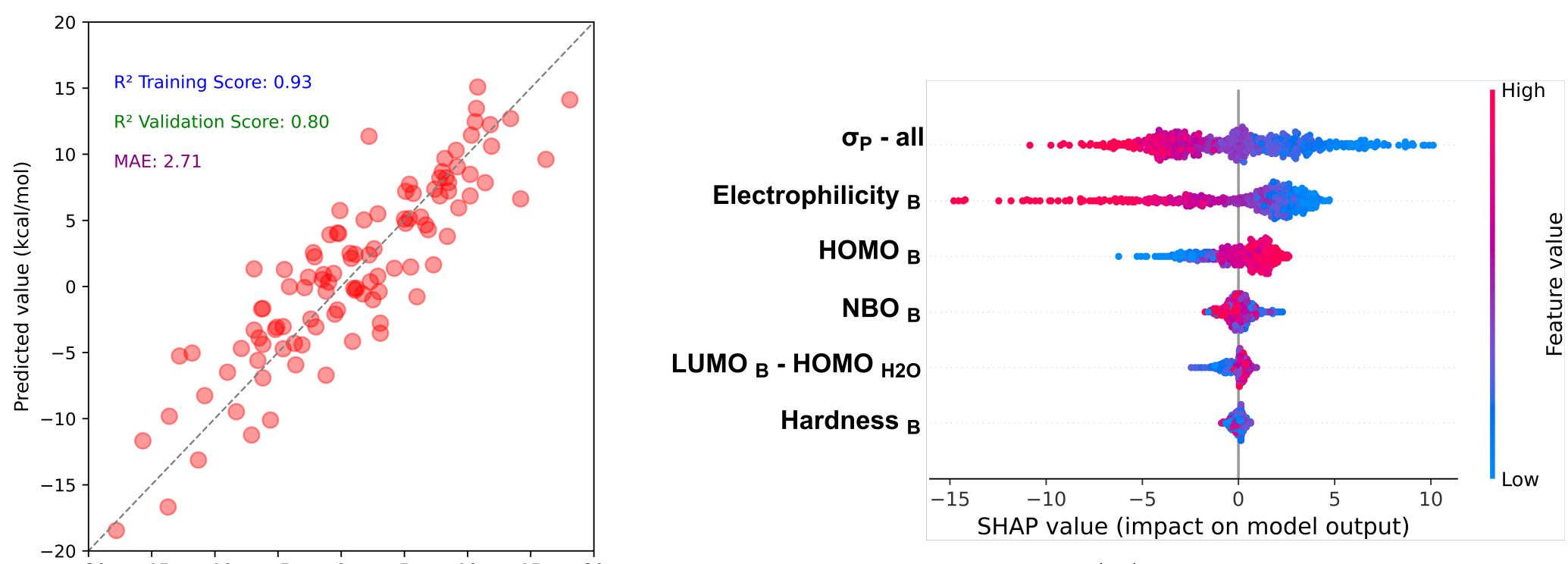

(a) (b)

Figure S20: (a) Scatter plot of DFT-computed ΔG_{rxn} versus predicted values from the gradient boosting (GB) regression model for H_2O. (b) SHAP values (kcal mol^{-1}) showing the contribution of individual features across all samples. The color scale represents the feature magnitude, with blue indicating low values and red indicating high values.

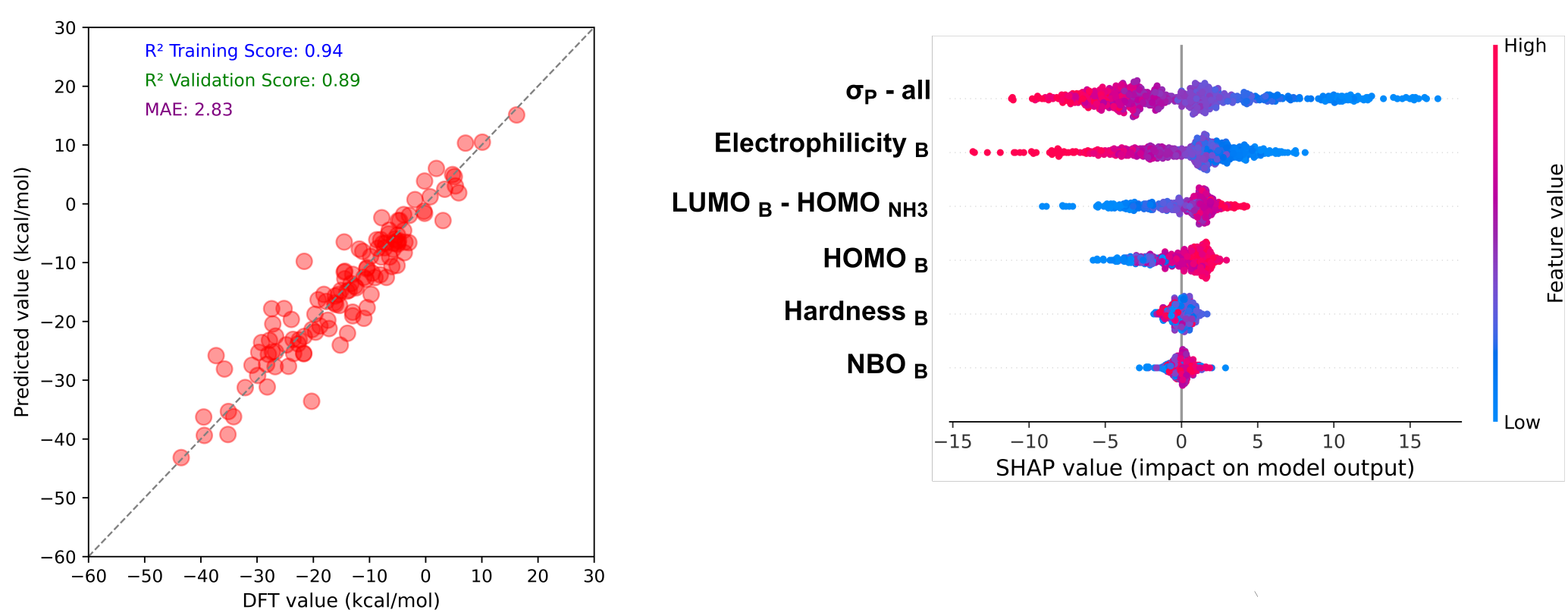

(a) (b)

Figure S21: (a) Scatter plot of DFT-computed ΔG_{rxn} versus predicted values from the gradient boosting (GB) regression model for NH_3. (b) SHAP values (kcal mol^{-1}) showing the contribution of individual features across all samples. The color scale represents the feature magnitude, with blue indicating low values and red indicating high values.

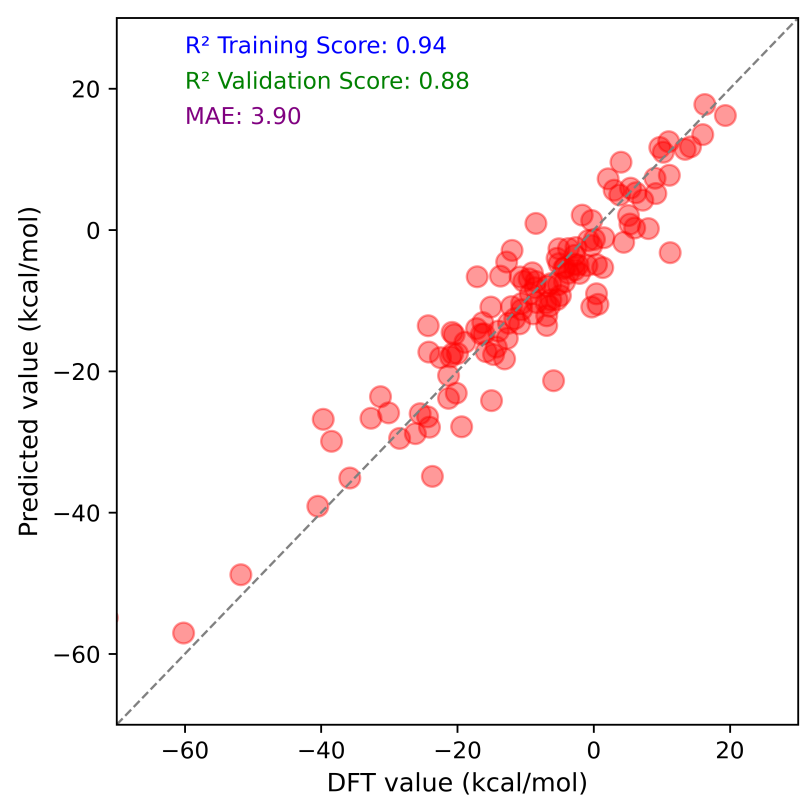

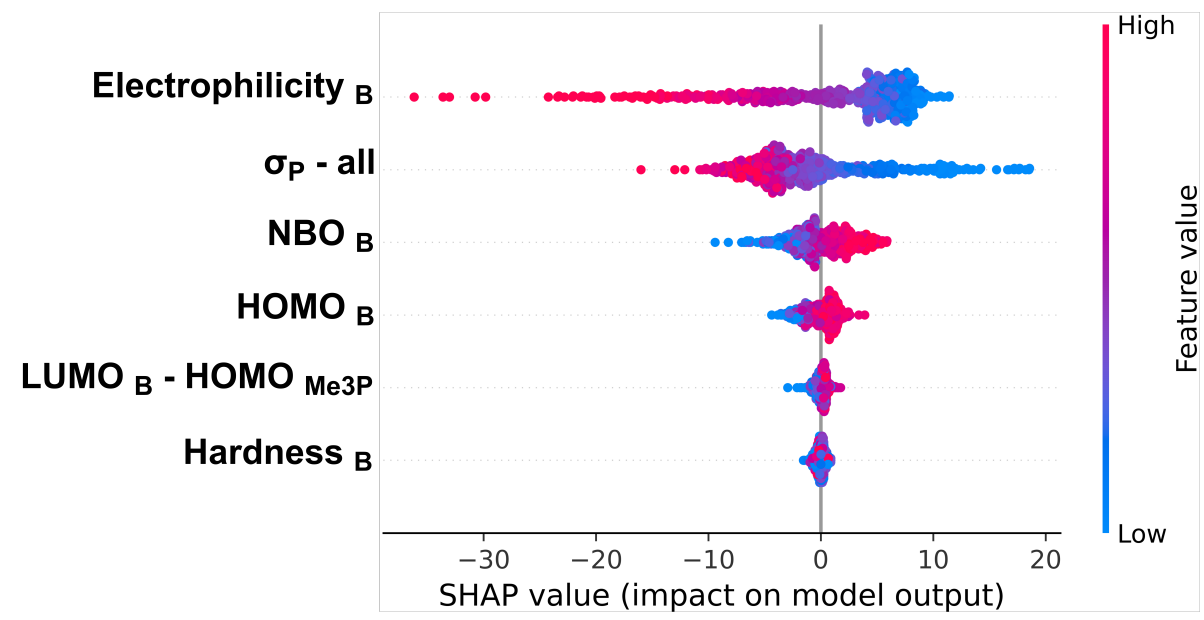

(b)

(a)

Figure S22: (a) Scatter plot of DFT-computed ΔG_{rxn} versus predicted values from the gradient boosting (GB) regression model for Me_3P. (b) SHAP values (kcal mol^{-1}) showing the contribution of individual features across all samples. The color scale represents the feature magnitude, with blue indicating low values and red indicating high values.

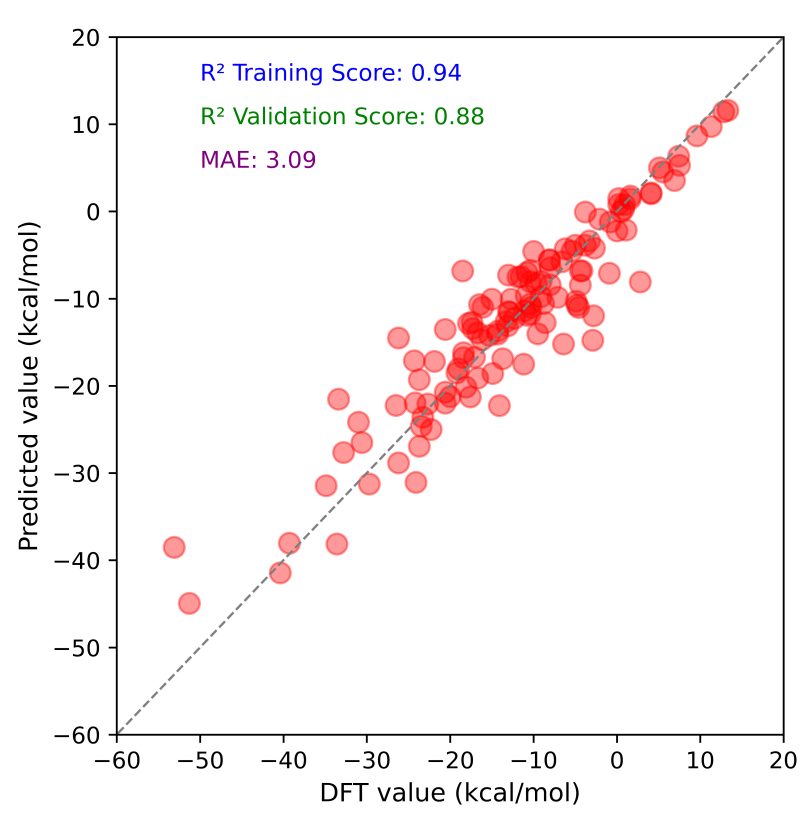

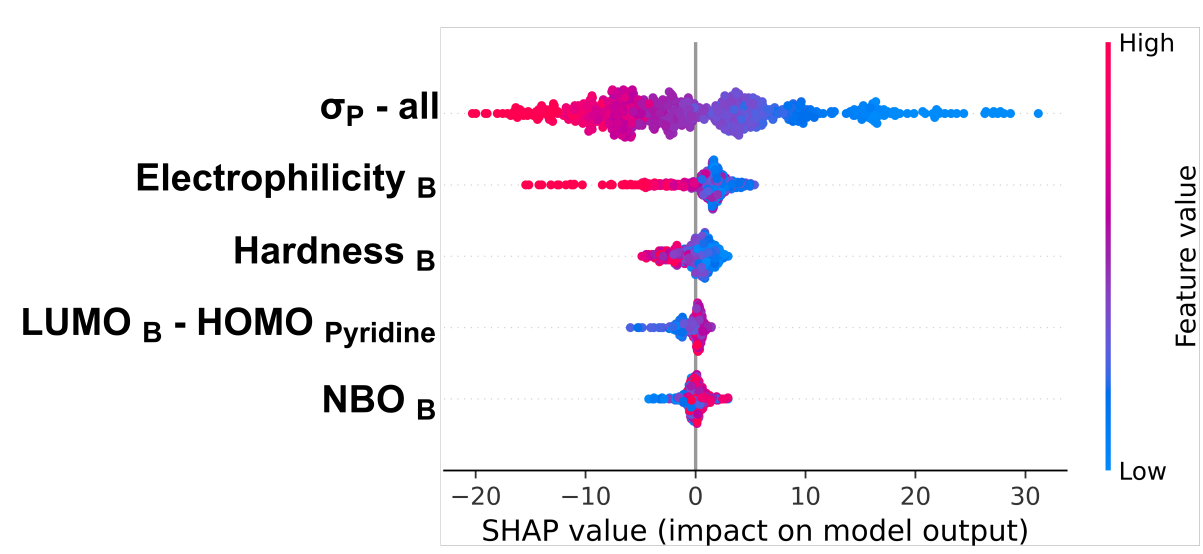

(b)

(a)

Figure S23: (a) Scatter plot of DFT-computed ΔG_{rxn} versus predicted values from the gradient boosting (GB) regression model for pyridine. (b) SHAP values (kcal mol^{-1}) showing the contribution of individual features across all samples. The color scale represents the feature magnitude, with blue indicating low values and red indicating high values.
